# Hallmarks of epithelial-mesenchymal plasticity in cancer

**DOI:** 10.1186/s12943-026-02690-x

**Published:** 2026-05-25

**Authors:** Laurie Clauzon, Francesco Verona, Kimiya Shams, Ornella Roberta Brancato, Sebastiano Di Bella, Noemi Di Miceli, Giorgio Stassi, Simone Di Franco

**Affiliations:** 1 Department of Precision Medicine in Medical, Surgical and Critical Care, University of Palermo, Palermo, 90127 Italy; 2 Department of Health Promotion Sciences, Internal Medicine and Medical Specialties (PROMISE), University of Palermo, Palermo, 90127 Italy; 3 IRCCS SYNLAB SDN, Naples, 80143 Italy

## Abstract

Cancer stem cells (CSCs) drive tumour initiation, progression, metastasis, and therapy resistance through their remarkable plasticity, enabling dynamic transitions between stem-like and differentiated states. A pivotal mechanism underlying this plasticity is epithelial-mesenchymal plasticity (EMP), which encompasses epithelial-mesenchymal transition (EMT), partial or hybrid EMT (E/M) states, and mesenchymal-epithelial transition (MET), allowing cancer cells to acquire invasive, stem-like properties while maintaining proliferative potential. Unlike the traditional binary view of EMT, recent evidence reveals a spectrum of intermediate E/M phenotypes that exhibit increased tumorigenicity, metastatic potential, and therapy resistance. This plasticity is orchestrated by intricate regulatory networks involving EMT-inducing transcription factors, signalling pathways, and non-coding RNAs. The tumour microenvironment (TME), with its cellular and non-cellular components, provides critical extrinsic cues that stabilize E/M states. Notably, metabolic reprogramming cooperates with EMP. Indeed, E/M flexibly shifts between glycolysis, oxidative phosphorylation, and lipid metabolism alterations to fuel invasion, buffer oxidative stress, and evade ferroptosis. Advanced and recently developed in vitro and in vivo models have illuminated these dynamics: dual-fluorescent reporters, microfluidic tumour-on-a-chip, genetically engineered mouse models, bioluminescence imaging, and intravital microscopy enable real-time tracking of EMP during progression and therapy response. On the other side, in silico tools, single-cell/spatial transcriptomics, network inference, machine learning, and agent-based modelling, map hybrid states, predict trajectories, and help identify biomarkers, revealing EMP’s role in evolutionary fitness. Therapeutically, targeting EMP holds promise to target resistant cancer cells and prevent relapse, though challenges arise from redundancy and plasticity. Strategies include pathway inhibitors, metabolic disruptors, epigenetic agents, TME modulators, and differentiation inducers. Combination therapies, guided by EMP biomarkers and rational models, act in combination with standard treatments to lock cells in epithelial states, disrupt hybrid phenotypes, and overcome resistance. This review highlights EMP as the main driver of tumour evolution, offering a unified framework for understanding tumour heterogeneity and heterogeneity-driven failures in therapy. By elucidating molecular mechanisms and vulnerabilities, it paves the way for precision interventions that could transform outcomes in aggressive malignancies, ultimately restraining metastasis and recurrence.

## Introduction

In this review, we deepen the EMP from its molecular foundations to its therapeutic implications. We first explore the intimate connection between CSCs and EMP, highlighting how dynamic transitions between stem-like and differentiated states, spanning classical EMT, its reverse MET, and intermediate E/M states, drive tumorigenicity, stemness, and therapy resistance. We then examine EMP’s role in the metastatic cascade, with particular focus on the E/M phenotype as the configuration most competent for systemic dissemination. Metabolic reprogramming is discussed as an integral dimension of EMP, covering dynamic shifts between glycolysis, oxidative phosphorylation, and lipid metabolism that fuel invasion and immune evasion. The TME is subsequently addressed as a critical extrinsic regulator of E/M states, through paracrine signals, biomechanical forces, and metabolic cues delivered by cancer-associated fibroblasts (CAFs), immune, endothelial, and neural cells. Experimental approaches, including dual-fluorescent reporters, organoids, genetically engineered mouse models, and spatial transcriptomics, are then surveyed alongside in silico methodologies. Finally, therapeutic strategies targeting EMP through signalling inhibition, epigenetic modulation, metabolic disruption, and TME reprogramming are critically discussed, emphasizing combination approaches and emerging targets for true EMP modulation.

### CSCs and EMP

CSCs, which were first identified in 1997 [1], constitute a minority but pivotal subpopulation within tumours that possess the unique ability to self-renew, differentiate into heterogeneous cancer cell types, and initiate tumour growth. Their discovery revolutionized the understanding of tumour biology by providing a hierarchical model, akin to normal tissue stem cells, in which CSCs drive tumour initiation, maintenance, progression, and metastasis [2]. CSCs often possess a dysregulated metabolic profile [3], and are endowed with resistance to conventional therapies such as chemotherapy and radiation [4], which preferentially kill the bulk of differentiated tumour cells but fail to eradicate the CSC fraction. This therapy resistance contributes to tumour relapse and poor patient prognosis. Plasticity is a hallmark of CSCs, referring to their ability to transition between stem-like and non-stem states, as well as among various intermediate states. This dynamic/plastic state enables tumours to adapt rapidly to changing TME conditions and therapeutic interventions, resulting in increased heterogeneity and resilience. The plastic nature of CSCs is influenced by intrinsic factors such as transcriptional regulators (OCT4, NANOG, SOX2, KLF4), epigenetic modifications, and non-coding RNAs, as well as extrinsic signals from the TME [5]. Indeed, TME plays a crucial role in driving the expansion or selection of specific CSC subsets during the different phases of tumour progression. The TME components, including CAFs, immune cells, extracellular matrix (ECM), hypoxia, and secreted cytokines, play essential roles in maintaining CSC phenotypes and plasticity by providing niche signals that modulate cellular states [6, 7]. For instance, hypoxia-inducible factors drive the expression of stemness and EMT-related genes in hypoxic tumour regions, promoting CSC maintenance and metastasis [8, 9]. The plasticity model merges the traditional hierarchical CSC model with the clonal evolution model by recognizing bidirectional transitions and cellular state interconversions as key drivers of intratumoral heterogeneity [2]. One major molecular program involved in CSC plasticity is the EMT and its reverse, MET. The EMT is a fundamental biological process essential for embryogenesis [10], wound healing, and tissue remodelling, and its partial and reversible activation in carcinoma cells has been linked to cancer progression and metastatic dissemination [11]. EMT is a dynamic and reversible biological process where epithelial cells can acquire mesenchymal features, thus adopting new characteristics regarding motility, immune evasion, and resistance to therapeutic agents [12]. Epithelial cells, from which EMT originates, are characterized by stable cell–cell junctions, apical–basal polarity, and interactions with the underlying basement membrane. In the context of EMT regulation, several key signalling pathways are activated at the plasma membrane and are influenced by lipid metabolism. Mutations in oncogenic pathways such as AMPK, PI3K, LKB1, RAS, WNT, TGF-Β, P53, and mTOR continuously alter cellular metabolism and drive the induction of EMT [13, 14]. Extensive research over the past decade has shifted this paradigm, revealing that EMT is better conceptualized as a continuum of states, rather than a strict epithelial versus mesenchymal binary status. To challenge this binary vision, since 2020, the term EMP has been preferred to encompass the diversity of the cellular states that can arise [15–17]. EMT endows epithelial tumour cells with mesenchymal properties, including motility and invasiveness, which are indispensable for metastasis. Importantly, EMT is tightly linked with the acquisition of stem-like traits, enabling disseminated tumour cells to initiate secondary tumours at distant sites [18]. However, full EMT is not always necessary or sufficient for stemness; rather, cells in E/M states often display the highest plasticity, tumorigenic potential, and resistance to therapies [19–21]. A recent study highlighted how cells can gain/retain mesenchymal properties, but not stemness, a mechanism regulated by the cell division process, particularly by epithelial splicing regulatory protein 1 (ESRP1). The overexpression of ESRP1 prevents the acquisition of stem-like traits, without impairing the mesenchymal phenotype. In this study, den Hollander and colleagues have shown that all cancer cells endowed with stemness exhibit mesenchymal traits, but not all mesenchymal cells are characterized by stemness [22]. Technological advances, such as single-cell RNA sequencing (scRNA-seq), have recently demonstrated that EMT does not operate as a binary process but rather spans a spectrum of intermediate or hybrid states, each characterized by distinct properties that fuel tumour heterogeneity, metastatic potential, and treatment resistance. Within this spectrum, E/M states coexist with fully epithelial and fully mesenchymal cells and display mixed marker profiles, endowing cancer cells with plasticity and diverse functional behaviours during tumour evolution and in response to therapy [23]. The relationship between CSC’s identity and EMP has been fundamentally revised by evidence demonstrating that stemness is not a property of fully mesenchymal cells but rather emerges preferentially within intermediate E/M states. As elegantly framed by Lambert and Weinberg, activation of an EMT programme generates a heterogeneous spectrum of quasi-mesenchymal phenotypes. It is specifically within these intermediate states, rather than at the fully mesenchymal pole, that cells acquire the highest tumour-initiating potential, a concept encapsulated in the notion of a tuneable “stemness window” along the EMT axis [24]. This association is mechanistically underpinned by the coupled regulatory circuitry of the miR-200/ZEB and LIN28/let-7 feedback loops, which collectively permit only E/M cells to achieve the intermediate levels of OCT4 and stemness gene expression that define a functional CSC state, as mathematically predicted by Jolly and colleagues and subsequently validated in multiple cancer models [25]. In this context, Canciello and co-authors further extended this framework by demonstrating that the E/M state represents a peak in both plasticity and stemness gene expression along the EMT continuum, with fully mesenchymal cells paradoxically losing the self-renewal and colonization capacity that defines metastatic competence [19]. To note, while the convergence of evidence linking E/M states to CSC properties has been compelling, it is important to acknowledge that these two cellular categories, despite sharing a remarkably overlapping molecular and functional signature, should not be regarded as fully coincident or interchangeable populations [26, 27]. Both E/M cells and CSCs could be inherently heterogeneous entities: the E/M compartment itself encompasses a spectrum of distinct intermediate phenotypes, differing in the relative balance of epithelial and mesenchymal marker expression, in their gene regulatory network configurations, and in their degrees of phenotypic stability, while the CSC compartment is equally heterogeneous, harbouring coexisting epithelial-like and mesenchymal-like stem cell subsets that differ in their proliferative activity, quiescence, and tumour-initiating capacity [28]. Demonstrating that these two populations are fully overlapping remains technically and conceptually challenging, as the markers currently used to isolate E/M cells and CSCs are not identical, are context- and tumour-type-dependent, and may capture distinct, although partially overlapping, subsets of cells within the same tumour [29]. Furthermore, the dynamic and reversible nature of both EMP and CSC state transitions means that the degree of overlap between these populations is likely to fluctuate over time and in response to microenvironmental cues, rendering any static characterization inherently incomplete [30]. Taken together, while E/M states and the CSC phenotype are deeply interrelated and likely mutually reinforcing, caution should be exercised in equating them, and future studies employing single-cell multi-omic approaches combined with functional lineage tracing will be essential to precisely delineate the boundaries and the true extent of the overlap between these two biologically distinct yet intertwined cellular identities.

Thus, a better understanding of the molecular and microenvironmental mechanisms governing cancer cell plasticity, particularly the interplay with EMT programs, holds promise for developing targeted therapies designed to overcome intratumoral heterogeneity and improve patient outcomes.

### EMP: from primary tumour to metastases

**Fig. 1:**
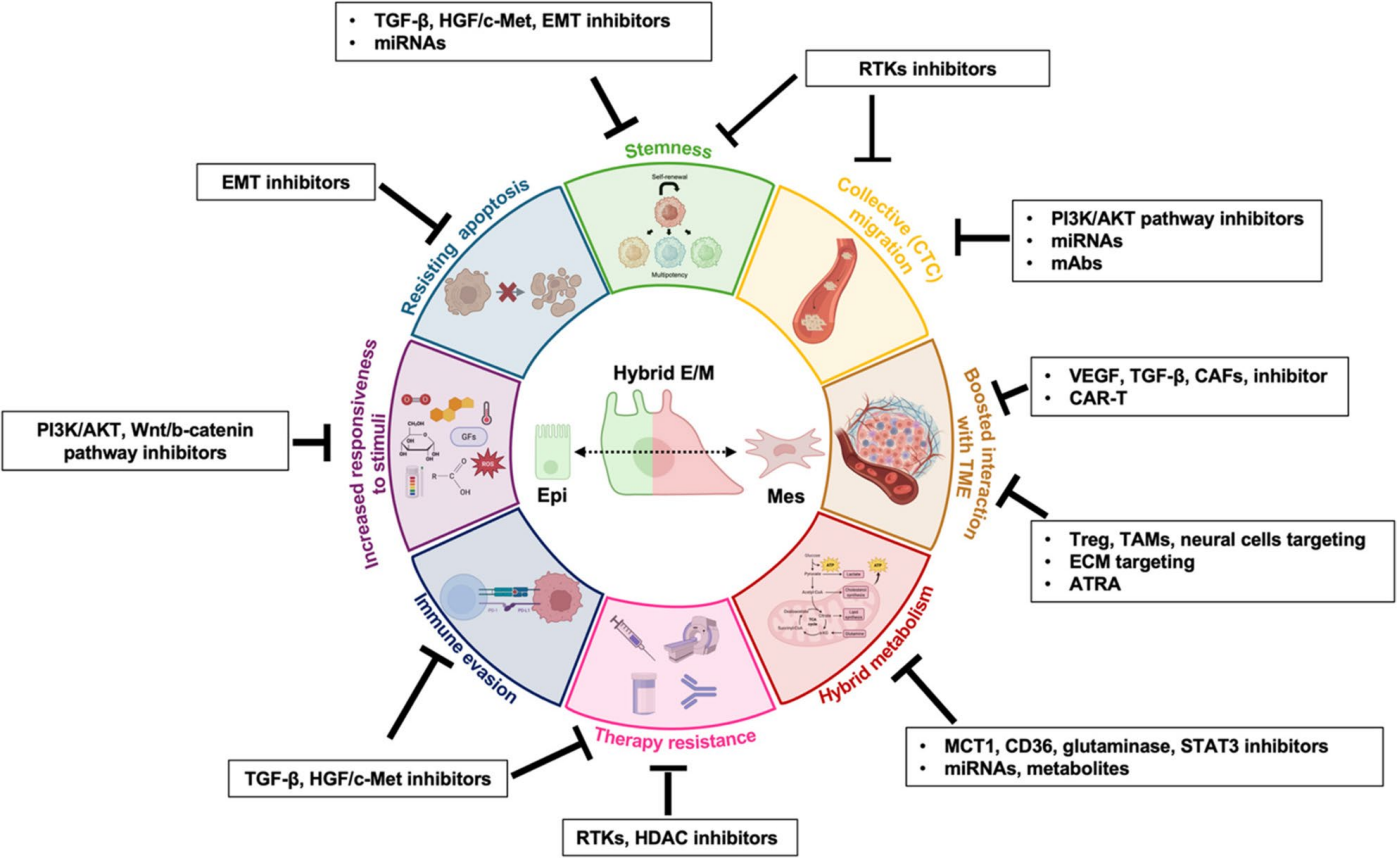
Hallmarks of EMP and target therapies. This figure represents a schematic diagram of the hallmarks of cancer cells endowed with EMP (coloured spokes and rounded boxes), and the novel therapeutic strategies to target them (black coloured squared boxes)

EMT is a pivotal biological process in tumour progression whereby cancer cells lose their epithelial characteristics, such as cell-cell adhesion and polarity, and acquire a mesenchymal phenotype, conferring enhanced migratory, invasive, and metastatic capabilities that drive disease advancement [31–33]. Besides its effect on tumour cells, EMT also affects TME thanks to the secretion of soluble factors, direct cell contact, release of exosomes and enzymes, as well as metabolic reprogramming [34, 35]. Between the two extremes lie E/M phenotypes, also called partial EMT states, in which cancer cells co-express epithelial and mesenchymal markers [25] (Fig. 1).

Cells in these hybrid states retain cell-cell junctions and some degree of epithelial morphology while acquiring mesenchymal traits such as motility and stress resistance. This plastic, intermediate phenotype confers several advantages during metastatic progression [36]. Notably, E/M cells demonstrate enhanced collective migration capabilities, allowing groups or clusters of tumour cells to invade and disseminate together while maintaining intercellular communication, which is critical for survival in the bloodstream and metastatic colonization [23, 31, 37]. Collective migration and cancer stemness represent two deeply intertwined facets of tumour progression, and recent research has begun to illuminate the molecular mechanisms that coordinate these processes at both the intracellular and intercellular levels [38]. Different studies shed light on distinct but converging regulatory axes that govern cancer cell plasticity, self-renewal, and invasive behaviour. Vilchez Mercedes and colleagues demonstrated that NRF2 acts as a phenotypic stability factor for E/M states during collective cancer migration. Using experimental and computational approaches, they showed that NRF2 prevents complete EMT and stabilizes an intermediate E/M phenotype immediately behind the leading edge, where it coordinates NOTCH-DLL4-JAG1 signalling to govern leader-follower cell asymmetry [39]. In this context, Lüönd et al. employed sophisticated dual recombinase lineage tracing in a breast cancer mouse model to demonstrate that E/M cells, marked by TENASCIN C expression, dynamically cycle between hybrid and epithelial states, acting as leader cells during collective invasion [40]. These cells were significantly enriched in lung metastases, and their selective ablation dramatically reduced metastatic burden, confirming their functional necessity. Full EMT cells, by contrast, remained stationary in perivascular niches and failed to colonize distant organs. Interestingly, both partial EMT (p-EMT) and full EMT populations contributed to chemotherapy resistance.

Indeed, these E/M states are implicated in tumour-initiating ability, immune evasion, and therapeutic resistance [41–46]. Unlike fully mesenchymal cells, which often show reduced proliferative capacity, E/M cells balance motility with growth potential, making them especially effective in establishing metastatic lesions. In different cancer models, circulating tumour cell (CTC) clusters displaying E/M markers have been shown to possess significantly higher metastatic capacity compared to solitary mesenchymal or epithelial CTCs [40, 47–49]. At the transcriptional level, E/M cells can be recognized by the concomitant expression of epithelial genes such as *CDH1*, *EPCAM*, or *CLAUDIN (CLDN)* family members together with mesenchymal genes including *VIM*, *ZEB1/2*, *SNAI1/2*, *TWIST1*, or *CDH2*-related programs, although the exact marker set is context-dependent and can vary by tumour type and assay platform. At the phenotypic level, these cells may display intermediate morphology, partial loss of cell-cell adhesion, retained epithelial organization, and acquisition of motility or invasive competence without full conversion to a mesenchymal phenotype. Functionally, E/M cells are often distinguished by a combination of collective migration, increased plasticity, stemness-associated traits, metastatic competence, and the capacity to dynamically revert toward either epithelial or mesenchymal states [50]. Several biomarkers have been proposed to identify hybrid EMT populations, but no single marker is sufficient on its own. Among transcript-based approaches, the VIM: CDH1 axis, and specifically the VIM: CDH1/CLDN7-associated framework, has been used to assign cells to epithelial, hybrid, or mesenchymal categories [51], while scRNA-seq can better resolve mixed marker expression within individual tumour cells. Protein-level markers that support a hybrid state include co-expression of epithelial markers such as EPCAM, E-CADHERIN, and cytokeratins with mesenchymal markers such as Vimentin, N-CADHERIN, and EMT-associated transcription factors, especially when these signals are detected in the same cell rather than in different cells within the sample. Additional support can come from functional readouts, including enhanced motility, invasion, spheroid/cluster dissemination, and stem-like behaviour, which are frequently associated with hybrid states. Importantly, an E/M state should be distinguished from a mixed population of separate epithelial and mesenchymal cells. In a true E/M state, both epithelial and mesenchymal markers are co-expressed in the same cell, whereas in a mixed population, the epithelial markers are confined to one subset and mesenchymal markers to another. This distinction requires single-cell or spatially resolved methods, such as scRNA-seq, multiplex immunofluorescence, imaging cytometry, or in situ hybridization, because bulk assays can incorrectly interpret admixture of distinct populations as hybridity. Recent advances have identified phenotypic stability factors, including GRHL2 and OVOL2, which modulate standard EMT regulators to maintain the E/M state [52–55]. However, it remains unclear if the hybrid EMT state constitutes a fixed cellular phenotype or merely captures a transient phase of fluctuating EMP in individual tumour cells.

To note, although EMT has long been considered a key driver of metastasis, accumulating evidence indicates that certain tumours can form distant lesions through EMT-independent routes. In these settings, cancer cells disseminate while largely maintaining epithelial features, using alternative mechanisms such as collective migration, passive shedding into circulation, or cooperation with stromal and immune components. Indeed, while EMP is widely implicated in promoting tumour dissemination, recent lineage-tracing studies in genetically engineered mouse models have challenged its necessity for metastatic colonization. In breast cancer models using mesenchymal-specific reporters (e.g., Fsp1-Cre or VIMENTIN-CreER driving a fluorescent switch from RFP to GFP upon EMT), lung macro metastases were predominantly composed of non-EMT-marked epithelial cells (RFP^+^), despite EMT events (GFP^+^) being detectable in 1–2% of primary tumour cells and enriched in circulating tumour cells (CTCs) [56, 57]. These findings indicate that EMP facilitates early steps such as invasion and chemoresistance but is dispensable for seeding and outgrowth of secondary tumours, as epithelial cells can directly form metastases [56, 58]. However, limitations of single-marker reporters, such as insensitivity to E/M or dynamic reversions, may underestimate EMP’s role, prompting calls for multi-marker, single-cell-resolved lineage-tracing [28, 59, 60]. Alternative mechanisms, including phenotypic cooperativity between EMT and non-EMT tumour subpopulations, heterotypic clustering, or stromal support, could enable epithelial cells to exploit mesenchymal advantages without undergoing full transition. Such findings challenge the traditional view that EMT is an obligate prerequisite for metastasis and underscore the remarkable plasticity of tumour cells in adapting to different microenvironmental and mechanical constraints.

The ability of cancer cells to reversibly transit via E/M states facilitates dissemination and seeding while permitting re-establishment of epithelial traits through MET, necessary for colonization and outgrowth of macro metastases [61]. The regulation of E/M involves complex signalling networks integrating diverse inputs from the TME and intracellular signalling pathways. EMT-inducing transcription factors (EMT-TFs) such as SNAIL, SLUG, TWIST, and Zinc finger E-box-binding homeobox (ZEB) families are extensively studied regulators of this plasticity [62]. These EMT-TFs can activate or repress epithelial and mesenchymal gene programs through direct binding to promoter regions or by recruiting epigenetic modifiers that remodel chromatin accessibility. EMT-TFs such as SNAIL and TWIST not only mediate phenotypic transitions but also contribute to the emergence of a subpopulation of cancer cells with self- renewal [63]. Similarly, expression of pluripotency factors like NANOG and OCT4 reinforces mesenchymal plasticity in oesophageal adenocarcinoma [64]. Overexpression of stemness markers such as NANOG, OCT4, and EMT-key regulator such as SNAI2 has been associated with poor prognosis across multiple tumour types, including ovarian, colorectal, oesophageal, and lung [65, 66]. A study by Mani et al. demonstrated a causal link between EMT and the CSC phenotype. They observed the emergence of cells expressing CSC markers and enhanced sphere-forming ability in mammary epithelial cells after EMT induction [63]. EMP is governed by a complex interplay of signalling pathways, including TGF-β/SMAD, WNT/ β-CATENIN, NOTCH, and HEDGEHOG, all of which are implicated in maintaining stemness and promoting EMT [67]. In colorectal cancer (CRC), disheveled3 (DVL3), involved in TGF-β1-induced EMT, was also shown to promote stemness and EMT-phenotype through the WNT/β-CATENIN/c-MYC/SOX2 axis [68]. A recent study performed on pancreatic ductal adenocarcinoma (PDAC) has shown that JAG1, one of the major cell surface ligands that activates the Notch signalling pathway, regulates the E/M state and identifies pancreatic CSCs [69]. From a preclinical perspective, the depletion of the E/M cell population led to a gradual collapse of organoids and inhibition of their tumorigenic capacity. Contrarily, the overexpression of JAG1 boosted the tumour-initiating cell self-renewal, confirming that JAG1-mediated regulation of the Notch signalling pathway controls the E/M cell phenotype in PDAC [69].

The EMP onset and CSC traits acquisition are orchestrated by multifactorial mechanisms, including epigenetic modifications and regulatory non-coding RNAs. These latter can modulate gene expression without altering the DNA sequence, thereby enabling dynamic and reversible changes in cellular phenotype [70]. A key example of epigenetic regulation is illustrated by DNA-methyltransferase 1 (DNTM1), an enzyme responsible for gene silencing through CpG island methylation. In prostate cancer cells, an increase of protein kinase C alpha (PKCα) and reduced DNA- methyltransferase 1 (DNMT1) expression by 5-azacitidine promotes the suppression of H3K9me3 and H3K27me3 on the promoter of EMT and stemness-related genes ZEB2 and KLF4, involved in somatic reprogramming. This therefore increases EMT, metastatic potential, emergence of CSC, and sphere formation in vitro [71]. PKCα is also reported to be involved in CSC-enriched populations of breast cancer [72]. Long non-coding RNAs (lncRNAs) play a pivotal role in modulating EMT and CSC programs by regulating transcriptional and post-transcriptional networks [73]. For instance, lncRNA MALAT1 and HOTAIR have been shown to promote EMT, stemness, and drug resistance in several cancer types through epigenetic remodelling and interaction with chromatin-modifying complexes [74–76]. The tumour microenvironment exerts a profound influence over EMP and CSC dynamics. In CRC, the 3D soft fibrin microenvironment may be responsible for the emergence of a hybrid phenotype resistant to ferroptosis via glutathione peroxidases/ferritin signalling. This resistant phenotype is thought to be the result of concomitant action of histone acetylation and the Wnt/β-catenin pathway [77]. It was also shown that microenvironment cues sustain expression of EMT and CSC markers and enhance mesenchymal potential of cells in a zebrafish xenograft model of human prostate cancer [78]. Similarly, the crosstalk between stromal and cancer cells can lead to both EMP and CSC phenotypes. For example, in the highly aggressive triple-negative breast cancer (TNBC), tumour-associated macrophages (TAMs) activate AKT signalling by CCL2 secretion, which increases β-catenin expression and leads to its nuclear localization, where it interacts with TCF/LEF transcription factors. This, therefore, promotes EMT and enriches the cancer cell population in CD24^−^/CD44^+^ and ALDH1 expressing cells [79]. In oesophageal adenocarcinoma, IL-6 secretion by fibroblasts favours cancer stem-like cells expansion by increasing mRNA and protein levels of CSC transcription factors like SOX2, OCT4, and NANOG, leading to EMP by NANOG [64]. Recent studies have demonstrated that cellular metabolism, mechanical stresses, and microenvironmental heterogeneity also orchestrate E/M states. For example, hypoxic niches induce HIF-1α, which cooperates with EMT-TFs to sustain partial EMT, while metabolic reprogramming towards glycolysis or oxidative phosphorylation supports cellular energy requirements during transition states [8, 80, 81]. Furthermore, extracellular matrix stiffness and cell-cell adhesion forces influence EMT progression by modulating intracellular signalling cascades and cytoskeletal reorganization. The E/M paradigm challenges traditional drug development strategies focused solely on eliminating purely epithelial or mesenchymal states. Instead, it highlights the importance of targeting intermediate phenotypes, which are arguably the most metastatic and therapy resistant. A better understanding of how E/M states arise, are maintained, and their crosstalk with CSC properties will guide novel interventions aimed at preventing dissemination, overcoming drug resistance, and curbing tumour recurrence. Increasing evidence suggests that EMP is a central driver of cancer stemness and tumour- initiating potential. In skin squamous cell carcinoma, deletion of the tumour suppressor FAT Atypical Cadherin 1 (FAT1) activates a dual signalling cascade. On one hand, phosphorylated Ca2+/calmodulin-dependent protein kinase II (CAMK2) activates the CD44-SRC pathway, promoting nuclear translocation of YAP, leading to ZEB1 expression, which induces a mesenchymal state. Simultaneously, FAT1 loss also inactivates EZH2, enabling SOX2 expression with the emergence of epithelial traits. The activation of these pathways results in a hybrid phenotype with enhanced tumour-initiating capacity and malignant progression [82]. EMP also contributes to disease progression and recurrence by enabling normal cancer cells to acquire CSC-like features. It has been demonstrated that stem-like cells arising from EMP resemble stem cells isolated from normal and neoplastic populations [63]. In TNBC, it was reported that intermediate E/M cells are enriched in CSCs [83]. The stable E/M phenotype driven by the SNAIL EMT-transcription factor and canonical Wnt signalling, identified by integrin β4 (CD104)^+^/CD44^high^ expression, a marker combination for CSCs, was also associated with enhanced tumorigenicity [84, 85]. EMP facilitates metastatic colonization through MET, which supports the implantation of CTCs in distant tissues [44]. In this scenario, recent studies demonstrated that E/M cancer cells act as leaders in collective invasion [86]. In PDAC, E/M favours CTC cluster formation and enables collective migration of cells, in contrast to the complete EMT program, associated with single-cell dissemination [38]. EMP may also promote intravasation of CTC and dissemination by activating angiogenesis-related genes, thereby facilitating escape from the primary tumour microenvironment [87]. Distinct EMT states play different roles in cancer progression [88]. Lineage tracing in a mouse model of breast cancer revealed that hybrid cells, but not fully mesenchymal cells, are required for lung metastasis. Although both partial and complete EMT cells contribute to chemoresistance, the latter contribute to a larger extent to it [40]. Further research by Youssef et al. helps distinguish two distinct EMP programs: one associated with invasion and the generation of hybrid stem-like cells, and another linked to inflammatory signalling in fully transitioned cells [89]. The dynamic nature of E/M states provides tumours with the flexibility to adapt and survive under therapeutic pressure. Future therapeutic strategies should aim to target the regulatory networks inducing both EMP and CSC phenotypes. Since EMP and CSC programs are often co-activated, targeting shared molecular nodes, such as VIMENTIN phosphorylation, could simultaneously suppress stemness and plasticity [90].

### Implications of E/M and metabolic reprogramming in cancer cells

**Fig. 2:**
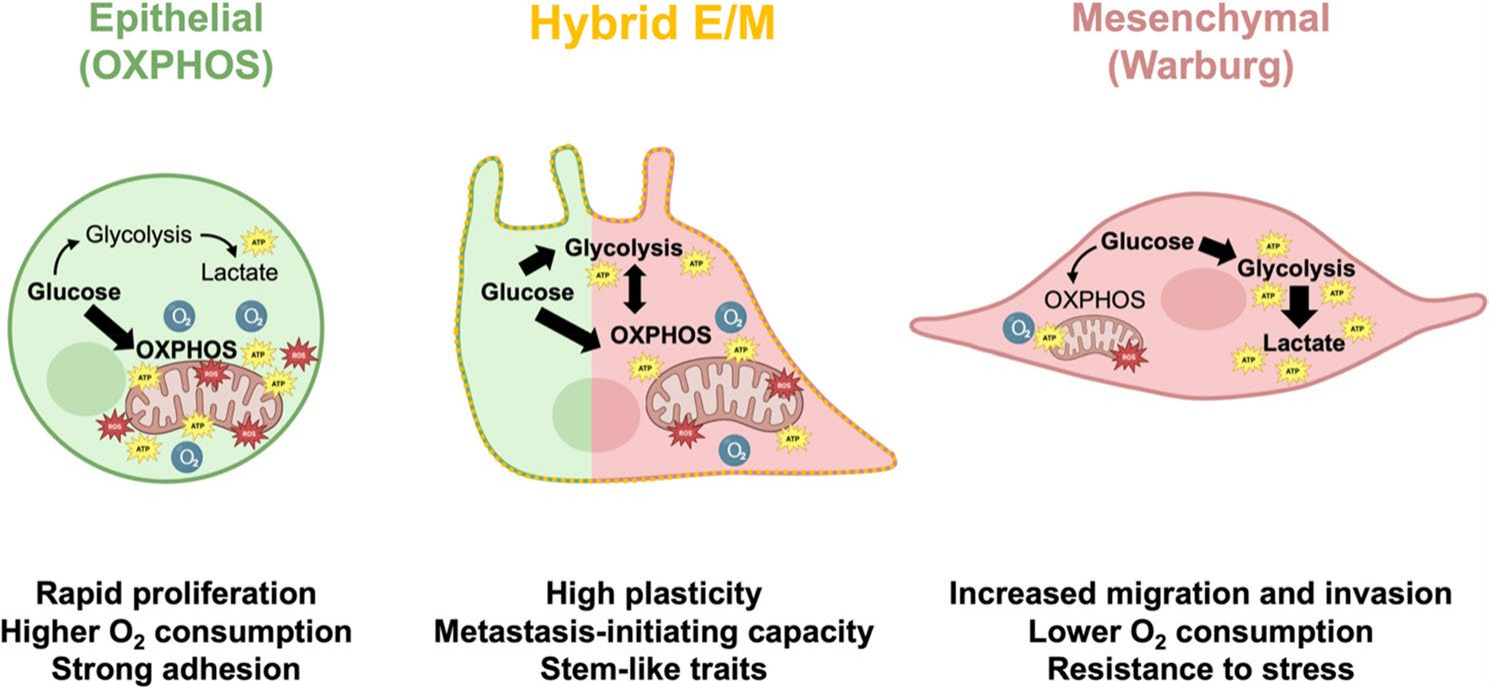
Metabolic rewiring associated with EMP. This figure shows the metabolic reprogramming of cancer cells from an epithelial phenotype (green colour), which mostly relies on OXPHOS metabolism, to a mesenchymal one (red colour) that is associated with enhanced glycolysis, passing through a hybrid phenotype that maintains both characteristics (yellow colour)

Metabolic reprogramming in neoplastic cells has been investigated since the identification of the Warburg effect in the 1920s [91]. Cancer cells exhibiting E/M possess the ability to switch between distinct metabolic states, such as glycolysis and oxidative phosphorylation (OXPHOS), depending on microenvironmental cues, nutrient availability, and energy demand. Metabolic reprogramming associated with E/M involves complex modifications in glucose metabolism, mitochondrial function, and lipid metabolism [92] (Fig. 2).

This metabolic adaptability confers upon CSCs the capacity to adapt to endogenous and exogenous microenvironmental stimuli, primarily through their own plasticity and considerable versatility in nutrient uptake and utilization [93]. Significant focus has been directed towards the metabolic perturbations intrinsically linked to pivotal stages of cancer dissemination, including but not limited to the initiation of metastasis, systemic circulation, subsequent colonization, and the development of chemoresistance [94, 95]. Here, although molecular mechanisms are not fully understood, we reported recent findings about the existing link between the metabolic reprogramming and the co-existence of the E/M phenotype in the CSC subpopulation.

### The role of glycolytic and OXPHOS metabolism in shaping the EMT plasticity spectrum

EMT drives cancer stemness through a spectrum of plastic cell states, and this phenotypic flexibility is fundamentally sustained by continuous shifts in metabolic flux, linking cellular identity transitions directly to metabolic reprogramming. Consequently, the interplay between EMT plasticity and the shifting metabolic landscape defines the adaptive potential of CSCs within the evolving TME. Interestingly, the acquisition of stemness during the EMT is driven by a sophisticated coupling of phenotypic plasticity and metabolic reprogramming [96]. While differentiated epithelial cancer cells primarily utilize OXPHOS [97, 98], the transition into an E/M CSC state necessitates a concomitant upregulation of glycolysis, resulting in a highly proliferative, bi-energetic phenotype (OXPHOS^high^/glycolysis^high^) [99–101]. These E/M cells may subsequently shift toward a mesenchymal-like state, characterized by metabolic quiescence and a reduction in OXPHOS and an increase in glycolysis (OXPHOS^low^/glycolysis^high^) [102, 103], or lose their stemness by downregulating glycolysis to differentiate into mesenchymal cancer cells (OXPHOS^high^) [103]. Accordingly, Ren et al. have discovered that stem-like and E/M breast cancer cells are able to drive tumour progression and metastasis formation via a metabolic reprogramming driven by the GPX2 /HIF1Α/P63 axis. In particular, within the primary tumour, the epithelial cells start acquiring an upregulation of mesenchymal genes, leading to the emergence of E/M subpopulations, supported by both OXPHOS and glycolysis. Later during tumour progression, disseminated cells in metastatic lesions regain epithelial traits, thus becoming mainly dependent on OXPHOS. This precise regulatory framework is governed by p63, which appears to orchestrate partial EMT or MET transitions in both primary and metastatic sites. Mechanistically, GPx2 knockdown initiates redox signalling by activating HIF1α, which regulates p63, leading to the onset of partial EMT and MET [104]. Similarly, a mouse breast cancer stem-like model, represented by 4T1 cells, exhibits an increased level of OXPHOS and glycolysis underlying the acquisition of a mixed metabolic and E/M phenotype (CK8^+^/αSMA^+^). Specifically, this metabolic signature appears to be sustained by PGC-1α, a transcription factor that drives the expression of metabolic signatures (including oxidative phosphorylation, lipid biogenesis, and mitochondrial biogenesis) and EMT-related genes, thereby driving the acquisition of an invasive phenotype [105]. In line with these findings, breast CSCs have been shown to comprise two EMT subpopulations. Firstly, the CD44^high^/CD24^low^, which are more-mesenchymal-like breast CSCs, and the aldehyde dehydrogenase (ALDH^+^) E/M cells. Interestingly, this latter is characterized by a mixed metabolic phenotype showing both OXPHOS and glycolysis, which drives metastatization [103, 106]. Moreover, another ALDH family member, ALDH1A3, drives the acquisition of E/M in CD44^+^/CD24^−^ TNBC stem cells. acc This finely regulated metabolic balance determines the acquisition of an E/M state in TN-BCSCs [107].

Finally, focusing only on glycolytic metabolism, Lactate dehydrogenase A (LDHA), which catalyses the conversion of pyruvate to lactate, the final step of anaerobic glycolysis, can promote EMT by upregulating the transcription factor ZEB2 [108]. Knockdown of LDHA has been shown to increase E-CADHERIN expression while decreasing the expression of focal adhesion kinase (FAK), matrix metalloproteinase 2 (MMP2), and vascular endothelial growth factor (VEGF), all of which are key regulators of EMT [109, 110]. Moreover, lactate produced by cancer cells contributes to EMT by lowering extracellular pH, which activates latent extracellular TGF-β [111]. Simultaneously, the efflux of lactate elevates intracellular pH, stimulating Wnt signalling and further promoting EMT [112]. NRF2, a key transcription factor of cellular metabolism, has been shown to induce and maintain an E/M phenotype. Specifically, NRF2 is positively regulated at the transcriptional level by AHR, ARNT, NF-κB, JUN, and MYC, while being negatively controlled at the post-translational level by CRLKeap1, SCFβ-TrCP, CRIF1, SIAH2, and RNF4 [113, 114]. From a metabolic perspective, emerging evidence suggests that NRF2 exerts a profound influence on cellular metabolism by enhancing fatty acid oxidation and repressing lipogenesis [115, 116]. The pivotal role of NRF2 in lipid metabolism is linked to the suppression of gluconeogenesis [117]. Taken together, these findings suggest that such a plastic and versatile phenotype cannot be confined to rigid metabolic categories. Instead, the acquisition of the EMT spectrum must be contextualized based on the specific cancer model, tumour stage, TME involvement, and driver mutations.

### Lipid metabolism and EMP

In this scenario of high heterogeneity and metabolic plasticity, lipid metabolism has recently been recognized as a key regulator of the EMT in cancer cells, and it is also linked to the balance between proliferative and invasive cellular states. Lipid metabolism has been recently defined as a main regulator of the EMT state of cancer cells, being also associated with different proliferative/invasive states and with the ability to adapt in highly nutrient and growth factor-deprived tumour microenvironments. Alterations in fatty acid synthesis and oxidation pathways help meet the increased energy demands and provide essential building blocks for cell membrane remodelling and signalling molecule production. Indeed, lipid metabolism is significantly altered in various cancers, during tumour initiation and progression. E/M cells have been shown to exhibit increased lipid uptake along with enhanced accumulation of lipid droplets, which serve as reservoirs to buffer oxidative stress, to supply energy during the metastatic cascade, and to resist therapy [13, 107, 118]. Dysregulated lipid metabolism can trigger EMT and stimulate multiple oncogenic pathways linked to this transition by modulating key signalling cascades, such as EGFR, RAS, MAPK, JAK2, STAT3, PI3K, AKT, and mTOR. Within EMT phenotypes, several critical signalling pathways are initiated at the plasma membrane and are influenced by lipid metabolic processes. Mammalian cells acquire lipids through two main mechanisms: de novo lipogenesis and lipid uptake. De novo lipogenesis is an intricate metabolic process whereby fatty acids are produced from non-lipid precursors, predominantly carbohydrates, leading to the production of long-chain fatty acids stored as triglycerides [13, 119, 120]. The enzyme primarily involved in this process is ATP citrate lyase (ACLY), which converts citrate into acetyl-CoA and plays a rate-limiting role in lipid synthesis. The produced acetyl-CoA is then carboxylated to form malonyl-CoA, marking the initial step in fatty acid biosynthesis. In addition, acetyl-CoA is a crucial substrate in the mevalonate pathway, where two molecules of acetyl-CoA combine to form acetoacetyl-CoA, ultimately leading to cholesterol production [121]. The phosphoinositide 3-kinase (PI3K)/AKT signalling pathway plays a central role in promoting cell survival across many types of cancer. In tumour cells, AKT directly regulates ACLY by inducing its phosphorylation and activation. Additionally, AKT enhances *ACLY* gene expression by activating the sterol regulatory element-binding protein 1 (SREBP-1), a transcription factor that controls genes involved in cholesterol and fatty acid biosynthesis. However, evidence suggests that the PI3K/AKT pathway primarily increases ACLY activity through post-translational modification, specifically phosphorylation, rather than through transcriptional upregulation. This phosphorylation also helps stabilize the ACLY protein. Beyond AKT, ACLY can be phosphorylated at multiple sites by other kinases, including nucleoside diphosphate kinase and cyclic AMP-dependent protein kinase [122]. ACLY serves as a central enzyme in de novo lipogenesis and acts as a metabolic link connecting lipogenesis with the Krebs cycle and gluconeogenesis [122]. Besides its specific role in regulating lipid metabolism, ACLY can act as a bridge, promoting EMT phenotypes through β-catenin signalling [92, 121]. Inhibition of ACLY suppresses tumour cell proliferation, growth, and stemness, with knockdown studies demonstrating reversal of EMT and inhibition of tumour growth via modulation of the PI3K/AKT pathway [123]. Acetyl-CoA carboxylase (ACC) catalyses the carboxylation of acetyl-CoA to malonyl-CoA, resulting in the synthesis of palmitate (16:0), a critical step in fatty acid metabolism [124, 125]. In different studies, ACC has been widely identified as a potential therapeutic target for suppressing tumour growth by modulating the metabolic alterations characteristic of cancer cells [126]. ACC is activated within the mitochondria in response to elevated citrate levels generated by citrate synthase. Two isoforms have been identified: ACC1, which is cytoplasmic, and its function is to synthesize malonyl-CoA used for *de novo* synthesis of fatty acids [127], and ACC2, which is associated with the mitochondrial membrane. ACC2 regulates fatty acid oxidation by catalysing the carboxylation of acetyl-CoA to malonyl-CoA, thereby inhibiting carnitine palmitoyl transferase I (CPT-I), the rate-limiting step in fatty acid uptake and oxidation [127]. These isoforms regulate de novo lipogenesis and fatty acid oxidation, respectively. Silencing ACC2 results in reduced β-oxidation and increased lipid accumulation, ultimately promoting the expression of genes associated with EMT, particularly an increase in TGF-β expression [127]. ACC1 regulates EMT through non-canonical pathways; in particular, TGF-β and leptin suppress ACC1 activity via AMPK-mediated phosphorylation at Ser79, thereby promoting EMT [128]. This effect is driven by the accumulation of acetyl-CoA following ACC1 inhibition, which enhances the acetylation of SMAD2, a key mediator of TGF-β-induced EMT [128]. In addition, ACC supplies fundamental substrates for de novo lipogenesis and functions in coordination with fatty acid synthase (FASN), converting acetyl-CoA into malonyl-CoA to support fatty acid chain elongation [124]. Fatty acid synthesis by FASN occurs in three main stages: (1) initiation, where acetyl-CoA and malonyl-CoA undergo condensation; (2) elongation, involving repeated cycles of reduction and dehydration that extend the fatty acid chain by two carbons each round; and (3) termination, in which palmitate is cleaved from the acyl carrier protein (ACP) [129]. In cancer cells, fatty acid synthase (FASN) expression is controlled by the transcription factor SREBP-1c [129]. Fatty acid synthase (FASN) catalyses the last step of de novo lipogenesis in different cancer types and is strongly associated with tumour progression [125, 129, 130] and is widely recognized as a driver of EMT [124, 129]. Evidence from patient-derived samples highlights a functional association between FASN, EMT, and major signalling pathways, including mTOR and EGFR. These pathways not only enhance lipogenic flux but also converge on transcriptional programs that promote EMT, including the upregulation of factors like SNAIL, TWIST, and ZEB family proteins. In this context, FASN-derived lipid products contribute to membrane remodelling and the formation of lipid rafts, thereby facilitating receptor-mediated signalling and amplifying downstream pro-EMT pathways [131]. In CSCs, FASN signalling plays a pivotal role in regulating the EMT phenotype. Its activity is tightly regulated at the post-transcriptional level, where specific microRNAs suppress FASN expression with a significant inhibition of EMT. Furthermore, TGF-β upregulates FASN expression, establishing a positive feedback loop in which enhanced lipogenesis promotes EMT [132, 133]. Stearoyl-CoA desaturase (SCD) introduces a double bond at the C9 position of long-chain saturated fatty acids, primarily converting stearoyl-CoA into oleoyl-CoA, a monounsaturated fatty acid, and is regulated by sterol regulatory element binding transcription factor 1c (SREBP1c) [134]. This modification markedly alters lipid properties and functions. Two isoforms of SCD are found in human tissues: SCD1 and SCD5. Both SCD1 and SCD5 isoforms are overexpressed and highly active in cancer cells, where they contribute to tumour progression. Increased SCD expression activates the Akt pathway, leading to phosphorylation and inhibition of GSK3β, which stabilizes β-catenin and enhances EMT signalling [135]. Stearoyl-CoA desaturase (SCD) is frequently co-expressed with EMT markers, where it downregulates the epithelial marker E-CADHERIN while upregulating the mesenchymal marker vimentin [136]. Acyl-CoA synthetases (ACSLs) are key enzymes in fatty acid metabolism that catalyse the conversion of long-chain fatty acids to acyl-CoA, a crucial step for phospholipid and triglyceride synthesis. Several isoforms, including ACSL1, ACSL3, and ACSL4, are frequently upregulated in cancer [92]. These isoforms display substrate specificity: ACSL1 preferentially activates oleate and linoleate, ACSL3 metabolizes myristate, palmitate, arachidonate, and eicosapentaenoic acid, whereas ACSL4 primarily utilizes arachidonate [137]. Notably, co-overexpression of ACSL and SCD has been associated with enhanced EMT regulation, marked by increased expression of SNAI2 and N-CADHERIN, as well as metastatic phenotypes. This synergistic effect likely arises from their combined roles in providing metabolic energy and promoting EMT-associated gene expression, thereby facilitating cancer cell invasiveness and progression [136, 138]. Lipolysis is a metabolic pathway that, under basal conditions, catalyses triacylglycerols into three fatty acids and one glycerol molecule through the action of specific enzymes: adipose triglyceride lipase (ATGL), hormone-sensitive lipase (HSL), and monoglyceride lipase (MGL). Inhibition of lipolysis enhances expression of EMT-associated genes and reduces endothelial cell differentiation capacity by suppressing E-CADHERIN transcription. In contrast, blocking glycolysis diminishes the expression of key EMT markers, including VIMENTIN, ZEB1, and SNAIL [139–141]. Furthermore, a strong association exists between EMT and fatty acid β-oxidation (FAO), largely driven by mitochondrial ROS signalling pathways, underscoring the importance of ROS in the biology of CSCs. FAO is regulated by transcriptional programs involving peroxisome proliferator-activated receptors (PPARs), particularly PPARα, which drive the expression of enzymes required for lipid catabolism. PI3K/AKT/ mTOR is a key regulatory pathway for FAO, and dysregulation of this pathway in tumour cells is closely associated with abnormal FAO metabolism [142]. Carnitine palmitoyl transferase 1 (CPT1) is the key rate-limiting enzyme of FAO, responsible for catalysing the transfer of long-chain acyl groups from coenzyme A to carnitine, enabling the transport of long-chain fatty acids into the mitochondrial matrix for oxidation [124]. Increased FAO has been suggested to sustain the self-renewal capacity of CSCs by controlling lipid and membrane biosynthesis, reducing oxidative stress through NADPH generation, and enhancing resistance to chemotherapy. CPT1A-driven FAO sustains cancer cell survival during detachment by maintaining intracellular NADPH levels and redox balance, thereby promoting anoikis resistance [143]. Conversely, a metabolic shift toward lipogenesis favours the generation of monounsaturated fatty acids, which are less prone to lipid peroxidation, thereby limiting ROS-induced damage in CSCs. Excess free fatty acids can also be converted into triglycerides and stored in lipid droplets to prevent lipotoxicity, an accumulation commonly observed in CSCs. Notably, this lipogenic shift further enhances glycolytic reprogramming, as fatty acid synthesis regenerates NAD+, which is essential for sustaining glycolysis [119]. Moreover, upstream mechanisms of lipid acquisition significantly contribute to the dependency on FAO observed in CSCs. Among these mechanisms, the fatty acid translocase CD36 has emerged as a key regulator of lipid uptake in both aggressive tumours and CSC populations. CD36-mediated fatty acid import fuels mitochondrial FAO, thereby meeting the heightened energetic and biosynthetic requirements characteristic of highly plastic cancer cells. Furthermore, CD36 signalling promotes EMT through activation of TGF-β pathways, characterized by reduced E-CADHERIN and increased vimentin expression [144, 145]. This contributes to the maintenance of stemness and enhanced metastatic potential, positioning CD36 at the crossroads of metabolic reprogramming and phenotypic plasticity. Upstream mechanisms of lipid acquisition significantly contribute to the dependency on FAO observed in CSCs. Notably, enhanced lipid uptake via CD36 has been demonstrated to support CSC survival and confer resistance to various stress conditions, including anchorage-independent growth. In mesenchymal-like CSC subsets, CD36-driven FAO is particularly crucial for survival under detachment conditions. By generating ATP and NADPH, FAO mitigates oxidative stress and facilitates prolonged persistence in the circulatory system, thereby promoting resistance to anoikis and enabling successful metastatic colonization [146]. The signalling networks that orchestrate EMT involve multiple growth factor pathways, such as mitogen-activated protein kinase (MAPK), Hedgehog, epidermal growth factor (EGF), NOTCH, and TGF-β, along with related downstream effectors like extracellular signal-regulated kinase (ERK), phosphatidylinositol 3-kinase (PI3K)/AKT, and nuclear factor kappa-B (NF-κB) [124]. These pathways are activated in response to metabolic stress, hypoxia, and inflammation, which collectively stimulate EMT-associated transcription factors, including ZEB1/2, SNAIL, and TWIST [32, 119]. In addition, EGF receptor (EGFR) contributes to the regulation of cancer cell metabolism by sustaining intracellular lipid and glucose homeostasis through mechanisms involving protein stability rather than kinase activity. When cellular energy levels are low, AMP and ADP binding activate AMPK, which stimulates FAO and promotes glucose uptake. Additionally, AMPK activates peroxisome proliferator-activated receptor gamma coactivator 1-alpha (PGC-1α), supporting oxidative metabolism and enhancing mitochondrial biogenesis [109, 147]. Notably, PGC-1α-driven metabolic reprogramming toward OXPHOS collaborates with EMT activation, further promoting cancer cell plasticity and progression [105]. Another example is represented by TGF-β, which is a potent inducer of EMT in various cancer types and is strongly associated with pro-oncogenic responses [148]. TGF-β signalling activates the TGF-β type I receptor (TGF-βRI), which phosphorylates the receptor-regulated Smad proteins, Smad2 and Smad3. Once phosphorylated, Smad2 and Smad3 form a complex with the common mediator Smad4 and translocate into the nucleus. At the nuclear level, they function as transcription factors, promoting the expression of SMAD2 and SMAD3 themselves and inducing genes associated with EMT (SNAIL and ZEB) and increasing the expression of mesenchymal markers [149]. Notably, blocking TGF-β signalling can reverse mesenchymal features and restore epithelial characteristics in cancer cells. Inhibition of FASN has been shown to disrupt EMT by downregulating mesenchymal markers, partly by interrupting the positive feedback loop between FASN and TGF-β1. Moreover, TGF-β-driven EMT is linked to increased ATP production and enhanced oxygen consumption, supporting the energy demands of transitioning cells. TGF-β1 has also been found to elevate ROS levels, which further drive cancer cell proliferation and EMT progression [150, 151].

While recent research has shed light on specific metabolic pathways implicated in this transition, the precise molecular mechanisms orchestrating these changes remain elusive. Further investigation is critically needed to unravel how alterations in metabolic pathways contribute to the plasticity and stability of the E/M state. Understanding these unresolved complexities is paramount for developing targeted therapeutic strategies. From a therapeutic perspective, targeting the metabolic vulnerabilities unique to E/M CSCs presents a promising avenue to disrupt metastasis and overcome drug resistance. However, the inherent metabolic plasticity of E/M cells poses challenges, as these cells can compensate for the inhibition of one pathway by shifting toward another metabolic route. Therefore, combination therapies and the development of biomarkers to predict and monitor metabolic states are critical for effective targeting. In conclusion, the interconnection between E/M and metabolic reprogramming endows cancer cells, especially CSCs, with phenotypic plasticity and metabolic flexibility that are essential for tumour progression, metastasis, and resistance to therapy. Investigating the molecular mechanisms underlying this coupling and developing strategies to disrupt these pathways may reveal novel and effective therapeutic modalities aimed at eradicating the most aggressive cancer cell populations.

### The bidirectional crosstalk between EMT and cancer metabolism

The molecular interplay between EMT and metabolic reprogramming remains a subject of intense debate and is not yet fully understood. Despite this uncertainty, emerging evidence suggests a bidirectional relationship: EMT can drive metabolic alterations and vice versa [152].

### EMT induces metabolic reprogramming

EMT involves extensive metabolic reconfiguration, driven by specific transcription factors, to meet the bioenergetic demands of enhanced motility and survival within adverse microenvironments. For example, SNAI1 expression was negatively associated with fructose-1,6-bisphosphatase 1 (FBP1), an enzyme involved in the glycolytic pathway [153]. In an NSCLC model, it was found that ZEB1 activates the expression of glucose transporter 3 (GLUT3) [154]. Similarly, overexpression of caveolin-1 (CAV1) in colorectal cancer models has been shown to enhance aerobic glycolysis by promoting HMGA1-induced GLUT3 transcription [155]. Furthermore, in NSCLC, TGF-β induces a metabolic shift from glycolysis toward OXPHOS. Specifically, TGF-β suppresses glycolytic flux while enhancing glutamate utilization by increasing carbon entry into the tricarboxylic acid (TCA) cycle. This metabolic reprogramming is mediated by the downregulation of pyruvate dehydrogenase kinase 4 (PDK4), an enzyme associated with EMT [156]. Similarly, in colorectal cancer, TGF-β drives EMT and promotes the nuclear translocation of pyruvate kinase M2 (PKM2), a key glycolytic enzyme responsible for converting phosphoenolpyruvate (PEP) to pyruvate [157]. In a glioblastoma model, TGF-β showed a crucial role in the upregulation of glucose transporter (GLUT) 1, hexokinase 2 (HK2), and lactate dehydrogenase A (LDHA) in glioblastoma [158]. It has been reported that TGF-β exhibits increased expression of 6-phosphofructo-2-kinase/fructose-2,6-biphosphatase 3 (PFKFB-3) in PANC-1 cells, which is linked to increased glucose flux within the cells and, consequently, lactate production [159]. Hexokinase, glyceraldehyde-3-phosphate dehydrogenase (GAPDH), and ENO, three glycolytic enzymes, were found positively enriched in both early and late EMT stages, upon TGF-β induction [160]. Analogously, ZEB1 enhances glucose uptake by upregulating the expression of glucose transporter 3 (GLUT3) [154]. Furthermore, during EMT, SNAIL plays a crucial role in the metabolic reprogramming from glucose flux between aerobic glycolysis and the pentose phosphate pathway (PPP) by repressing phosphofructokinase platelet (PFKP) [161]. Regarding lipid metabolism in prostate cancer, both TNF-α and TGF-β have been implicated in the increasing levels of unsaturated triacylglycerols [162]. Furthermore, SNAI1 overexpression in A549 NSCLC cells inhibits the transcriptional regulator of lipogenesis, carbohydrate-responsive element-binding protein (ChREBP), thereby downregulating both fatty acid synthase (FASN) and acetyl-CoA carboxylase (ACC) [151]. Taken together, these studies demonstrate that the EMT-driven metabolic landscape is highly context-dependent, varying between the promotion of glycolysis, the enhancement of oxidative process, and the modulation of lipid metabolism. By orchestrating the expression of key enzymes and transporters, the EMT program ensures that migrating cells can adapt their bioenergetic strategies to diverse microenvironments. Understanding this complex interplay could offer critical insights into identifying new therapeutic vulnerabilities in metastatic cancer.

### Metabolic reprogramming induces EMT changes

A growing body of evidence suggests that metabolic reprogramming plays a pivotal role in driving EMT-related changes. Recent findings indicate that mesenchymal cancer cells exhibit heightened metabolic requirements compared to their epithelial counterparts. This phenomenon may be attributed to the increased energy demands associated with the superior motility and invasive capacity typical of the mesenchymal phenotype. Consistently, Shaul et al. utilized public in silico datasets to demonstrate a robust correlation between the expression of 44 metabolic genes and of 1000 tumours characterized by high mesenchymal marker expression. Among these metabolic genes, they found Dihydropyridine dehydrogenase (DPYD), which encodes for an enzyme driving pyrimidine catabolism [163]. For example, from a lipid metabolism point of view, some lipid enzymes such as acetyl-CoA synthases (ACSL1 and ACSL4), SCD, support the EMT program in colorectal cancer [136]. Accordingly, hepatic cancer cells activate the EMT program, via Wnt and TGF-β, upon free fatty acids (FFAs) uptake driven by CD36 [144]. Emerging evidence links glycolytic metabolism and EMT alterations. The overexpression of the glycolytic enzyme phosphoglucose isomerase (PGI), which is implicated in the conversion of glucose-6P to fructose-6P, induces, via NF-κB, ZEB1 and ZEB2 activation and miR-200s repression [164]. In addition to glycolytic enzymes, fructose-bisphosphate aldolase A (ALDOA), an enzyme implicated in the conversion of fructose-1,6-bisphosphate to glyceraldehyde-3-phosphate and hydroxy-acetone, induces EMT via mesenchymal markers upregulation in lung squamous carcinoma [165]. Similarly, GAPDH supports EMT through SNAI1 expression in colon cancer [166]. The lactate dehydrogenase (LDHA/B), the enzyme that converts pyruvate into lactate during anaerobic glycolysis, showed a positive correlation with EMT markers in muscle-invasive bladder cancer [167]. Some evidence showed that high glucose plays a crucial role in cancer. For example, glucose drives, in CRC, a reduction of E-CADHERIN and upregulation of VIMENTIN [168]. In addition, high glucose levels promote EMT by increasing YAP1 and TAZ in breast and bladder cancer models [169, 170]. Metabolic mitochondrial dysfunctions are also linked to EMT reprogramming in several cancer histotypes. Fumarate hydratase (FH) is a mitochondrial enzyme that is involved in fumarate to malate conversion. In renal cancer, FH induces EMT signature via miR-200b/200a/429 [171, 172]. Conversely, lymphoid-specific helicase (LSH), after recruiting the epigenetic silencer factor G9a, represses FH. Downregulation of FH level leads to an abrogation of succinate, fumarate, and malate production, which in turn downregulates E-CADHERIN and ZO-1 expression and overexpresses VIMENTIN in nasopharyngeal carcinoma [173, 174]. Succinate dehydrogenase (SDH) converts succinate to fumarate. SDH downregulation promotes cell migration and invasion via TGF-β/SNAI1 in colon and ovarian cancer [175, 176]. Furthermore, Succinate Dehydrogenase Complex Assembly Factor 2 (SDHAF2) via glycogen-synthase kinase (GSK-3β)-β-catenin axis induces EMT in lung cancer cells [177]. Isocitrate dehydrogenases (IDHs) are enzymes that catalyse the conversion of isocitrate to alpha-ketoglutarate (α-KG). Mutations in IDH genes are involved in the activation of the EMT pathway via the miR200-ZEB1 axis [178]. Amino acids represent an alternative metabolic source useful for cancer metabolism [179]. It has been shown that the distal-less homeobox-2 (Dlx-2)/glutaminase 1 (GLS1)/glutamine (Gln) metabolism axis drives TGF-β/Wnt/SNAIL-induced EMT and the glycolytic switch. Mechanistically, DLX-2 enhances SNAIL mRNA stability by inducing GLS1/Gln metabolism [180]. Glutaminase 2 (GLS2) has a contradictory role in inducing or suppressing the EMT process [181, 182]. However, it has been demonstrated that GLS2 induces EMT by increasing ERK phosphorylation and, consequently, both ZEB1 and VIMENTIN levels [182]. Similarly, another enzyme involved in the amino acid metabolism/EMT duality is glutamate dehydrogenase (GDH), which is implicated in α-KG production from glutamate. GDH upregulation drives the tyrosine phosphorylation of STAT3, mediating EMT acquisition in CRC models [183]. Collectively, this evidence highlights the existence of a regulatory axis from cellular metabolism to EMP. The modulation of these metabolic pathways underscores how intermediate metabolites act as signalling molecules to determine the fate of specific EMT states. Deciphering these complex interactions provides a framework for identifying metabolic vulnerabilities that could be exploited in anti-cancer therapies.

Disentangling the causal directionality of the EMT–metabolism axis requires orthogonal deconvolution strategies that collectively resolve both the molecular hierarchy and the spatiotemporal dynamics of this crosstalk. First, temporal single-cell multi-omics, integrating scRNA-seq with single-cell metabolic gene signatures and pseudo time trajectory inference, could enable the mapping of EMP states onto discrete metabolic programs, identifying whether glycolytic, oxidative, or lipid-anabolic shifts precede or follow transcriptional EMT commitment; this approach is particularly powerful for resolving hybrid E/M intermediates, which bulk transcriptomics systematically obscures. Second, stable isotope tracing with ¹³C-labeled glucose or glutamine in isogenic CRISPR-edited models, where individual EMT-TFs are selectively knocked out or activated, would allow direct quantification of carbon flux through glycolysis, the pentose phosphate pathway, and the TCA cycle under defined genetic perturbations, causally assigning each metabolic rewiring to a specific transcriptional driver rather than inferring it from correlative data. Third, in vivo genetic lineage tracing, combining Cre-lox systems driven by EMT-specific promoters with fluorescent metabolic biosensors, would enable dynamic tracking of metabolic state transitions within cells undergoing EMT in living tumour models, capturing the spatially resolved heterogeneity between invasive fronts and tumour cores that static in vitro assays cannot recapitulate. Each of these strategies, discussed in detail in the following sections, addresses a distinct layer of the EMT–metabolism relationship and, when applied in concert, offers a framework to determine whether metabolic reprogramming is a driver, an enabler, or a consequence of specific EMP states, with direct implications for identifying context-dependent therapeutic vulnerabilities.

### Tumour microenvironment and EMP

EMP in cancer cells is dynamically regulated by the multifaceted TME, encompassing cellular components, CAFs, immune cells (i.e., tumour-associated macrophages TAMs, myeloid-derived suppressor cells (MDSCs), T cells, endothelial cells (ECs), pericytes, and non-cellular elements like the ECM, all of which deliver paracrine signals, biomechanical forces, and metabolic cues to drive E/M states promoting invasion, stemness, metastasis, and therapy resistance [184–186].

### CAFs, endothelial, and immune cells

CAFs, the predominant stromal cells originating from resident fibroblasts, epithelial/endothelial-mesenchymal transitions, or bone marrow precursors, secrete TGF-β, IL-6, PDGF, and HGF to activate SMAD2/3, STAT3, and PI3K/AKT pathways, upregulating EMT transcription factors (SNAIL, SLUG, TWIST, ZEB1/2) while repressing E-CADHERIN [187, 188]. Endothelial cells contribute via VEGF/Notch signalling, inducing endothelial-mesenchymal transition-like responses in tumour cells [189], and secreting TGF-β/Wnt ligands that stabilize β-catenin and NICD to reinforce partial EMP [190, 191], facilitating vascular mimicry and intravasation [192, 193]. Pericytes stabilize aberrant vessels but, when activated, release PDGF-BB to crosstalk with CAFs [194, 195], amplifying ECM remodelling and stiffness [196]. Immune cells profoundly shape EMP: TAMs (M2-polarized) produce TGF-β, TNF-α, IL-6/10, and CCL2 to trigger STAT3/SMAD-mediated Snail/ZEB expression and immune evasion via PD-L1 upregulation in mesenchymal states [197–199]; MDSCs suppress anti-tumour immunity while secreting ARG1/IL-6 to sustain plasticity [200, 201]; regulatory T cells contribute TGF-β/ID1 signals for hybrid phenotypes [202–204]. On the other hand, E/M cancer cells can be crucial during the metastatic process, thanks to their crosstalk with immune cells, as highlighted by their capacity to express high levels of inhibitory signals that protect them from NK cell-mediated clearance [45], or to express high levels of PD-L1, thus generating an immune-evasive phenotype, without requiring the need to undergo a transition toward a full mesenchymal phenotype [46]. These interactions create immunosuppressive loops, with mesenchymal cells recruiting more protumour immune infiltrates [205].

### Neural cells

Neural cells within the TME, including neurons, Schwann cells, and neural crest-derived glia, actively contribute to cancer progression by releasing neurotrophic factors such as brain-derived neurotrophic factor (BDNF), nerve growth factor (NGF), and neurturin (NRTN), thereby fostering tumour-neural crosstalk that drives malignancy. These peritumoral nerves innervate tumours and promote plasticity through synaptic-like connections, where neurotransmitter release and neurotrophins signalling reprogram cancer cells toward stemness and invasiveness [206, 207]. Neurotrophic factors play a pivotal role in cancer biology by modulating tumour cell plasticity, particularly through the regulation of EMT [206]. These factors bind to high-affinity Tropomyosin receptor kinase (Trk) receptors (e.g., TRKA for NGF, TRKB for BDNF) and the low-affinity p75NTR receptor, activating downstream pathways such as PI3K/AKT, MAPK/ERK, and PLCγ, which converge to reprogram cellular states [208]. In CSCs, BDNF/TrkB signalling upregulates EMT transcription factors like SLUG, SNAIL, and TWIST1, driving the acquisition of mesenchymal traits, such as increased N-CADHERIN, VIMENTIN, and ZEB1 expression, while suppressing epithelial markers like E-CADHERIN [206, 209, 210]. This shift not only enriches CSC pools with self-renewal capacity (e.g., via sphere formation and CD44^high^/CD24^low^ phenotypes in breast and lung cancers) but also confers resistance to therapies by enabling adaptive plasticity in response to stress. In specific contexts, NGF induces SLUG via p75NTR in breast cancer cells, promoting invasion and stemness, while NRTN enhances ZEB1/N-CADHERIN in colorectal and pancreatic tumours to boost motility and angiogenesis [211]. BDNF further supports glioma progression through synaptic plasticity mechanisms, including AMPA receptor trafficking and CAMK2 activation, linking neural-tumour interactions to metastatic potential [207]. Collectively, these neurotrophin-driven processes underscore their contribution to tumour heterogeneity, progression, therapeutic evasion, and plasticity, positioning their targeting as promising agents to disrupt plasticity.

### ECM

The ECM, comprising collagens (I/IV), fibronectin, hyaluronic acid, and laminins, undergoes CAF/MMP-driven remodelling, increasing stiffness that engages integrin-α5β1/αvβ3-FAK/Src/RhoA/YAP/TAZ mechanosignalling to lock EMP, disrupt basement membranes, and promote invadopodia formation [212–215]. Moreover, ECM proteins like fibronectin cooperate with TGF-β for ERK/MAPK activation [216–218]. However, recent studies have highlighted that matrix stiffness can regulate invadopodia in a context-dependent manner. In 3D basement membrane-like matrices, Chang et al. showed that increasing stiffness reduced the probability of invadopodia extension, shortened invadopodia length and lifetime, and impaired cell migration, indicating that a stiffer and less permissive environment can physically restrict protrusive invasion. Importantly, these findings contrast with earlier 2D gelatine- or collagen-based assays, in which increased stiffness was associated with greater invadopodia-mediated degradation, suggesting that stiffness does not exert a uniform effect across experimental systems. This apparent discrepancy likely reflects fundamental differences in dimensionality, matrix composition, and readout. In 2D assays, invadopodia are scored mainly by proteolytic degradation on rigid, ligand-coated substrates, whereas in 3D basement membrane-like matrices, invadopodia must extend through a confined, mechanically plastic environment and support directional migration. Thus, stiffness may enhance degradative activity under planar conditions while simultaneously limiting protrusion extension in 3D by increasing confinement and reducing matrix plasticity. Taken together, the available evidence suggests that invadopodia respond not only to stiffness per se, but also to the mechanical architecture of the surrounding matrix, interpreting stiffness-dependent effects as highly context dependent [219]. Collectively, these TME elements act in synergy, with CAF-ECM stiffening that amplifies EC/TAM-derived TGF-β/Notch signalling, yielding reversible hybrid states resistant to therapies. Thus, the targeting of multi-axis using specific inhibitors (TGF-β traps, anti-FAK, CAF-depleters) could disrupt EMP networks.

#### Models to monitor the E/M state of cancer cells

#### In vitro models

Recent advances in in vitro modelling have dramatically expanded our capacity to monitor and dissect EMP in cancer cells at unprecedented molecular and temporal resolution (Table 1).

**Table 1:** Summary table showing in vitro and in vivo models to investigate the EMT status of cancer cells

**Approach**	**Applications**	**Representative ** **Studies/Tools (PMID)**	**Strenghts**	**Limitations**
Network inference & GRN modeling	Identify EMT drivers, reconstruct regulatory circuits	SpidermiR [173], Network analysis [171]	Reveals key regulators, integrates multi-omics	Dependent on data quality, may miss context-specific effects
Single-cell & spatial transcriptomics	Map EMT/hybrid states, analyze heterogeneity	Spatial transcriptomics [34, 168], scRNA-seq [179]	High resolution, spatial context, captures plasticity	Costly, complex analysis, limited by tissue sampling
Machine learning & deep learning	Predict EMT states, drug response, biomarker discovery	UNAGI [169], ML review [170], ML orgenelles [175], ML morphology [176].	Handles high-dimensional data, enables *in silico*screening	Requires large datasets, interpretability challenges
Agent-based & dynamic modeling	Simulate EMT transitions, study plasticity & evolution	Agent-based [177], >ODE/Boolean [178]	Captures dynamics, models cell-cell interactions	Model assumptions, computationally intensive
Multi-omics integration	Uncover novel signatures, link molecular layers	SpidermiR [171],UNAGI [169], ML review [170]	Holistic view, identifies crosstalk between pathways	Data integration challenges, batch effects

Dual-fluorescent reporter systems, which utilize promoter regions of epithelial (e.g., E-CADHERIN) and mesenchymal (e.g., VIMENTIN) markers to drive spectrally distinct fluorophores (such as RFP and GFP), have emerged as powerful tools to track EMT dynamics in real time across colorectal, lung, and breast cancer models [220]. These dual-reporter constructs enable the identification and isolation of distinct epithelial (E), mesenchymal (M), and E/M subpopulations via flow cytometry and live-cell imaging, revealing that intermediate phenotypes represent a balanced stemness and invasive capacity, compared to pure epithelial or mesenchymal states, thus making it the ideal target for designing novel antitumour therapies. Additional reporter strategies incorporate microRNA-based sensors, such as miR-200 fused to fluorescent proteins, to robustly detect EMT and MET events, thereby capturing bidirectional plasticity and distinguishing cancer stem cell-like populations within heterogeneous tumours [221]. Complementing these plasmid-based systems, CRISPR/Cas9-mediated endogenous tagging of EMT-related genes (*SNAI1*,* ZEB1*,* CDH2*) with fluorescent proteins provides physiologically relevant models that preserve native regulatory elements, subcellular localization, and post-translational modifications, thus avoiding overexpression artifacts [222, 223]. CRISPR-based knockout/knockdown and overexpression approaches dysregulating phenotypic stability factors (e.g., ESRP1, GRHL2, and OVOL2) further permit dissection of the molecular circuitry governing E/M state stabilization and reveal how loss of these factors drives cells toward complete EMT [224–227]. In parallel, sophisticated three-dimensional (3D) culture platforms, including patient-derived organoids (PDOs), tumour spheroids embedded in ECM, and microfluidic tumour-on-chip devices, recapitulate critical TME features such as tissue architecture, hypoxia, matrix stiffness, and biochemical gradients [228–232]. TGF-β-induced EMT in breast cancer PDOs, for instance, triggers morphological changes, downregulation of E-CADHERIN, cytoskeletal reorganization, and enhanced organoid adhesion to culture substrates, reflecting migration and invasion through the ECM [233]. Advanced imaging modalities, combined with deep learning algorithms, enable the quantitative, high-throughput assessment of EMT susceptibility, inter- and intra-organoid heterogeneity, and dynamic cellular responses across patient-derived lines. Microfluidic platforms further enhance these capabilities by providing spatiotemporal control over nutrient delivery, drug exposure, and mechanical cues, while permitting real-time visualization of collective cell invasion, single-cell EMT dynamics, and phenotypic switching under defined conditions [234, 235].

#### In vivo models

**Fig. 3:**
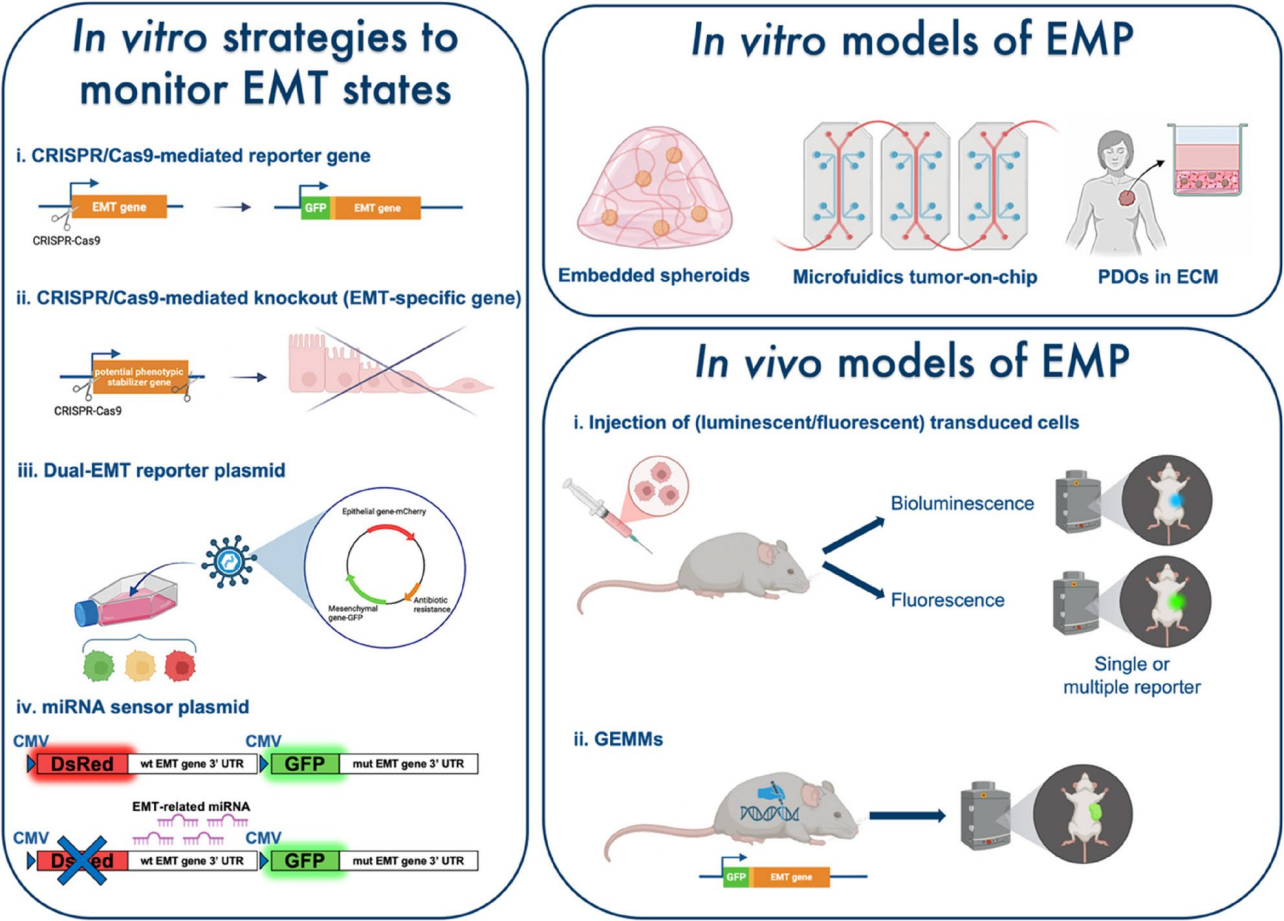
In vitro and in vivo models to study EMP. Schematic overview of in vitro and in vivo strategies for monitoring EMT states and studying EMP in preclinical models

In vivo monitoring of EMP in mouse models has been revolutionized by bioluminescence imaging (BLI) and fluorescent protein-based reporter systems, enabling real-time, non-invasive tracking of EMT dynamics during tumour progression and metastasis. Luciferase-based reporters provide the most sensitive and quantitative approach for longitudinal in vivo tracking, as bioluminescent signals from firefly or Renilla luciferase-expressing cancer cells can be detected throughout the body, even in deep tissues, with photon emission correlating linearly with tumour burden. Dual luciferase reporter systems that combine constitutive expression of one luciferase isoform (e.g., firefly luciferase for tumour burden), with EMT-responsive expression of a second isoform (e.g., Renilla luciferase driven by ZEB1 or SNAI1 promoters) enable ratiometric quantification of EMT status in xenograft and orthotopic models [236]. For instance, Renilla luciferase fused to the 3′-UTR of ZEB1 mRNA serves as a biosensor for miR-200 family activity, with bioluminescence signals being strongly repressed in epithelial cells but activated in mesenchymal or TGF-β-treated cells undergoing EMT. Orthotopic xenograft models implanted with luciferase-tagged cancer cells into anatomically relevant sites (e.g., colon, pancreas, lung) recapitulate the natural tumour microenvironment and metastatic dissemination patterns observed in patients, with serial BLI measurements enabling detection of primary tumour growth and distant metastases without sacrificing animals. In colorectal cancer models, subcutaneous or orthotopic injection of CT-26-Luc cells results in lung metastases detectable by BLI within 1–2 weeks, with bioluminescence intensity showing strong correlation with tumour volume measured by micro-computed tomography (micro-CT) imaging. Genetically engineered mouse models (GEMMs) carrying tissue-specific, inducible EMT reporters represent the most physiologically relevant systems for studying spontaneous EMT during *de novo* tumorigenesis. Dual reporter GEMMs of PDAC engineered with fluorescent proteins driven by EMT markers, such as α-smooth muscle actin (αSMA) and fibroblast-specific protein 1 (FSP1), enable lineage tracing of EMP in spontaneously arising tumours within an intact immune microenvironment [237]. These GEMM-based systems revealed that EMT programs are dynamically regulated during PDAC progression, with αSMA-positive mesenchymal cells exhibiting enhanced invasiveness and contributing to tumour heterogeneity. Intravital microscopy (IVM) combined with fluorescent protein reporters provides the highest spatiotemporal resolution for visualizing EMT dynamics in living mice. Multi-photon IVM through surgically implanted imaging windows (e.g., mammary, cranial, dorsal skin, or lung windows) allows repeated imaging of the same tumour region over days to weeks, revealing that motile, invasive tumour cells express mesenchymal markers (e.g., VIMENTIN-GFP) while proliferative cells at the tumour core retain epithelial characteristics (E-CADHERIN-RFP) [238, 239]. Recent IVM studies using CSC biosensors, such as the SORE6 > GFP reporter activated by SOX2/OCT4 binding, demonstrate that CSCs exhibiting EMT-like properties are enriched at tumour-microenvironment of metastasis doorways, specialized microanatomical sites where cancer cells interact with perivascular macrophages to facilitate intravasation [240, 241]. Furthermore, IVM with FRET-based biosensors for SRC, Rho GTPases, or caspase-3 activity enables real-time visualization of signalling events driving EMT, migration, and resistance to apoptosis at single-cell resolution within the native TME [242–244]. Collectively, luciferase-based BLI for whole-body metastasis tracking, fluorescent protein reporters in GEMMs for lineage tracing, and IVM for subcellular dynamics constitute a comprehensive in vivo toolkit for dissecting EMP across multiple scales, from molecular signalling to tissue-level tumour architecture, during cancer progression and therapeutic response. Collectively, these integrated in vitro and in vivo models (Fig. 3), spanning genetically encoded reporters, CRISPR-engineered cell lines, 3D organoid systems, microfluidic devices, and GEMMs, constitute robust and versatile toolkits for interrogating the molecular mechanisms, regulatory networks, and therapeutic vulnerabilities underlying epithelial-mesenchymal plasticity in cancer.

In conclusion, single- and dual-reporter systems offer distinct advantages and caveats in studying EMP. Single reporters, such as luciferase- or fluorescence-based constructs driven by EMT-associated promoters, provide sensitive, quantitative, and often non-invasive tracking of EMT dynamics in vitro and in vivo, yet they capture only unidimensional aspects of phenotypic transitions. In contrast, dual-reporter systems linking epithelial and mesenchymal promoters to spectrally distinct fluorophores enable simultaneous visualization of bidirectional transitions, intermediate hybrid states, and cell-state heterogeneity, thus offering higher spatiotemporal resolution and functional insight into dynamic EMP trajectories. However, these approaches may suffer from promoter leakiness, artificial overexpression, or limited physiological relevance, particularly in plasmid-based models that lack endogenous regulatory context. CRISPR/Cas9-mediated knock-in reporters and GEMMs partially overcome these limitations by preserving native gene regulation and allowing lineage tracing within the tumour microenvironment. Overall, integrating single and dual reporters across complementary platforms, from 3D organoids to intravital microscopy, provides a more comprehensive and mechanistically faithful framework to dissect EMP regulation during cancer progression and therapeutic response.

#### In silico methodology to study the EMT process

The identification and quantification of E/M states remain challenging due to the lack of reliable biomarkers and the inherent fluidity of the process. However, scRNA-seq and spatial transcriptomics have enabled the mapping of these populations within tumours, revealing that E/M cells are often enriched at the invasive front and are associated with increased metastatic potential and therapy resistance [23, 44, 245]. In this context, in silico methodologies have emerged as indispensable tools, enabling the integration and analysis of high-dimensional omics data, the modelling of complex regulatory networks, and the simulation of dynamic cellular processes [246–248]. For example, spatial transcriptomics has been used to localize E/M populations within the tumour microenvironment, demonstrating their spatial association with invasive niches and their dynamic response to microenvironmental cues [245]. GEMMs employing lineage tracing strategies, such as vimentin- and E-CADHERIN-driven GFP and RFP reporters [220], have provided direct evidence that hybrid and mesenchymal lineages are crucial for tumour progression and metastasis. Ablation of EMT-proficient cells in these models dramatically impairs both primary tumour growth and metastatic dissemination, while spatial profiling and whole-genome sequencing have revealed that EMT-derived cells are associated with increased chromosomal instability and chromothripsis, directly linking EMP to tumour evolution and the emergence of high-fitness malignant clones [249, 250]. In this scenario, network-based approaches have been instrumental in dissecting the regulatory logic underlying EMT and its hybrid states. Tools such as SpidermiR [251] enable the integration of gene expression, copy number variation, and miRNA data to reconstruct gene regulatory networks (GRNs) in cancer. In prostate cancer, SpidermiR was used to identify miR-145-5p as a central regulator of an aggressive gene network, highlighting how network inference can pinpoint non-canonical drivers of EMT and hybrid phenotypes. Similarly, network topology analysis has been applied to identify master regulators of EMT in various tumour types, revealing that E/M states are often governed by complex, multi-layered regulatory circuits [248].

Single-cell RNA-seq and spatial transcriptomics have revolutionized the study of EMT dynamics by enabling the high-resolution mapping of cellular states within tumours [252]. These technologies have been pivotal in identifying and characterizing E/M populations. For instance, spatial transcriptomics was used to map E/M cells at the invasive front of tumours, demonstrating their spatial heterogeneity and association with metastatic potential [245]. Single-cell analysis has further revealed that E/M states are not rare intermediates but constitute a significant fraction of tumour cell populations, with distinct transcriptional programs and functional properties [23, 44]. A landmark study using scRNA-seq in head and neck squamous cell carcinoma identified a specific E/M signature that was spatially localized at the leading edge of primary tumours; notably, this E/M program was found to be a better predictor of nodal metastasis than canonical EMT markers, highlighting the clinical relevance of hybrid states [253–255]. These approaches have also uncovered the dynamic plasticity of E/M cells, including their ability to revert to epithelial or progress to fully mesenchymal states in response to environmental cues. In parallel with single-cell and spatial approaches, several transcriptomics-based scoring metrics have been developed to quantitatively position patient samples along the epithelial–hybrid–mesenchymal (E–H–M) spectrum. A seminal study introduced an E/M gene expression scoring metric capable of stratifying tumours based on their hybrid phenotype and predicting survival outcomes across multiple cancer types, demonstrating that E/M states are often associated with poor prognosis and increased metastatic potential [51]. Subsequent comparative analyses systematically evaluated different EMT scoring frameworks, highlighting their relative strengths, limitations, and consistency in capturing the E-H-M continuum [256]. These studies emphasized that EMT is not a binary switch but a continuous and multidimensional spectrum requiring quantitative tools for robust clinical interpretation. Beyond prognostic stratification, EMT scoring approaches have been integrated with stemness and immune-related signatures, revealing that EMP is tightly linked to CSC traits and the modulation of the tumour immune microenvironment [257]. Such integrative scoring systems provide a systems-level framework to correlate E/M states with therapeutic resistance, immune evasion, and disease progression. From a theoretical perspective, the existence of stable hybrid states predicted by transcriptomic scoring metrics is supported by computational studies of multistable GRNs. Recent in silico studies have further refined this dynamical systems perspective by demonstrating that the enhanced plasticity of E/M states can be explained by specific topological features of EMT regulatory networks. In particular, network analyses have revealed the existence of mutually reinforcing “teams” of nodes corresponding to epithelial and mesenchymal programs, each characterized by dense intra-team positive feedback and inter-team antagonism [258, 259]. Fully epithelial or mesenchymal states are stabilized when one team dominates the regulatory landscape; in contrast, hybrid E/M states arise in configurations where neither team achieves complete dominance. This intermediate topological balance results in reduced structural reinforcement, rendering hybrid states more responsive to perturbations and therefore more plastic. Such findings provide a mechanistic explanation for the experimentally observed adaptability of E/M cells and link network topology directly to phenotypic stability and transition potential. Theoretical and computational frameworks describing multistability, bifurcations, and attractor landscapes have demonstrated how feedback loops and nonlinear regulatory interactions can generate multiple coexisting stable phenotypes, including epithelial, mesenchymal, and hybrid attractor states [260]. These approaches provide a rigorous dynamical systems foundation for understanding EMP, linking molecular regulatory circuits to phenotypic stability and transitions. Together, EMT scoring metrics and multistability-based modelling frameworks bridge quantitative transcriptomic data with dynamical systems theory, enabling a more precise and predictive characterization of EMP in patient samples.

The complexity and volume of multi-omics data often necessitate the use of machine learning (ML) and artificial intelligence (AI) to extract meaningful patterns and make predictions. Deep generative models, such as the UNAGI framework [246], power time-series single-cell data to model disease progression and simulate the effects of pharmacological perturbations. UNAGI learns disease-informed cell embeddings that capture the continuous transcriptomic shifts during EMT, enabling the identification of intermediate (hybrid) states and dynamic marker genes. In practical terms, UNAGI has been used to simulate in silico drug screening, predicting which compounds can revert hybrid or mesenchymal states toward a more epithelial phenotype, and prioritizing candidates for experimental validation. Complementing transcriptomics-based EMT scoring, a recent morphology-driven machine learning framework leveraging high-content Cell Painting imaging and organelle feature extraction (e.g., endoplasmic reticulum/mitochondrial texture metrics and cell-shape solidity) enables continuous, annotation-independent quantification of epithelial–mesenchymal plasticity, capturing EMT kinetics, hybrid states, and MET across different inducers and cancer types, with clear scalability for high-throughput phenotypic drug screening [261]. Beyond transcriptomics and molecular marker-based readouts, deep learning pipelines for morphology-driven phenotyping, benchmarked on brightfield intestinal organoid images, demonstrate that CNN architectures and Vision Transformers can accurately classify organoid developmental stages and morphological transitions reflective of epithelial plasticity, offering a scalable, automated imaging workflow applicable to EMT-like morphological changes across diverse organoid and cancer models [262].

Agent-based models and dynamic simulations provide a powerful framework for studying the temporal and spatial evolution of EMT and its hybrid states. For example, agent-based modelling has been used to simulate the transition between epithelial, hybrid, and mesenchymal states, demonstrating how cellular plasticity influences metastatic dissemination and therapeutic response [263]. Dynamic modelling approaches, including ordinary differential equations (ODEs) and Boolean networks, have been applied to simulate the regulatory circuits controlling EMT, predicting the existence of stable hybrid states and identifying key feedback loops that stabilize these phenotypes [264]. These models have revealed that E/M states can act as attractors in the regulatory landscape, indicating that hybrid phenotypes are not merely transient intermediates but can represent dynamically stable configurations determined by the structure of the underlying gene regulatory network, conferring robustness and adaptability to tumour cell populations. Integrative analysis of genomics, transcriptomics, proteomics, and metabolomics data provides a holistic view of EMT and its hybrid states. For example, SpidermiR [251] and UNAGI [246] both exemplify how multi-omics integration can uncover novel regulatory interactions and therapeutic targets. By combining data from multiple molecular layers, these approaches have revealed that E/M states are associated with unique gene signatures and regulatory networks that are not apparent from single-omic analyses [247].

 In silico approaches have significantly advanced the identification of novel EMT drivers, moving beyond canonical transcription factors to uncover complex regulatory networks involving microRNAs and other molecular effectors. Computational predictions are increasingly validated using sophisticated experimental models, such as GEMMs, PDOs, and patient-derived xenografts, which preserve tumour heterogeneity and microenvironmental complexity [44]. SpidermiR-based identification of miR-145-5p as a key regulator in prostate cancer was followed by experimental validation in cell lines and animal models, confirming its role in driving aggressive phenotypes [251]. Similarly, AI-driven models like UNAGI have been used to predict the response of tumour cells to anti-EMT therapies, guiding the prioritization of candidate compounds for preclinical testing [246]. The study of cellular plasticity, particularly the identification and characterization of E/M states, has been greatly facilitated by single-cell and spatial transcriptomics, which reveal the heterogeneity and dynamic nature of tumour cell populations. These insights are critical for the development of therapies targeting the supportive niches that sustain tumour progression. Despite their transformative potential, in silico models face significant challenges. The validation of computational predictions remains a bottleneck, often constrained by the resource-intensive nature of experimental work and the need for robust, scalable validation pipelines. The predictive accuracy of these models is fundamentally dependent on the quality and diversity of training data, with issues of data standardization and interoperability persisting as major obstacles [247]. Future progress will hinge on the integration of emerging technologies, including AI, single-cell multi-omics, and spatial transcriptomics, to build more realistic and comprehensive models of disease. The harmonization of high-resolution data with advanced analytics promises to enhance diagnostics, identify novel therapeutic targets, and enable the development of personalized therapies [246]. In silico methodologies have revolutionized the study of EMT, complementing traditional experimental approaches and enabling the integration and interpretation of complex, high-dimensional data. These computational tools facilitate the modelling of dynamic cellular processes, the identification of regulatory networks and biomarkers, and the simulation of therapeutic interventions. Their application holds significant promise for improving diagnosis, prognosis, and the development of targeted therapies, ultimately advancing the realization of personalized medicine in oncology.

Despite their transformative potential, each of these in silico approaches faces specific limitations that must be addressed to ensure clinical translatability (Table 2). For instance, while transcriptomics-based scoring metrics provide a quantitative framework, they often lack cross-platform consistency; a sample might be classified differently depending on the specific gene set or algorithm used, complicating the establishment of universal thresholds for ‘hybrid’ states. Furthermore, single-cell and spatial transcriptomics, while high-resolution, are frequently hampered by technical noise, ‘dropout’ events in gene detection, and the high costs associated with large-scale patient cohorts. Network-based and dynamic models (such as ODEs and Boolean networks) are inherently limited by the ‘curse of dimensionality’ and the scarcity of kinetic parameters for most regulatory interactions, often requiring simplifying assumptions that may overlook the stochastic nature of cellular transitions. Similarly, while AI and deep learning frameworks like UNAGI offer powerful predictive capabilities, they often operate as ‘black boxes’, making it difficult to extract the underlying biological logic behind a prediction. Moreover, the predictive accuracy of these models is fundamentally dependent on the quality and diversity of training data; issues of data standardization and interoperability persist as major obstacles, particularly when integrating multi-omics datasets from different laboratories. Finally, the validation of computational predictions remains a significant bottleneck, as the resource-intensive nature of experimental work with GEMMs or organoids cannot yet match the throughput of in silico screenings, necessitating more robust and scalable automated validation pipelines. 

**Table 2:** Summary of main in silico approaches for EMT research, their applications, representative studies/tools, strengths, and limitations

Approach	Key Question Answered	Applications	Representative Studies/Tools (PMID)	Strengths	Limitations
Network inference & GRN modelling	Which molecular circuits and master regulators drive EMT/hybrid states?	Identify EMT drivers, reconstruct regulatory circuits	SpidermiR [252], Network analysis [249]	Reveals key regulators, integrates multi-omics	Dependent on data quality, may miss context-specific effects
Single cell & spatial transcriptomics	Where are hybrid cells located and how does the microenvironment influence them?	Map EMT/hybrid states, analyse heterogeneity	Spatial transcriptomics [246], scRNA-seq [254–256]	High resolution, spatial context, captures plasticity	Costly, complex analysis, limited by tissue sampling
Machine learning & deep learning	Can we predict drug responses or identify new biomarkers from complex data?	Predict EMT states, drug response, biomarker discovery	UNAGI [247], ML review [248], ML organelles [262], ML morphology [263].	Handles high-dimensional data, enables in silico screening	Requires large datasets, interpretability challenges
Agent-based & dynamic modelling	What are the temporal trajectories and stability of EMT/MET transitions?	Simulate EMT transitions, study plasticity & evolution	Agent-based [264], E/M states [265]	Captures dynamics, models cell-cell interactions	Model assumptions, computationally intensive
Multi-omics integration	How do different molecular layers (DNA, RNA, protein) crosstalk during EMT?	Uncover novel signatures, link molecular layers	SpidermiR [252], UNAGI [247], ML review [248]	Holistic view, identifies crosstalk between pathways	Data integration challenges, batch effects

#### Therapeutic strategies to target EMP in cancer

Despite significant advances in tumour treatment, the acquisition of EMP features remains a key driver of cancer recurrence and chemoresistance. Targeting the EMT plasticity represents a promising approach in cancer therapy, given its critical role in tumour progression, metastasis, and resistance to conventional treatments [265]. However, the inherent plasticity and dynamic nature of EMT, particularly the existence of heterogeneous populations with completely epithelial or mesenchymal phenotypes and E/M states that confer both stemness and invasive capabilities, pose significant challenges to developing effective therapies [266]. EMP is regulated by complex signalling networks and transcriptional programs, including pathways such as TGF-β and PI3K/AKT, and transcription factors like SNAIL, SLUG, and ZEB1 [258]. Supporting this, Verstappe et al., using a Krt14CreTg/^+^-Trp53Fl/Fl mice, responsible for the cutaneous squamous cell carcinomas development, in association with the expression of ZEB2, a transcriptional factor modulating EMP, highlighted that the expression of ZEB2 induces the generation of heterogenic cancer cells population with a plastic phenotype [267]. Similarly, in a hybrid cellular model of NSCLC, characterized by a dynamic equilibrium between E-CADHERIN and SNAI2 expression, TGF-β1 treatment induced the acquisition of an EMP state and increased CD133 expression [268], a marker associated with highly metastatic lung CSCs [269]. These findings collectively indicate that EMP contributes to tumour progression and increased malignancy [268]. Considering this, the possibility of preventing or reversing the mesenchymal-like phenotype represents a critical approach to effective cancer therapy. Current therapeutic strategies targeting EMP focus on inhibiting upstream pathways involved in EMT, modulating metabolic reprogramming, epigenetic regulation, and co-targeting both the TME and intracellular mechanisms that sustain tumour growth and progression.

#### Intracellular signalling pathways

The inhibition of intracellular pathways is based on the use of inhibitors directed against key intracellular signalling pathways, including TGF-β, c-MET, NF-κB, EGFR, WNT, and Notch, as well as the use of ligand-neutralizing antibodies. The TGF-β pathway is well-known for its involvement in several cellular processes, such as proliferation, differentiation, and immunosurveillance [270]. Recently, its role in regulating EMP through the expression of EMT transcriptional factors has also emerged. In this context, bone morphogenetic protein-7 (BMP-7), a member of the TGF-β superfamily, plays a key role in the inhibition of EMT in melanoma, breast, and colon cancer [271–273]. In melanoma cells, treatment with BMP-7 induces MET, characterized by a reduction in the expression levels of TWIST and SNAIL [272]. In a recent study, Rastgar-Pouyani and colleagues have shown that the combinatorial treatment based on pirfenidone (PFD), which is an inhibitor of TGF-β, and paclitaxel (PTX), possesses a synergistic effect on the downregulation of breast cancer cell viability, colony-forming, and invasive capacity [274]. Importantly, the PFD + PTX combinatorial approach was able to reduce the expression of pluripotency transcription factors, and it mitigated the tumorigenic and metastatic ability of cancer cells. The HGF-c-MET axis is a potential therapeutic target primarily involved in cell proliferation. Additionally, it plays a significant role in regulating the EMT mechanism, ultimately enhancing the invasiveness of cancer cells [275]. Indeed, in vitro and in vivo studies have shown that HGF stimulation has been shown to upregulate the expression of Snail and Vimentin, key markers of the mesenchymal phenotype. Furthermore, activation of the HGF/c-MET signalling pathway in vitro induces chemoresistance. The treatment with crizotinib, a Met inhibitor, restores the chemotherapy sensitivity of small cell lung cancer cells, and alongside this reduces the expression of mesenchymal markers [276]. Despite its critical role, targeting the TGF-β pathway remains challenging due to its involvement in regulating stromal and immune cell behaviour [277, 278]. Nonetheless, inhibitors of this pathway are currently being evaluated in both preclinical and clinical settings. AXL, a receptor tyrosine kinase, is another receptor implicated in the regulation of EMT markers. Analysis of matched pairs of primary tumours and metastatic sites has shown that AXL expression is higher in metastases compared to primary tumours, suggesting that AXL is associated with poor prognosis in breast cancer patients and is crucial for the metastatic process. Silencing AXL in mesenchymal-like breast cancer cell lines has been shown to reduce both in vitro and in vivo tumour initiation and metastasis formation [279]. In addition, Koorstra and colleagues demonstrated that in pancreatic adenocarcinoma cells, AXL sustains the mesenchymal phenotype, with its silencing leading to the downregulation of key EMT transcription factors, such as SNAIL, TWIST, and SLUG [280]. In NSCLC, the upregulation of AXL and its ligand GAS6 is associated with EMT signature and resistance to EGFR and PI3K inhibitors. Notably, combined treatment with EGFR inhibitor erlotinib and the AXL inhibitor SGI-7079 counteracted drug resistance in mesenchymal-like NSCLC in a mouse xenograft model. These findings highlight AXL’s role in driving the EMT phenotype and CSC-like characteristics, suggesting it is a promising therapeutic target for eliminating CSCs that have undergone EMT [281]. The PI3K/AKT signalling pathway plays a crucial role in the induction of EMT through a SMAD-independent mechanism, leading to the activation of EMT-related transcription factors. Mechanistically, PI3K/AKT signalling inhibits the activity of GSK-3β, preventing the double phosphorylation of Snail at distinct serine residues, thereby blocking its cytoplasmic translocation (Ser 97, Ser 101) and proteasomal degradation (Ser108, Ser112, Ser116, and Ser120), and enhancing its transcriptional activity [282]. In lung cancer cells under hypoxic stress, overexpression of NETRIN-1, a laminin-related glycoprotein, activates the PI3K/AKT pathway, which in turn promotes cell proliferation, migration, and stimulates the EMT process [283]. Furthermore, Cassier et al. showed that in endometrial adenocarcinoma, the treatment with NP137, an anti-Netrin-1 antibody, not only induces tumour cell death but also effectively inhibits the expression of mesenchymal genes in preclinical models and patients with endometrial adenocarcinoma, highlighting its potential as a therapeutic agent [284]. In line with the key role of the PI3K pathway in the regulation of EMT, a recent study showed that the knockdown of the polycomb group factor 1 (PCGF1) led to reduced colorectal cancer cell proliferation, with the concomitant downregulation of EMT biomarkers, cancer cell invasive and migratory capacity, through the downregulation of Wnt/β‑Catenin and PI3K/AKT/mTOR pathways. Another intracellular regulator implicated in EMP is the EGFR. In a tyrosine-kinase inhibitor (TKI) resistant model of NSCLC, the treatment with metformin, an anti-diabetic drug, has been shown to overcome this resistance and inhibit EMT [285]. In conclusion, targeting intracellular signalling pathways involved in EMP represents a promising therapeutic strategy to counteract tumour progression, metastasis, and drug resistance. Key pathways such as TGF-β, HGF/c-MET, AXL, EGFR, and PI3K/AKT play central roles in regulating EMT and associated cancer stem cell–like properties. The inhibition of these pathways, either alone or in combination with conventional therapies, has demonstrated significant potential in reversing EMT, restoring drug sensitivity, and reducing tumour aggressiveness in preclinical and clinical settings.

#### Redifferentiation strategy

An effective strategy to target the EMT process is to promote the re-differentiation of mesenchymal cells into an epithelial phenotype, regulating the EMT marker expression, including E-CADHERIN, N-CADHERIN, and Vimentin. Fang et al. demonstrated in an in vitro study that lung cancer stem cells exhibit upregulation of BTBD7, a protein-protein interaction motif related to epithelial tissue remodelling and formation of branched organs [286, 287]. The upregulation of this protein induces a decrease in E-CADHERIN expression, contributing to the development of chemoresistance and facilitating the EMT. Importantly, silencing BTBD7 leads to a reduction in mesenchymal markers, specifically targeting cancer stem cells that are undergoing EMT [287]. In a recent work, Yan and colleagues showed that treatment with ß-elemene, a natural sesquiterpenic compound, mainly extracted from Curcuma wenyujin, reduced cell viability of breast cancer cells by inducing anoikis, downregulation of EMT markers such as MMPs, VIMENTIN, N-CADHERIN, TWIST1, and IGF, leading to a reversion of the EMT state in cancer cells [288]. Another example of EMT suppression and differentiation therapeutic approach is represented by Taxifolin (a natural, plant-derived flavonoid with powerful antioxidant, anti-inflammatory, and anti-tumour properties), which has been shown to inhibit proliferative and self-renewal of glioblastoma stem cells, and induce apoptosis, thus supporting its potential as a differentiation-based adjuvant therapy [289]. Interestingly, this work demonstrated that Taxifolin suppresses tumour growth, without toxicity, and boosts the therapeutic effect of temozolomide.

#### Targeting EMP-associated metabolism

In recent years, the interplay between EMP and the metabolic reprogramming in cancer cells has attracted significant interest as a key factor in cancer cell adaptation during the metastatic process. One example of this connection is the increased uptake of FFAs via the fatty acid translocase (CD36), which promotes Wnt/β-Catenin-mediated EMT in hepatocellular carcinoma. This is accompanied by elevated expression of mesenchymal markers, such as SNAIL, TWIST, ZEB, VIMENTIN, in HepG2 cells [144]. Importantly, chemical inhibition of CD36 has been shown to reduce the expression of these mesenchymal markers, further supporting the functional role of CD36-mediated FFAs uptake in driving EMT. A well-characterized aspect of this metabolic-EMT interplay is the dependence of cancer cells on glutamine metabolism to fuel the tricarboxylic acid (TCA) cycle [290], a process known to regulate EMT. In particular, in lung cancer, the suppression of glutaminolysis, the conversion of glutamine to glutamate mediated by glutaminase GLS1, has been shown to suppress the expression of the EMT-inducing transcription factor Snail, thereby reducing metastatic dissemination [291]. These findings highlight the potential of targeting lipid metabolism as a therapeutic strategy to inhibit EMT and suppress metastasis.

Consistent with this, emerging therapeutic strategies exploit the dynamic metabolic rewiring that sustains E/M states, invasion, stemness, and resistance. EMT shifts metabolism from glycolysis toward oxidative phosphorylation (OXPHOS), fatty acid oxidation, and oncometabolite accumulation like succinate, which stabilizes mesenchymal traits via HIF-1α stabilization, SUCNR1/GPR91 signalling, or ROS-mediated pathways, while extracellular vesicles (EVs) from cancer-associated fibroblasts transfer mtDNA or miRNAs to further reprogram mitochondrial function in tumour cells. In this scenario, repurposed metabolic inhibitors offer promise: MCT1 inhibitors (e.g., AZD3965, phase I/II trials) block lactate efflux critical for EMT migration; glutaminase inhibitors (CB-839) disrupt glutamine-fuelled anaplerosis in mesenchymal cells; and STAT3 inhibitors (e.g., napabucasin) revert partial EMT by normalizing OXPHOS. Emerging approaches include EV-engineered delivery of mitochondrial-targeted miRNAs or doxorubicin to OXPHOS-addicted cells, citrate supplementation to override hypoxic adaptation in low-glycolytic hepatocellular carcinomas (reducing HIF1α), and succinate pathway antagonists like SUCNR1 blockers or TRAP1 inhibitors to curb metastasis [292, 293]. Along similar lines, treatment with simvastatin, an inhibitor of 3-hydroxy-3-methylglutaryl–coenzyme A (HMG-CoA) reductase, in bladder cancer cells reduces the expression of the mesenchymal marker vimentin while increasing the expression of the epithelial marker E-CADHERIN. Additionally, this treatment decreases the proliferative, invasive, and migratory capacities of these cells [294]. In NSCLC, activation of the lipid metabolism enzyme fatty acid synthase (FASN) enhances the EMT induced by TGF-β, and the treatment with TVB-2640, a FASN inhibitor, reduces the EMT-associated metastatic potential in NSCLC cells resistant to cisplatin [131]. Another potential therapeutic target is glucose metabolism. In colorectal cancer cells, activation of the TGF-β/JNK/ATF2 signalling pathway increases GLUT-3 expression, thereby promoting EMT [295]. In this context, targeting this transporter could reduce the EMT-associated metastatic potential of cancer cells. Altogether, integrating metabolic targeting with conventional therapies may provide a more effective strategy to suppress EMP-driven tumour plasticity and improve clinical outcomes.

#### Targeting the epigenetic and post-transcriptional regulation of EMP

Epigenetic therapies have gained attention as an alternative strategy to reverse chromatin modifications that maintain EMT and stemness traits. Histone deacetylase inhibitors (HDACis) and DNMT inhibitors (DNMTis) can restore the expression of epithelial markers like E-CADHERIN and attenuate mesenchymal characteristics. For example, HDACis such as vorinostat have been shown to sensitize tumours to chemotherapy by modulating EMT plasticity, thereby overcoming resistance mechanisms [296, 297]. Moreover, the reduction of ZEB1 expression induced by HDACis or DNMTis is able to restore the sensitivity to chemotherapy in pancreatic cancer and osteosarcoma models. In the same way, the inhibition of ZEB1 resensitizes ovarian cancer cells to paclitaxel treatment [298]. In parallel, the use of monoclonal antibodies specific for crucial mediators of EMP is emerging as a novel and efficient therapeutic approach. Simanovich et al. have recently demonstrated that a monoclonal antibody specific for EMMPRIN (hMR18-mAb), a membrane glycoprotein implicated in cell–cell interactions, proliferation, angiogenesis, and EMT, loaded on U937 monocytic-like cells, inhibits the EMT process in breast and oral squamous cancer cells. This effect is mediated by a reduced release of EMT-inducer cytokines by macrophages, an increased E-CADHERIN, and reduced vimentin expression. Moreover, the treatment with hMR18-mAb was sufficient to reduce the onset of dormant phenotypes, which are often associated with the metastatic potential of cancer cells [299]. Recently, the role of miRNA, a single-stranded non-coding RNA, has emerged in the context of EMT transition [300]. As evidenced by the miR-200 family, microRNAs can suppress EMT by targeting EMT-regulating transcription factors such as ZEB1 and ZEB2 [62]. Ma et al. reported that in gemcitabine-resistant pancreatic cancer cells, high expression of this miRNA induces a mesenchymal state, whereas inhibition of miR-223 activates MET and reduces cell motility and invasion capacity [301].

#### TME reprogramming

A complementary strategy focuses on targeting the TME, which plays a crucial role in sustaining EMP and promoting tumour progression. By modulating both cellular and non-cellular components of the TME, these strategies aim to interfere with the external cues that sustain mesenchymal-like phenotypes, ultimately enhancing the efficacy of conventional and targeted anticancer treatments [302, 303]. Therapies targeting CAFs include fibroblast activation protein (FAP) inhibitors or CAR-T cells for direct depletion [304, 305], TGF-β pathway blockers (e.g., Galunisertib, AVID200) to prevent activation and EMT induction, and reprogramming agents like vitamin D analogues or all-trans retinoic acid (ATRA) to revert myCAFs to quiescent states, thereby reducing cytokine secretion and ECM remodelling that sustain EMP [306]. Losartan, an angiotensin II receptor antagonist, curbs collagen deposition and CAF contractility, improving drug penetration in preclinical models [307]. Anti-angiogenic agents such as bevacizumab (anti-VEGF) or sunitinib normalize tumour vasculature, alleviating hypoxia-driven HIF-1α/EMP signalling from ECs [308]. Interestingly, vascular normalization therapies enhance the efficacy of immunotherapy by improving T-cell infiltration. Several pieces of evidence have demonstrated that the acquisition of EMP phenotype modulated the TME toward an immunosuppressive state. Additionally, in different tumours, it was demonstrated that the EMP induces an enhancement of immune checkpoint expression, such as PD-1 and PD-L1 [309, 310]. In this scenario, phase I/II trials demonstrate safety and synergy with PD-1 inhibitors by restoring TME immunosurveillance [211, 311–314]. In a mouse model, the use of Galunisertib (LY2157299), an inhibitor of TGF-β, induces the reduction of tumour growth and metastasis formation, enhancing the immune cells’ response following anti-PD-1/PD-L1 treatment [315]. Regarding the immune cell compartment, TAMs can be depleted or reprogrammed to M1 antitumor phenotypes using CSF1R inhibitors (e.g., pexidartinib) or CD40 or TLR agonists [316, 317]. This treatment reduces TGF-β/IL-6 output, fuelling STAT3/Snail-mediated EMP. On the other hand, MDSCs succumb to low-dose gemcitabine/5-FU or ATRA to block recruitment/differentiation via CCL2/ARG1 inhibition, while Tregs are depleted by anti-CD25 antibodies or targeted via PI3Kδ/γ inhibitors, alleviating immunosuppression and partial EMT stabilization [318]. Targeting neurotrophic factors and neural cells in tumours offers promising strategies to disrupt tumour plasticity, EMT, and progression driven by tumour-neural crosstalk. Small-molecule tyrosine kinase inhibitors like Larotrectinib and entrectinib potently block TrkA/B/C signalling, reducing BDNF/NGF-mediated CSC enrichment and EMT in cancers such as lung, breast, and neuroblastoma. These FDA-approved agents (NTRK fusion-positive tumours) show clinical efficacy in shrinking Trk-driven lesions while overcoming plasticity-induced resistance; ongoing trials combine them with chemotherapy to target perineural invasion [210, 319]. Differently, monoclonal antibodies or decoy receptors against p75NTR can be used to interrupt low-affinity neurotrophins binding, suppressing SLUG induction and invasion in breast/glioma models [320–322]. Anti-BDNF or anti-NGF antibodies (e.g., tanezumab analogues repurposed for oncology) neutralize circulating ligands, curbing tumour angiogenesis and motility [323]. Surgical or pharmacological denervation (e.g., botulinum neurotoxin) severs peritumoral nerves, halting the release of neurotransmitters and neurotrophins that fuel tumour plasticity and EMT [324, 325]. Finally, the combinatorial therapy based on the use of neurotrophin receptor inhibitors and EMT reversers (e.g., TGF-β blockers), or metabolic disruptors, could exploit synthetic lethality in E/M states. Future strategies may pair these with CAR-T cells engineered against neural antigens to eliminate innervating glia/Schwann cells. Finally, ECM stiffness is mitigated by losartan or relaxin to decrease collagen crosslinking/HA accumulation, inhibiting integrin-FAK-YAP/TAZ mechanotransduction that locks E/M states; LOXL2 inhibitors (e.g., simtuzumab) prevent fibrosis [326, 327]. These approaches enhance chemotherapy/radiotherapy penetration and cooperate with immunotherapies like ICB by easing physical barriers and T-cell exhaustion. Targeting the TME offers a synergistic approach to limit EMP and its associated pro-tumorigenic effects, representing a strategy to overcome resistance, limit tumour plasticity, and improve overall clinical outcomes.

In conclusion, while the therapeutic strategies described above underscore the breadth of potential targets within EMP-associated signalling, metabolism, epigenetics, and the TME, a fundamental challenge remains: the very plasticity that renders EMP clinically dangerous could potentially allow cancer cells to circumvent therapeutic pressure. Unlike static oncogenic drivers, EMP is inherently dynamic. Cancer cells do not simply occupy a fixed epithelial or mesenchymal state but continuously shift along a phenotypic spectrum in response to microenvironmental cues, including those inadvertently generated by treatment itself. Therefore, targeting a single EMT state or a specific molecular mediator may be insufficient, as surviving cells can readily transition toward alternative E/M states, effectively escaping therapeutic intervention. This adaptive phenotypic switching, rather than classical genetic resistance, represents one of the most formidable obstacles in anti-EMP therapy, demanding strategies that account not only for the identity of the target, but for the fluidity of the system being targeted.

#### Unsolved questions and future directions

Despite significant advances, the study of EMP continues to raise fundamental questions regarding its regulation and functional relevance in tumour progression. Clarifying how EMP operates across distinct tumour types, microenvironmental contexts, and disease stages remains a central challenge, as does defining the contribution of transient or hybrid phenotypic states to metastatic dissemination and therapeutic resistance. The development of more sophisticated lineage-tracing systems, spatial transcriptomic approaches, and integrative multi-omic analyses will be critical to dissect these dynamic processes in vivo. Moreover, integrating EMP research with studies of immune modulation, stromal crosstalk, and metabolic adaptation may illuminate how these interconnected layers collectively shape tumour evolution. A mechanistic and context-dependent understanding of EMP may ultimately reconcile current discrepancies in the field and provide the basis for novel therapeutic strategies designed to constrain malignant plasticity, enhance treatment sensitivity, and limit disease recurrence.

## Conclusion

Based on accumulating evidence, this review establishes EMP as the main driver of CSC self-renewal, intratumor heterogeneity, metastatic dissemination, and resistance to therapies across diverse malignancies. EMP states, enriched in CSCs, confer higher tumorigenicity through balanced epithelial-mesenchymal traits, dynamic metabolic shifts (glycolysis/OXPHOS/lipid rewiring), and TME synergies involving CAFs, TAMs, endothelial cells, immune cells, neural cells, and ECM stiffness. Sophisticated tools, from dual-fluorescent reporters and organoids to spatial transcriptomics, intravital microscopy, and AI-driven models, have recently helped in mapping EMP trajectories, validating its evolutionary fitness and biomarker potential. However, enduring gaps continue to hinder translation. Despite these current challenges, therapeutic optimism remains: single‑pathway inhibitors (e.g., targeting TGF‑β or Notch) often fail because redundancy and cellular plasticity enable compensatory adaptations, and metabolic disruptors can cause off‑target toxicity unless they achieve true CSC specificity. Future advances demand multi-omics integration, patient-derived avatars for combinatorial regimens (e.g., EMP-blockers + immunotherapy), and robust biomarkers to stratify hybrid-enriched subsets, ultimately dismantling this adaptive vulnerability to avert relapse. Given the redundancy and complexity of EMT regulatory networks, combination therapies targeting multiple facets of EMT alongside conventional chemotherapies, targeted therapies, or immunotherapies are likely necessary to prevent treatment resistance and metastatic relapse [328]. Rational design guided by biomarkers capable of identifying EMT and CSC states will improve patient stratification and therapeutic outcomes. In summary, therapeutic strategies targeting EMT and particularly E/M states in cancer encompass inhibition of signalling pathways, epigenetic modulation, metabolic disruption, and microenvironmental remodelling. As the understanding of EMT plasticity deepens, new, precise, and effective treatments aimed at eradicating the most aggressive CSC-enriched tumour cell populations are anticipated to emerge, offering renewed hope for combating metastatic disease.

## Data Availability

No datasets were generated or analysed during the current study.

## Abbreviations

2DG: 2-deoxy-D-glucose
ACC: Acetyl-CoA carboxylase
ACLY: ATP citrate lyase
ACP: Acyl Carrier Protein
ACSL: Acyl-CoA synthetase long-chain
ACSL: Acyl-CoA synthetases
ADP: Adenosine diphosphate
AhR: Aryl Hydrocarbon Receptor
AI: Artificial Intelligence
ALDH: Aldehyde dehydrogenase
ALDH1A3: Aldehyde Dehydrogenase 1 Family, Member A3
ALDOA: fructose-bisphosphate aldolase A
AMP: Adenosine monophosphate
AMPA: Alpha-Amino-3-hydroxy-5-methyl-4-isoxazolepropionic acid
AMPK: AMP-activated protein kinase
ARG1: Arginase-1
ARNT: Aryl hydrocarbon Receptor Nuclear Translocator
ATGL: Adipose Triglyceride Lipase
ATRA: all-trans retinoid acid
AXL: Receptor Tyrosine Kinase
BCSCs: Breast cancer stem cells
BDNF: Brain- derived neurotrophic factor
BLI: Bioluminescence imaging
BMP-7: Bone morphogenetic protein-7
BTBD7: BTB/POZ domain-containing protein 7
c-MET: Hepatocyte growth factor receptor
c-Myc: cellular Myelocytomatosis protein
CAFs: Cancer-associated fibroblasts
CAMK2: Calmodulin-dependent protein kinase II
CAR-T: Chimeric Antigen Receptor T-cell
CAV1: Caveolin-1
CCL2: C-C motif chemokine ligand 2
CDH1: Epithelial cadherin, E-CADHERIN
CDH2: Neural cadherin, N-CADHERIN
CHD1: Chromodomain Helicase DNA Binding Protein 1
ChREBP: Carbohydrate-Responsive Element-Binding Protein
CK8: Cytokeratin 8
CLDN: Claudin
CLRKeap1: Cullin-RING E3 ubiquitin ligase complex
CPT1: Carnitine palmitoyltransferase 1
CRC: Colorectal Cancer
CreER: Cre recombinase fused with Estrogen Receptor
CRIF1: CR-6 interacting factor 1
CSCs: Cancer Stem Cells
CSF1R: Colony Stimulating Factor 1 Receptor
CT: Computed tomography
CTCs: Circulating Tumour Cells
D1x-2: Distal-less homeobox-2
DNMT: DNA- methyltransferase
DNMTis: DNA methyltransferase inhibitors
DPYD: Dihydropyridine dehydrogenase
DVL3: Disheveled 3
E-H-M: epithelial–hybrid–mesenchymal
E/M: Hybdrid Epithelial/Mesenchymal
ECM: Extracellular matrix
EMP: Epithelial Mesenchymal Plasticity
EMT: Epithelial-mesenchymal transition
EMT-TFs: EMT-inducing transcription factors
ENO: Enolase
EPCAM: Epithelial Cell Adhesion Molecule
ESRP1: Epithelial Splicing Regulatory Protein 1
EZH2: Enhancer of zeste homolog 2
FAK: Focal adhesion kinase
FAO: Fatty acid beta-oxidation
FAP: Fibroblast Activation protein
FASN: Fatty acid synthase
FAT1: FAT Atypical Cadherin 1
FBP1: fructose-1,6-bisphosphatase 1
FDA: Food Drug Association
FFAs: Free fatty acids
FH: Fumarate hydratase
FRET: Förster Resonance Energy Transfer
Fsp1: Fibroblast-specific protein 1
GADPH: Glyceraldehyde-3-Phosphate Dehydrogenase
GDH: Glutamate dehydrogenase
GEMMs: Genetically engineered mouse models
GLN: Glutamine
GLS1: Glutaminase 1
GLUT1: Glucose Transporter 1
GPR91: G protein-coupled receptor 91
GPx2: Glutathione peroxidase 2
GRHL2: Grainyhead-like 2
GRNs: gene regulatory networks
GTP: Guanosine-5′-triphosphate
H3K27me3: Histone H3 protein trymethylated Lysine 27
H3K9me3: Histone H3 protein trymethylated Lysine 9
HDACi: Histone deacetylase inhibitor
HIF-1α: Hypoxia-Inducible Factor 1-alpha
HK2: Hexokinase 2
HMG-CoA: 3-hydroxy- 3-methylglutaryl–coenzyme A
HMGA1: High Mobility Group A1
HOTAIR: HOX transcript antisense RNA
HSL: Hormone-Sensitive Lipase
ICB: Immune checkpoint blockage
IDH: Isocitrate dehydrogenase
IGF: Insulin-like growth factors
integrin αvβ3: αvβ3 vitronectin receptor
integrin-α5β1: Fibronectin Receptor
IVM: Intravital microscopy
JAK2: Janus Kinase 2
KLF4: Krüppel-like factor 4
Krt14CreTg: Keratin 14 Cre recombinase Transgenic
LDHA: Lactate dehydrogenase A
LEF: Lymphoid enhancer factor
LIN28/let-7: Lin28 homolog-mediated control of Lethal-7 maturation
LKB1: Liver Kinase B1
LOXL2: Lysyl oxidase homolog 2
LSH: Lymphoid-Specific Helicase
MALAT1: Metastasis Associated Lung Adenocarcinoma Transcript 1
MAPK: Mitogen-activated protein kinase
MCT1: Monocarboxylate Transporter 1
MDSCs: Myeloid-derived suppressor cells
MET: Mesenchymal-epithelial transition
MGL: Monoacylglycerol lipase
ML: Machine Learning
MMP: Matrix metalloproteinase
mtDNA: Mitochondrial DNA
mTOR: Mechanistic Target of Rapamycin
NAD+: Nicotinamide adenine dinucleotide
NADPH: Nicotinamide adenine dinucleotide phosphate
NF-κB: Nuclear factor kappa-B
NGF: Nerve growth factor
NICD: Notch Intracellular Domain
NK: Natural Killer
Notch-Dll4-JAG1: Notch- Delta-like 4 ligand -Jagged 1 ligand
NPC: nasopharyngeal carcinoma
NRF2: Nuclear factor erythroid 2-related factor 2
NRTN: Neurturin
NSCL: Non-small cell lung cancer
NTRK: Neurotrophic Tyrosine Receptor Kinase
OCT4: Octamer Binding Transcription Factor 4
OVOL2: Ovo Like Zinc Finger 2
OXPHOS: Oxidative phosphorylation
p75NTR: 75 kDa neurotrophin receptor
PCGF1: polycomb group factor 1
PD-1: Programmed cell Death protein 1
PD-L1: Programmed death-ligand 1
PDAC: Pancreatic ductal adenocarcinoma
PDGF: Platelet-derived growth factor
PDGF-BB: Platelet-Derived Growth Factor-BB
PDOs: Patient-derived organoids
PFD: Pirfenidone
PFKFB-3: 6-phosphofructo-2-kinase/fructose-2,6-biphosphatase 3
PFKP: Phosphofructokinase, platelet
PGC-1α: Proliferator-activated receptor gamma coactivator 1-alpha
PGI: Phosphoglucose isomerase
PKCα: Protein kinase C alpha
PKM2: Pyruvate Kinase M2
PPARs: Peroxisome proliferator-activated receptors
PPP: Pentose phosphate pathway
PTX: Paclitaxel
RAS: Rat sarcoma virus family
Rho: Rho GTPase family
RNF4: Ring finger protein 4
ROS: Reactive oxygen species
SCD: Stearoyl-CoA desaturase
SCF: SKP1-Cullin-F box (E3-ubiquitin ligase complex)
scRNA-seq: Single-cell RNA sequencing
SDHAF2: Succinate Dehydrogenase Complex Assembly Factor 2
SDHB: Succinate dehydrogenase complex iron-sulfur subunit B
Siah2: Seven in absentia homolog 2 (E3-ubiquitin protein ligase)
SMAD: SMA (Caenorhabditis elegans worm ) gene and MAD (Mothers Against Decapentaplegic ) gene fusion
SOX2: Sex-determining Region Y-related High mobility group-box 2
Src: Proto-oncogene tyrosine-protein kinase
SREBP1: Sterol Regulatory Element-Binding Protein 1
STAT3: Signal Transducer and Activator of Transcription 3
SUCNR1: Succinate receptor 1
TAM: Tumor-associated macrophage
TAZ: Transcriptional coactivator with PDZ-binding motif
TCA: Tricarboxylic Acid Cycle
TCF: T-cell factor
TKI: Tyrosine Kinase Inhibitors
TLR: Toll-like receptor
TME: Tumor microenvironment
TNBC: Triple negative breast cancer
TNF-⍺: Tumor Necrosis Factor-alpha
TRAP1: Tumor necrosis factor receptor-associated protein 1
Trk: Tropomyosin receptor kinase B
Trp53Fl: “floxed” Transformation-related protein 53
VEGF: Vascular endothelial Growth Factor
VIM: Vimentin
Wnt: Wingless-related integration site
YAP1: Yes-associated protein 1
ZEB: Zinc finger E-box-binding homeobox
ZO-1: Zonula Occludens-1
α-KG: alpha-ketoglutarate
α-SMA: Alpha-smooth muscle actin
β-catenin: Beta catenin
β-TrCP: Beta-transducin repeat-containing protein

## References

[1] Bonnet D, Dick JE. Human acute myeloid leukemia is organized as a hierarchy that originates from a primitive hematopoietic cell. Nat Med. 1997;3:730–7. 10.1038/nm0797-730. 9212098 10.1038/nm0797-730

[2] Thankamony AP, Saxena K, Murali R, Jolly MK, Nair R. Cancer stem cell plasticity - a deadly deal. Front Mol Biosci. 2020;7:79. 10.3389/fmolb.2020.00079. 32426371 10.3389/fmolb.2020.00079 PMC7203492

[3] Turdo A, Porcelli G, D’Accardo C, Franco SD, Verona F, Forte S, et al. Metabolic escape routes of cancer stem cells and therapeutic opportunities. Cancers (Basel). 2020;12:1436. 10.3390/cancers12061436. 32486505 10.3390/cancers12061436 PMC7352619

[4] Lo Iacono M, Gaggianesi M, Bianca P, Brancato OR, Muratore G, Modica C, et al. Destroying the shield of cancer stem cells: natural compounds as promising players in cancer therapy. JCM. 2022;11:6996. 10.3390/jcm11236996. 36498571 10.3390/jcm11236996 PMC9737492

[5] Loh J-J, Ma S. Hallmarks of cancer stemness. Cell Stem Cell. 2024;31:617–39. 10.1016/j.stem.2024.04.004. 38701757 10.1016/j.stem.2024.04.004

[6] Guo Q, Zhou Y, Xie T, Yuan Y, Li H, Shi W, et al. Tumor microenvironment of cancer stem cells: Perspectives on cancer stem cell targeting. Genes Dis. 2024;11:101043. 10.1016/j.gendis.2023.05.024. 38292177 10.1016/j.gendis.2023.05.024 PMC10825311

[7] Gaggianesi M, Di Franco S, Pantina VD, Porcelli G, D’Accardo C, Verona F, et al. Messing up the cancer stem cell chemoresistance mechanisms supported by tumor microenvironment. Front Oncol. 2021;11:702642. 10.3389/fonc.2021.702642. 34354950 10.3389/fonc.2021.702642 PMC8330815

[8] Saxena K, Jolly MK, Balamurugan K. Hypoxia, partial EMT and collective migration: emerging culprits in metastasis. Transl Oncol. 2020;13:100845. 10.1016/j.tranon.2020.100845. 32781367 10.1016/j.tranon.2020.100845 PMC7419667

[9] Abd GM, Laird MC, Ku JC, Li Y. Hypoxia-induced cancer cell reprogramming: a review on how cancer stem cells arise. Front Oncol. 2023;13:1227884. 10.3389/fonc.2023.1227884. 37614497 10.3389/fonc.2023.1227884 PMC10442830

[10] Eastham AM, Spencer H, Soncin F, Ritson S, Merry CLR, Stern PL, et al. Epithelial-mesenchymal transition events during human embryonic stem cell differentiation. Cancer Res. 2007;67:11254–62. 10.1158/0008-5472.CAN-07-2253. 18056451 10.1158/0008-5472.CAN-07-2253

[11] Acloque H, Adams MS, Fishwick K, Bronner-Fraser M, Nieto MA. Epithelial-mesenchymal transitions: the importance of changing cell state in development and disease. J Clin Invest. 2009;119:1438–49. 10.1172/JCI38019. 19487820 10.1172/JCI38019 PMC2689100

[12] Huang Y, Hong W, Wei X. The molecular mechanisms and therapeutic strategies of EMT in tumor progression and metastasis. J Hematol Oncol. 2022;15:129. 10.1186/s13045-022-01347-8. 36076302 10.1186/s13045-022-01347-8 PMC9461252

[13] Din ZU, Cui B, Wang C, Zhang X, Mehmood A, Peng F, et al. Crosstalk between lipid metabolism and EMT: emerging mechanisms and cancer therapy. Mol Cell Biochem. 2025;480:103–18. 10.1007/s11010-024-04995-1. 38622439 10.1007/s11010-024-04995-1

[14] Singh A, Gupta R, Kulshreshtha R. Capturing the variations in mutant p53-driven regulatory networks in breast cancer subtypes, its clinical relevance and a novel association with androgen receptor and EMT. IUBMB Life. 2026;78:e70081. 10.1002/iub.70081. 41472467 10.1002/iub.70081

[15] Hanahan D. Hallmarks of cancer—Then and now, and beyond. Cell. 2026. 10.1016/j.cell.2025.12.049. S0092867425014989. 41616779 10.1016/j.cell.2025.12.049

[16] Yang J, Antin P, Berx G, Blanpain C, Brabletz T, Bronner M, et al. Author Correction: Guidelines and definitions for research on epithelial-mesenchymal transition. Nat Rev Mol Cell Biol. 2021;22:834. 10.1038/s41580-021-00428-9. 34654908 10.1038/s41580-021-00428-9 PMC8604719

[17] Ye X, Weinberg RA, Epithelial-Mesenchymal Plasticity. A Central regulator of cancer progression. Trends Cell Biol. 2015;25:675–86. 10.1016/j.tcb.2015.07.012. 26437589 10.1016/j.tcb.2015.07.012 PMC4628843

[18] Todaro M, Gaggianesi M, Catalano V, Benfante A, Iovino F, Biffoni M, et al. CD44v6 Is a marker of constitutive and reprogrammed cancer stem cells driving colon cancer metastasis. Cell Stem Cell. 2014;14:342–56. 10.1016/j.stem.2014.01.009. 24607406 10.1016/j.stem.2014.01.009

[19] Canciello A, Cerveró-Varona A, Peserico A, Mauro A, Russo V, Morrione A, et al. In medio stat virtus: Insights into hybrid E/M phenotype attitudes. Front Cell Dev Biol. 2022;10:1038841. 10.3389/fcell.2022.1038841. 36467417 10.3389/fcell.2022.1038841 PMC9715750

[20] Jolly MK, Tripathi SC, Somarelli JA, Hanash SM, Levine H. Epithelial/mesenchymal plasticity: how have quantitative mathematical models helped improve our understanding? Mol Oncol. 2017;11:739–54. 10.1002/1878-0261.12084. 28548388 10.1002/1878-0261.12084 PMC5496493

[21] Verstappe J, Berx G. A role for partial epithelial-to-mesenchymal transition in enabling stemness in homeostasis and cancer. Semin Cancer Biol. 2023;90:15–28. 10.1016/j.semcancer.2023.02.001. 36773819 10.1016/j.semcancer.2023.02.001

[22] Den Hollander P, Castaneda M, Vasaikar SV, Maddela JJ, Gould C, Demestichas BR, et al. EMT-induced stem cell and mesenchymal programs can be decoupled via cell division and ESRP1-dependent mechanisms. iScience. 2026;29:114284. 10.1016/j.isci.2025.114284. 41503216 10.1016/j.isci.2025.114284 PMC12768877

[23] Pastushenko I, Blanpain C. EMT Transition states during tumor progression and metastasis. Trends Cell Biol. 2019;29:212–26. 10.1016/j.tcb.2018.12.001. 30594349 10.1016/j.tcb.2018.12.001

[24] Lambert AW, Weinberg RA. Linking EMT programmes to normal and neoplastic epithelial stem cells. Nat Rev Cancer. 2021;21:325–38. 10.1038/s41568-021-00332-6. 33547455 10.1038/s41568-021-00332-6

[25] Jolly MK, Boareto M, Huang B, Jia D, Lu M, Ben-Jacob E, et al. implications of the hybrid epithelial/mesenchymal phenotype in metastasis. Front Oncol. 2015;5:155. 10.3389/fonc.2015.00155. 26258068 10.3389/fonc.2015.00155 PMC4507461

[26] Jolly MK, Jia D, Boareto M, Mani SA, Pienta KJ, Ben-Jacob E, et al. Coupling the modules of EMT and stemness: a tunable stemness window model. Oncotarget. 2015;6:25161–74. 10.18632/oncotarget.4629. 26317796 10.18632/oncotarget.4629 PMC4694822

[27] Celià-Terrassa T, Kang Y. Distinctive properties of metastasis-initiating cells. Genes Dev. 2016;30:892–908. 10.1101/gad.277681.116. 27083997 10.1101/gad.277681.116 PMC4840296

[28] Nieto MA, Huang RY-J, Jackson RA, Thiery JPEMT. 2016. Cell. 2016;166:21–45. 10.1016/j.cell.2016.06.028 10.1016/j.cell.2016.06.028 27368099

[29] Shibue T, Weinberg RA. EMT, CSCs, and drug resistance: the mechanistic link and clinical implications. Nat Rev Clin Oncol. 2017;14:611–29. 10.1038/nrclinonc.2017.44. 28397828 10.1038/nrclinonc.2017.44 PMC5720366

[30] Batlle E, Clevers H. Cancer stem cells revisited. Nat Med. 2017;23:1124–34. 10.1038/nm.4409. 28985214 10.1038/nm.4409

[31] Liao T-T, Yang M-H. Hybrid epithelial/mesenchymal state in cancer metastasis: clinical significance and regulatory mechanisms. Cells. 2020;9:623. 10.3390/cells9030623. 32143517 10.3390/cells9030623 PMC7140395

[32] Lamouille S, Xu J, Derynck R. Molecular mechanisms of epithelial-mesenchymal transition. Nat Rev Mol Cell Biol. 2014;15:178–96. 10.1038/nrm3758. 24556840 10.1038/nrm3758 PMC4240281

[33] Ozawa M, Kobayashi W. Reversibility of the Snail-induced epithelial-mesenchymal transition revealed by the Cre-loxP system. Biochem Biophys Res Commun. 2015;458:608–13. 10.1016/j.bbrc.2015.02.012. 25681770 10.1016/j.bbrc.2015.02.012

[34] Xie Y, Wang X, Wang W, Pu N, Liu L. Epithelial-mesenchymal transition orchestrates tumor microenvironment: current perceptions and challenges. J Transl Med. 2025;23:386. 10.1186/s12967-025-06422-5. 40176117 10.1186/s12967-025-06422-5 PMC11963649

[35] Aggarwal V, Montoya CA, Donnenberg VS, Sant S. Interplay between tumor microenvironment and partial EMT as the driver of tumor progression. iScience. 2021;24:102113. 10.1016/j.isci.2021.102113. 33659878 10.1016/j.isci.2021.102113 PMC7892926

[36] Brabletz S, Schuhwerk H, Brabletz T, Stemmler MP. Dynamic EMT: a multi-tool for tumor progression. EMBO J. 2021;40:e108647. 10.15252/embj.2021108647. 34459003 10.15252/embj.2021108647 PMC8441439

[37] Akhmetkaliyev A, Alibrahim N, Shafiee D, Tulchinsky E. EMT/MET plasticity in cancer and Go-or-Grow decisions in quiescence: the two sides of the same coin? Mol Cancer. 2023;22:90. 10.1186/s12943-023-01793-z. 37259089 10.1186/s12943-023-01793-z PMC10230810

[38] Aiello NM, Maddipati R, Norgard RJ, Balli D, Li J, Yuan S, et al. EMT subtype influences epithelial plasticity and mode of cell migration. Dev Cell. 2018;45:681–e6954. 10.1016/j.devcel.2018.05.027. 29920274 10.1016/j.devcel.2018.05.027 PMC6014628

[39] Vilchez Mercedes SA, Bocci F, Ahmed M, Eder I, Zhu N, Levine H, et al. Nrf2 Modulates the hybrid epithelial/mesenchymal phenotype and notch signaling during collective cancer migration. Front Mol Biosci. 2022;9:807324. 10.3389/fmolb.2022.807324. 35480877 10.3389/fmolb.2022.807324 PMC9037689

[40] Lüönd F, Sugiyama N, Bill R, Bornes L, Hager C, Tang F, et al. Distinct contributions of partial and full EMT to breast cancer malignancy. Dev Cell. 2021;56:3203–e322111. 10.1016/j.devcel.2021.11.006. 34847378 10.1016/j.devcel.2021.11.006

[41] Gooding AJ, Schiemann WP. Epithelial-mesenchymal transition programs and cancer stem cell phenotypes: mediators of breast cancer therapy resistance. Mol Cancer Res. 2020;18:1257–70. 10.1158/1541-7786.MCR-20-0067. 32503922 10.1158/1541-7786.MCR-20-0067 PMC7483945

[42] Tan TZ, Miow QH, Miki Y, Noda T, Mori S, Huang RY-J, et al. Epithelial-mesenchymal transition spectrum quantification and its efficacy in deciphering survival and drug responses of cancer patients. EMBO Mol Med. 2014;6:1279–93. 10.15252/emmm.201404208. 25214461 10.15252/emmm.201404208 PMC4287932

[43] Wilson MM, Weinberg RA, Lees JA, Guen VJ. Emerging mechanisms by which EMT programs control stemness. Trends Cancer. 2020;6:775–80. 10.1016/j.trecan.2020.03.011. 32312682 10.1016/j.trecan.2020.03.011

[44] Jolly MK, Somarelli JA, Sheth M, Biddle A, Tripathi SC, Armstrong AJ, et al. Hybrid epithelial/mesenchymal phenotypes promote metastasis and therapy resistance across carcinomas. Pharmacol Ther. 2019;194:161–84. 10.1016/j.pharmthera.2018.09.007. 30268772 10.1016/j.pharmthera.2018.09.007

[45] Parodi M, Centonze G, Murianni F, Orecchia P, Andriani F, Roato I, et al. Hybrid epithelial-mesenchymal status of lung cancer dictates metastatic success through differential interaction with NK cells. J Immunother Cancer. 2024;12:e007895. 10.1136/jitc-2023-007895. 38458638 10.1136/jitc-2023-007895 PMC10921513

[46] Sahoo S, Nayak SP, Hari K, Purkait P, Mandal S, Kishore A, et al. Immunosuppressive traits of the hybrid epithelial/mesenchymal phenotype. Front Immunol. 2021;12:797261. 10.3389/fimmu.2021.797261. 34975907 10.3389/fimmu.2021.797261 PMC8714906

[47] Lecharpentier A, Vielh P, Perez-Moreno P, Planchard D, Soria JC, Farace F. Detection of circulating tumour cells with a hybrid (epithelial/mesenchymal) phenotype in patients with metastatic non-small cell lung cancer. Br J Cancer. 2011;105:1338–41. 10.1038/bjc.2011.405. 21970878 10.1038/bjc.2011.405 PMC3241564

[48] Zhao Q, Li B, Gao Q, Luo Y, Ming L. Prognostic value of epithelial-mesenchymal transition circulating tumor cells in female breast cancer: a meta-analysis. Front Oncol. 2022;12:1024783. 10.3389/fonc.2022.1024783. 36530995 10.3389/fonc.2022.1024783 PMC9749886

[49] Qi L-N, Xiang B-D, Wu F-X, Ye J-Z, Zhong J-H, Wang Y-Y, et al. Circulating tumor cells undergoing emt provide a metric for diagnosis and prognosis of patients with hepatocellular carcinoma. Cancer Res. 2018;78:4731–44. 10.1158/0008-5472.CAN-17-2459. 29915159 10.1158/0008-5472.CAN-17-2459

[50] Williams ED, Gao D, Redfern A, Thompson EW. Controversies around epithelial-mesenchymal plasticity in cancer metastasis. Nat Rev Cancer. 2019;19:716–32. 10.1038/s41568-019-0213-x. 31666716 10.1038/s41568-019-0213-x PMC7055151

[51] George JT, Jolly MK, Xu S, Somarelli JA, Levine H. Survival outcomes in cancer patients predicted by a partial emt gene expression scoring metric. Cancer Res. 2017;77:6415–28. 10.1158/0008-5472.CAN-16-3521. 28947416 10.1158/0008-5472.CAN-16-3521 PMC5690883

[52] Wu R-S, Hong J-J, Wu J-F, Yan S, Wu D, Liu N, et al. OVOL2 antagonizes TGF-β signaling to regulate epithelial to mesenchymal transition during mammary tumor metastasis. Oncotarget. 2017;8:39401–16. 10.18632/oncotarget.17031. 28455959 10.18632/oncotarget.17031 PMC5503621

[53] Wang G, Pan J, Zhang L, Wang C. Overexpression of grainyhead-like transcription factor 2 is associated with poor prognosis in human pancreatic carcinoma. Oncol Lett. 2019;17:1491–6. 10.3892/ol.2018.9741. 30675204 10.3892/ol.2018.9741 PMC6341798

[54] Jolly MK, Tripathi SC, Jia D, Mooney SM, Celiktas M, Hanash SM, et al. Stability of the hybrid epithelial/mesenchymal phenotype. Oncotarget. 2016;7:27067–84. 10.18632/oncotarget.8166. 27008704 10.18632/oncotarget.8166 PMC5053633

[55] Jia D, Jolly MK, Boareto M, Parsana P, Mooney SM, Pienta KJ, et al. OVOL guides the epithelial-hybrid-mesenchymal transition. Oncotarget. 2015;6:15436–48. 10.18632/oncotarget.3623. 25944618 10.18632/oncotarget.3623 PMC4558162

[56] Fischer KR, Durrans A, Lee S, Sheng J, Li F, Wong STC, et al. Epithelial-to-mesenchymal transition is not required for lung metastasis but contributes to chemoresistance. Nature. 2015;527:472–6. 10.1038/nature15748. 26560033 10.1038/nature15748 PMC4662610

[57] Rhim AD, Mirek ET, Aiello NM, Maitra A, Bailey JM, McAllister F, et al. EMT and dissemination precede pancreatic tumor formation. Cell. 2012;148:349–61. 10.1016/j.cell.2011.11.025. 22265420 10.1016/j.cell.2011.11.025 PMC3266542

[58] Zheng X, Carstens JL, Kim J, Scheible M, Kaye J, Sugimoto H, et al. Epithelial-to-mesenchymal transition is dispensable for metastasis but induces chemoresistance in pancreatic cancer. Nature. 2015;527:525–30. 10.1038/nature16064. 26560028 10.1038/nature16064 PMC4849281

[59] Fischer KR, Altorki NK, Mittal V, Gao D, Fischer, et al. reply Nat. 2017;547:E5–6. 10.1038/nature22817. 10.1038/nature22817 PMC6377064 28682327

[60] Chaffer CL, San Juan BP, Lim E, Weinberg RA. EMT, cell plasticity and metastasis. Cancer Metastasis Rev. 2016;35:645–54. 10.1007/s10555-016-9648-7. 27878502 10.1007/s10555-016-9648-7

[61] Valastyan S, Weinberg RA. Tumor metastasis: molecular insights and evolving paradigms. Cell. 2011;147:275–92. 10.1016/j.cell.2011.09.024. 22000009 10.1016/j.cell.2011.09.024 PMC3261217

[62] Di Franco S, Bianca P, Sardina DS, Turdo A, Gaggianesi M, Veschi V, et al. Adipose stem cell niche reprograms the colorectal cancer stem cell metastatic machinery. Nat Commun. 2021;12:5006. 10.1038/s41467-021-25333-9. 34408135 10.1038/s41467-021-25333-9 PMC8373975

[63] Mani SA, Guo W, Liao M-J, Eaton EN, Ayyanan A, Zhou AY, et al. The epithelial-mesenchymal transition generates cells with properties of stem cells. Cell. 2008;133:704–15. 10.1016/j.cell.2008.03.027. 18485877 10.1016/j.cell.2008.03.027 PMC2728032

[64] van der Zalm AP, Dings MPG, Manoukian P, Boersma H, Janssen R, Bailey P, et al. The pluripotency factor NANOG contributes to mesenchymal plasticity and is predictive for outcome in esophageal adenocarcinoma. Commun Med (Lond). 2024;4:89. 10.1038/s43856-024-00512-z. 38760583 10.1038/s43856-024-00512-z PMC11101480

[65] Subbalakshmi AR, Sahoo S, Biswas K, Jolly MK. A computational systems biology approach identifies SLUG as a mediator of partial epithelial-mesenchymal transition (EMT). Cells Tissues Organs. 2022;211:689–702. 10.1159/000512520. 33567424 10.1159/000512520

[66] Lu F, Li L, Wang L, Shu S, Gao J, Chang L, et al. Integrative stemness- and EMT-related gene signatures associated with prognosis and the immune microenvironment in lung adenocarcinoma. Discov Oncol. 2025;16:1139. 10.1007/s12672-025-02866-9. 40531415 10.1007/s12672-025-02866-9 PMC12177122

[67] Lei Z-N, Teng Q-X, Koya J, Liu Y, Chen Z, Zeng L, et al. The correlation between cancer stem cells and epithelial-mesenchymal transition: molecular mechanisms and significance in cancer theragnosis. Front Immunol. 2024;15:1417201. 10.3389/fimmu.2024.1417201. 39403386 10.3389/fimmu.2024.1417201 PMC11471544

[68] Li Z, Yang Z, Liu W, Zhu W, Yin L, Han Z, et al. Disheveled3 enhanced EMT and cancer stem-like cells properties via Wnt/β-catenin/c-Myc/SOX2 pathway in colorectal cancer. J Transl Med. 2023;21:302. 10.1186/s12967-023-04120-8. 37147666 10.1186/s12967-023-04120-8 PMC10161491

[69] Lan L, Li H, Zhang S, Garrone CM, Wu Y, Zaw Thin M, et al. A Jagged1-regulated hybrid-EMT state identifies pancreatic cancer stem cells. Cell Rep. 2026;45:116909. 10.1016/j.celrep.2025.116909. 41581147 10.1016/j.celrep.2025.116909

[70] Haerinck J, Goossens S, Berx G. The epithelial-mesenchymal plasticity landscape: principles of design and mechanisms of regulation. Nat Rev Genet. 2023;24:590–609. 10.1038/s41576-023-00601-0. 37169858 10.1038/s41576-023-00601-0

[71] Lee E, Wang J, Yumoto K, Jung Y, Cackowski FC, Decker AM et al. DNMT1 regulates epithelial-mesenchymal transition and cancer stem cells, which promotes prostate cancer metastasis. Neoplasia. New York, N.Y.; 2016;18:553–66. 10.1016/j.neo.2016.07.007 10.1016/j.neo.2016.07.007 PMC5031902 27659015

[72] Tam WL, Lu H, Buikhuisen J, Soh BS, Lim E, Reinhardt F, et al. Protein kinase C α is a central signaling node and therapeutic target for breast cancer stem cells. Cancer Cell. 2013;24:347–64. 10.1016/j.ccr.2013.08.005. 24029232 10.1016/j.ccr.2013.08.005 PMC4001722

[73] Yuan Y, Tang Y, Fang Z, Wen J, Wicha MS, Luo M. Long Non-Coding RNAs: Key regulators of tumor epithelial/mesenchymal plasticity and cancer stemness. Cells. 2025;14:227. 10.3390/cells14030227. 39937018 10.3390/cells14030227 PMC11817775

[74] Li J, Wang J, Chen Y, Li S, Jin M, Wang H, et al. LncRNA MALAT1 exerts oncogenic functions in lung adenocarcinoma by targeting miR-204. Am J Cancer Res. 2016;6:1099–107. 27294002 PMC4889723

[75] Sun R, Qin C, Jiang B, Fang S, Pan X, Peng L, et al. Down-regulation of MALAT1 inhibits cervical cancer cell invasion and metastasis by inhibition of epithelial-mesenchymal transition. Mol Biosyst. 2016;12:952–62. 10.1039/c5mb00685f. 26798987 10.1039/c5mb00685f

[76] Amicone L, Marchetti A, Cicchini C. The lncRNA HOTAIR: a pleiotropic regulator of epithelial cell plasticity. J Exp Clin Cancer Res. 2023;42:147. 10.1186/s13046-023-02725-x. 37308974 10.1186/s13046-023-02725-x PMC10262438

[77] Wei X, Ge Y, Zheng Y, Zhao S, Zhou Y, Chang Y et al. Hybrid EMT phenotype and cell membrane tension promote colorectal cancer resistance to ferroptosis. Adv Sci (Weinh). Weinheim, Baden-Wurttemberg, Germany; 2025;12:e2413882. 10.1002/advs.202413882 10.1002/advs.202413882 PMC12005738 39985376

[78] Chen L, Boleslaw Olszewski M, Kruithof-de Julio M, Snaar-Jagalska BE. Zebrafish microenvironment elevates EMT and CSC-like phenotype of engrafted prostate cancer cells. Cells. 2020;9:797. 10.3390/cells9040797. 10.3390/cells9040797 PMC7226630 32225005

[79] Chen X, Yang M, Yin J, Li P, Zeng S, Zheng G, et al. Tumor-associated macrophages promote epithelial-mesenchymal transition and the cancer stem cell properties in triple-negative breast cancer through CCL2/AKT/β-catenin signaling. Cell Commun Signal. 2022;20:92. 10.1186/s12964-022-00888-2. 35715860 10.1186/s12964-022-00888-2 PMC9205034

[80] Ko C-C, Yang P-M. Hypoxia-induced MIR31HG expression promotes partial EMT and basal-like phenotype in pancreatic ductal adenocarcinoma based on data mining and experimental analyses. J Transl Med. 2025;23:305. 10.1186/s12967-025-06292-x. 40065368 10.1186/s12967-025-06292-x PMC11895263

[81] Lundgren K, Nordenskjöld B, Landberg G, Hypoxia. Snail and incomplete epithelial-mesenchymal transition in breast cancer. Br J Cancer. 2009;101:1769–81. 10.1038/sj.bjc.6605369. 19844232 10.1038/sj.bjc.6605369 PMC2778529

[82] Pastushenko I, Mauri F, Song Y, de Cock F, Meeusen B, Swedlund B, et al. Fat1 deletion promotes hybrid EMT state, tumour stemness and metastasis. Nature. 2021;589:448–55. 10.1038/s41586-020-03046-1. 33328637 10.1038/s41586-020-03046-1 PMC7612440

[83] Bierie B, Pierce SE, Kroeger C, Stover DG, Pattabiraman DR, Thiru P, et al. Integrin-β4 identifies cancer stem cell-enriched populations of partially mesenchymal carcinoma cells. Proc Natl Acad Sci U S A. 2017;114:E2337–46. 10.1073/pnas.1618298114. 28270621 10.1073/pnas.1618298114 PMC5373369

[84] Kröger C, Afeyan A, Mraz J, Eaton EN, Reinhardt F, Khodor YL, et al. Acquisition of a hybrid E/M state is essential for tumorigenicity of basal breast cancer cells. Proc Natl Acad Sci U S A. 2019;116:7353–62. 10.1073/pnas.1812876116. 30910979 10.1073/pnas.1812876116 PMC6462070

[85] Correction for Kröger. Acquisition of a hybrid E/M state is essential for tumorigenicity of basal breast cancer cells. Proc Natl Acad Sci U S A. 2019;116:11553–4. 10.1073/pnas.1907473116. 30910979 10.1073/pnas.1812876116 PMC6462070

[86] Doran BR, Moffitt LR, Wilson AL, Stephens AN, Bilandzic M. Leader cells: invade and evade-the frontline of cancer progression. Int J Mol Sci. 2024;25:10554. 10.3390/ijms251910554. 39408880 10.3390/ijms251910554 PMC11476628

[87] Redfern AD, Spalding LJ, Thompson EW. The Kraken Wakes: induced EMT as a driver of tumour aggression and poor outcome. Clin Exp Metastasis. 2018;35:285–308. 10.1007/s10585-018-9906-x. 29948647 10.1007/s10585-018-9906-x

[88] Lu W, Kang Y. Epithelial-mesenchymal plasticity in cancer progression and metastasis. Dev Cell. 2019;49:361–74. 10.1016/j.devcel.2019.04.010. 31063755 10.1016/j.devcel.2019.04.010 PMC6506183

[89] Youssef KK, Narwade N, Arcas A, Marquez-Galera A, Jiménez-Castaño R, Lopez-Blau C, et al. Two distinct epithelial-to-mesenchymal transition programs control invasion and inflammation in segregated tumor cell populations. Nat Cancer. 2024;5:1660–80. 10.1038/s43018-024-00839-5. 39414946 10.1038/s43018-024-00839-5 PMC11584407

[90] Kuburich NA, den Hollander P, Castaneda M, Pietilä M, Tang X, Batra H, et al. Stabilizing vimentin phosphorylation inhibits stem-like cell properties and metastasis of hybrid epithelial/mesenchymal carcinomas. Cell Rep. 2023;42:113470. 10.1016/j.celrep.2023.113470. 37979166 10.1016/j.celrep.2023.113470 PMC11062250

[91] Mao Y, Xia Z, Xia W, Jiang P. Metabolic reprogramming, sensing, and cancer therapy. Cell Rep. 2024;43:115064. 10.1016/j.celrep.2024.115064. 39671294 10.1016/j.celrep.2024.115064

[92] Liaghat M, Ferdousmakan S, Mortazavi SH, Yahyazadeh S, Irani A, Banihashemi S, et al. The impact of epithelial-mesenchymal transition (EMT) induced by metabolic processes and intracellular signaling pathways on chemo-resistance, metastasis, and recurrence in solid tumors. Cell Commun Signal. 2024;22:575. 10.1186/s12964-024-01957-4. 39623377 10.1186/s12964-024-01957-4 PMC11610171

[93] Xie Y, Ma S, Tong M. Metabolic plasticity of cancer stem cells in response to microenvironmental cues. Cancers (Basel). 2022;14:5345. 10.3390/cancers14215345. 36358763 10.3390/cancers14215345 PMC9657081

[94] Tan Y, Li J, Zhao G, Huang K-C, Cardenas H, Wang Y, et al. Metabolic reprogramming from glycolysis to fatty acid uptake and beta-oxidation in platinum-resistant cancer cells. Nat Commun. 2022;13:4554. 10.1038/s41467-022-32101-w. 35931676 10.1038/s41467-022-32101-w PMC9356138

[95] Liu S, Zhang X, Wang W, Li X, Sun X, Zhao Y, et al. Metabolic reprogramming and therapeutic resistance in primary and metastatic breast cancer. Mol Cancer. 2024;23:261. 10.1186/s12943-024-02165-x. 39574178 10.1186/s12943-024-02165-x PMC11580516

[96] Jia D, Park JH, Kaur H, Jung KH, Yang S, Tripathi S, et al. Towards decoding the coupled decision-making of metabolism and epithelial-to-mesenchymal transition in cancer. Br J Cancer. 2021;124:1902–11. 10.1038/s41416-021-01385-y. 33859341 10.1038/s41416-021-01385-y PMC8184790

[97] Zu XL, Guppy M. Cancer metabolism: facts, fantasy, and fiction. Biochem Biophys Res Commun. 2004;313:459–65. 10.1016/j.bbrc.2003.11.136. 14697210 10.1016/j.bbrc.2003.11.136

[98] Krapf SA, Lund J, Lundkvist M, Dale MG, Nyman TA, Thoresen GH, et al. Pancreatic cancer cells show lower oleic acid oxidation and their conditioned medium inhibits oleic acid oxidation in human myotubes. Pancreatology. 2020;20:676–82. 10.1016/j.pan.2020.04.014. 32360002 10.1016/j.pan.2020.04.014

[99] Galbraith M, Levine H, Onuchic JN, Jia D. Decoding the coupled decision-making of the epithelial-mesenchymal transition and metabolic reprogramming in cancer. iScience. 2023;26:105719. 10.1016/j.isci.2022.105719. 36582834 10.1016/j.isci.2022.105719 PMC9792913

[100] Jia D, Lu M, Jung KH, Park JH, Yu L, Onuchic JN, et al. Elucidating cancer metabolic plasticity by coupling gene regulation with metabolic pathways. Proc Natl Acad Sci USA. 2019;116:3909–18. 10.1073/pnas.1816391116. 30733294 10.1073/pnas.1816391116 PMC6397570

[101] Yu L, Lu M, Jia D, Ma J, Ben-Jacob E, Levine H, et al. Modeling the genetic regulation of cancer metabolism: interplay between glycolysis and oxidative phosphorylation. Cancer Res. 2017;77:1564–74. 10.1158/0008-5472.CAN-16-2074. 28202516 10.1158/0008-5472.CAN-16-2074 PMC5380541

[102] Luo M, Shang L, Brooks MD, Jiagge E, Zhu Y, Buschhaus JM, et al. Targeting breast cancer stem cell state equilibrium through modulation of redox signaling. Cell Metab. 2018;28:69–e866. 10.1016/j.cmet.2018.06.006. 29972798 10.1016/j.cmet.2018.06.006 PMC6037414

[103] Colacino JA, Azizi E, Brooks MD, Harouaka R, Fouladdel S, McDermott SP, et al. Heterogeneity of human breast stem and progenitor cells as revealed by transcriptional profiling. Stem Cell Rep. 2018;10:1596–609. 10.1016/j.stemcr.2018.03.001. 10.1016/j.stemcr.2018.03.001 PMC5995162 29606612

[104] Ren Z, Dharmaratne M, Liang H, Benard O, Morales-Gallego M, Suyama K, et al. Redox signalling regulates breast cancer metastasis via phenotypic and metabolic reprogramming due to p63 activation by HIF1α. Br J Cancer. 2024;130:908–24. 10.1038/s41416-023-02522-5. 38238426 10.1038/s41416-023-02522-5 PMC10951347

[105] LeBleu VS, O’Connell JT, Gonzalez Herrera KN, Wikman H, Pantel K, Haigis MC, et al. PGC-1α mediates mitochondrial biogenesis and oxidative phosphorylation in cancer cells to promote metastasis. Nat Cell Biol. 2014;16:992–1003. 10.1038/ncb3039. 25241037 10.1038/ncb3039 PMC4369153

[106] Fernando W, Cruickshank BM, Arun RP, MacLean MR, Cahill HF, Morales-Quintanilla F, et al. ALDH1A3 is the switch that determines the balance of ALDH + and CD24-CD44 + cancer stem cells, EMT-MET, and glucose metabolism in breast cancer. Oncogene. 2024;43:3151–69. 10.1038/s41388-024-03156-4. 39251846 10.1038/s41388-024-03156-4 PMC11493680

[107] Sun K, Ling H, Peng F, Yang T, Hao Y, Bai Q, et al. FADS3 fuels CcRCC progression via lipid-droplet/TGF-β receptors axis bridging metabolic reprogramming and epithelial plasticity. Int J Surg. 2025. 10.1097/JS9.0000000000004094. cited 2026 Jan 28. 41314810 10.1097/JS9.0000000000004094

[108] Cai H, Li J, Zhang Y, Liao Y, Zhu Y, Wang C, et al. LDHA Promotes oral squamous cell carcinoma progression through facilitating glycolysis and epithelial-mesenchymal transition. Front Oncol. 2019;9:1446. 10.3389/fonc.2019.01446. 31921691 10.3389/fonc.2019.01446 PMC6930919

[109] Corbet C, Bastien E, Santiago de Jesus JP, Dierge E, Martherus R, Vander Linden C, et al. TGFβ2-induced formation of lipid droplets supports acidosis-driven EMT and the metastatic spreading of cancer cells. Nat Commun. 2020;11:454. 10.1038/s41467-019-14262-3. 31974393 10.1038/s41467-019-14262-3 PMC6978517

[110] Khajah MA, Khushaish S, Luqmani YA. Lactate dehydrogenase a or b knockdown reduces lactate production and inhibits breast cancer cell motility in vitro. Front Pharmacol. 2021;12:747001. 10.3389/fphar.2021.747001. 34744727 10.3389/fphar.2021.747001 PMC8564068

[111] Weihua Z, Tsan R, Huang W-C, Wu Q, Chiu C-H, Fidler IJ, et al. Survival of cancer cells is maintained by EGFR independent of its kinase activity. Cancer Cell. 2008;13:385–93. 10.1016/j.ccr.2008.03.015. 18455122 10.1016/j.ccr.2008.03.015 PMC2413063

[112] Jeon S-M. Regulation and function of AMPK in physiology and diseases. Exp Mol Med. 2016;48:e245. 10.1038/emm.2016.81. 27416781 10.1038/emm.2016.81 PMC4973318

[113] Ulasov AV, Rosenkranz AA, Georgiev GP, Sobolev AS. Nrf2/Keap1/ARE signaling: towards specific regulation. Life Sci. 2022;291:120111. 10.1016/j.lfs.2021.120111. 34732330 10.1016/j.lfs.2021.120111 PMC8557391

[114] Song M-Y, Lee D-Y, Chun K-S, Kim E-H. The Role of NRF2/KEAP1 signaling pathway in cancer metabolism. Int J Mol Sci. 2021;22:4376. 10.3390/ijms22094376. 33922165 10.3390/ijms22094376 PMC8122702

[115] Riz I, Hawley TS, Marsal JW, Hawley RG. Noncanonical SQSTM1/p62-Nrf2 pathway activation mediates proteasome inhibitor resistance in multiple myeloma cells via redox, metabolic and translational reprogramming. Oncotarget. 2016;7:66360–85. 10.18632/oncotarget.11960. 27626179 10.18632/oncotarget.11960 PMC5340085

[116] Kay HY, Kim WD, Hwang SJ, Choi H-S, Gilroy RK, Wan Y-JY, et al. Nrf2 inhibits LXRα-dependent hepatic lipogenesis by competing with FXR for acetylase binding. Antioxid Redox Signal. 2011;15:2135–46. 10.1089/ars.2010.3834. 21504366 10.1089/ars.2010.3834 PMC6468953

[117] Slocum SL, Skoko JJ, Wakabayashi N, Aja S, Yamamoto M, Kensler TW, et al. Keap1/Nrf2 pathway activation leads to a repressed hepatic gluconeogenic and lipogenic program in mice on a high-fat diet. Arch Biochem Biophys. 2016;591:57–65. 10.1016/j.abb.2015.11.040. 26701603 10.1016/j.abb.2015.11.040 PMC4747866

[118] Matsumoto A, Inoko A, Tanaka T, Konishi G-I, Hosoda W, Kojima T, et al. Chemotherapy resistance due to epithelial-to-mesenchymal transition is caused by abnormal lipid metabolic balance. eLife. 2026;13:RP104374. 10.7554/eLife.104374. 41521893 10.7554/eLife.104374 PMC12795503

[119] Daniel Y, Lelou E, Aninat C, Corlu A, Cabillic F. Interplay between metabolism reprogramming and epithelial-to-mesenchymal transition in cancer stem cells. Cancers (Basel). 2021;13:1973. 10.3390/cancers13081973. 33923958 10.3390/cancers13081973 PMC8072988

[120] Santos CR, Schulze A. Lipid metabolism in cancer. FEBS J. 2012;279:2610–23. 10.1111/j.1742-4658.2012.08644.x. 22621751 10.1111/j.1742-4658.2012.08644.x

[121] Wen J, Min X, Shen M, Hua Q, Han Y, Zhao L, et al. ACLY facilitates colon cancer cell metastasis by CTNNB1. J Exp Clin Cancer Res. 2019;38:401. 10.1186/s13046-019-1391-9. 31511060 10.1186/s13046-019-1391-9 PMC6740040

[122] Migita T, Narita T, Nomura K, Miyagi E, Inazuka F, Matsuura M, et al. ATP citrate lyase: activation and therapeutic implications in non-small cell lung cancer. Cancer Res. 2008;68:8547–54. 10.1158/0008-5472.CAN-08-1235. 18922930 10.1158/0008-5472.CAN-08-1235

[123] Hanai J, Doro N, Sasaki AT, Kobayashi S, Cantley LC, Seth P, et al. Inhibition of lung cancer growth: ATP citrate lyase knockdown and statin treatment leads to dual blockade of mitogen-activated protein kinase (MAPK) and phosphatidylinositol-3-kinase (PI3K)/AKT pathways. J Cell Physiol. 2012;227:1709–20. 10.1002/jcp.22895. 21688263 10.1002/jcp.22895 PMC3407542

[124] Shuvalov O, Daks A, Fedorova O, Petukhov A, Barlev N. Linking metabolic reprogramming, plasticity and tumor progression. Cancers (Basel). 2021;13:762. 10.3390/cancers13040762. 33673109 10.3390/cancers13040762 PMC7917602

[125] Lai X, Li Q, Wu F, Lin J, Chen J, Zheng H, et al. Epithelial-mesenchymal transition and metabolic switching in cancer: lessons from somatic cell reprogramming. Front Cell Dev Biol. 2020;8:760. 10.3389/fcell.2020.00760. 32850862 10.3389/fcell.2020.00760 PMC7423833

[126] Luo J, Hong Y, Lu Y, Qiu S, Chaganty BKR, Zhang L, et al. Acetyl-CoA carboxylase rewires cancer metabolism to allow cancer cells to survive inhibition of the Warburg effect by cetuximab. Cancer Lett. 2017;384:39–49. 10.1016/j.canlet.2016.09.020. 27693630 10.1016/j.canlet.2016.09.020 PMC5110372

[127] Xu Y, Huang J, Xin W, Chen L, Zhao X, Lv Z, et al. Lipid accumulation is ahead of epithelial-to-mesenchymal transition and therapeutic intervention by acetyl-CoA carboxylase 2 silence in diabetic nephropathy. Metabolism. 2014;63:716–26. 10.1016/j.metabol.2014.02.010. 24650564 10.1016/j.metabol.2014.02.010

[128] Rios Garcia M, Steinbauer B, Srivastava K, Singhal M, Mattijssen F, Maida A, et al. Acetyl-CoA Carboxylase 1-dependent protein acetylation controls breast cancer metastasis and recurrence. Cell Metab. 2017;26:842–e8555. 10.1016/j.cmet.2017.09.018. 29056512 10.1016/j.cmet.2017.09.018

[129] Liu H, Liu J-Y, Wu X, Zhang J-T. Biochemistry, molecular biology, and pharmacology of fatty acid synthase, an emerging therapeutic target and diagnosis/prognosis marker. Int J Biochem Mol Biol. 2010;1:69–89. 20706604 PMC2919769

[130] Yasumoto Y, Miyazaki H, Vaidyan LK, Kagawa Y, Ebrahimi M, Yamamoto Y, et al. Inhibition of fatty acid synthase decreases expression of stemness markers in glioma stem cells. PLoS ONE. 2016;11:e0147717. 10.1371/journal.pone.0147717. 26808816 10.1371/journal.pone.0147717 PMC4726602

[131] Yang L, Zhang F, Wang X, Tsai Y, Chuang K-H, Keng PC, et al. A FASN-TGF-β1-FASN regulatory loop contributes to high EMT/metastatic potential of cisplatin-resistant non-small cell lung cancer. Oncotarget. 2016;7:55543–54. 10.18632/oncotarget.10837. 27765901 10.18632/oncotarget.10837 PMC5342435

[132] Singh R, Yadav V, Kumar S, Saini N. MicroRNA-195 inhibits proliferation, invasion and metastasis in breast cancer cells by targeting FASN, HMGCR, ACACA and CYP27B1. Sci Rep. 2015;5:17454. 10.1038/srep17454. 26632252 10.1038/srep17454 PMC4668367

[133] Chen T, Zhou L, Li H, Tian Y, Li J, Dong L, et al. Fatty acid synthase affects expression of ErbB receptors in epithelial to mesenchymal transition of breast cancer cells and invasive ductal carcinoma. Oncol Lett. 2017;14:5934–46. 10.3892/ol.2017.6954. 29113229 10.3892/ol.2017.6954 PMC5661422

[134] Ran H, Zhu Y, Deng R, Zhang Q, Liu X, Feng M, et al. Stearoyl-CoA desaturase-1 promotes colorectal cancer metastasis in response to glucose by suppressing PTEN. J Exp Clin Cancer Res. 2018;37:54. 10.1186/s13046-018-0711-9. 29530061 10.1186/s13046-018-0711-9 PMC5848567

[135] Mauvoisin D, Charfi C, Lounis AM, Rassart E, Mounier C. Decreasing stearoyl-CoA desaturase-1 expression inhibits β-catenin signaling in breast cancer cells. Cancer Sci. 2013;104:36–42. 10.1111/cas.12032. 23013158 10.1111/cas.12032 PMC7657162

[136] Sánchez-Martínez R, Cruz-Gil S, Gómez de Cedrón M, Álvarez-Fernández M, Vargas T, Molina S, et al. A link between lipid metabolism and epithelial-mesenchymal transition provides a target for colon cancer therapy. Oncotarget. 2015;6:38719–36. 10.18632/oncotarget.5340. 26451612 10.18632/oncotarget.5340 PMC4770732

[137] Rossi Sebastiano M, Konstantinidou G. Targeting Long Chain Acyl-CoA synthetases for cancer therapy. Int J Mol Sci. 2019;20:3624. 10.3390/ijms20153624. 31344914 10.3390/ijms20153624 PMC6696099

[138] Sánchez-Martínez R, Cruz-Gil S, García-Álvarez MS, Reglero G, Ramírez de Molina A. Complementary ACSL isoforms contribute to a non-Warburg advantageous energetic status characterizing invasive colon cancer cells. Sci Rep. 2017;7:11143. 10.1038/s41598-017-11612-3. 28894242 10.1038/s41598-017-11612-3 PMC5593891

[139] Pouyafar A, Heydarabad MZ, Abdolalizadeh J, Zade JA, Rahbarghazi R, Talebi M. Modulation of lipolysis and glycolysis pathways in cancer stem cells changed multipotentiality and differentiation capacity toward endothelial lineage. Cell Biosci. 2019;9:30. 10.1186/s13578-019-0293-z. 30962872 10.1186/s13578-019-0293-z PMC6437852

[140] Pouyafar A, Heydarabad MZ, Abdolalizadeh J, Rahbarghazi R, Talebi M. Correction to: Modulation of lipolysis and glycolysis pathways in cancer stem cells changed multipotentiality and differentiation capacity toward endothelial lineage. Cell Biosci. 2019;9:37. 10.1186/s13578-019-0301-3. 31168353 10.1186/s13578-019-0301-3 PMC6509775

[141] Morandi A, Taddei ML, Chiarugi P, Giannoni E. Targeting the metabolic reprogramming that controls epithelial-to-mesenchymal transition in aggressive tumors. Front Oncol. 2017;7:40. 10.3389/fonc.2017.00040. 28352611 10.3389/fonc.2017.00040 PMC5348536

[142] Lei Y, Cai S, Zhang J-K, Ding S-Q, Zhang Z-H, Zhang C-D, et al. The role and mechanism of fatty acid oxidation in cancer drug resistance. Cell Death Discov. 2025;11:277. 10.1038/s41420-025-02554-1. 40514365 10.1038/s41420-025-02554-1 PMC12166077

[143] Tian T, Lu Y, Lin J, Chen M, Qiu H, Zhu W, et al. CPT1A promotes anoikis resistance in esophageal squamous cell carcinoma via redox homeostasis. Redox Biol. 2022;58:102544. 10.1016/j.redox.2022.102544. 36427397 10.1016/j.redox.2022.102544 PMC9692043

[144] Nath A, Li I, Roberts LR, Chan C. Elevated free fatty acid uptake via CD36 promotes epithelial-mesenchymal transition in hepatocellular carcinoma. Sci Rep. 2015;5:14752. 10.1038/srep14752. 26424075 10.1038/srep14752 PMC4589791

[145] Deng M, Cai X, Long L, Xie L, Ma H, Zhou Y, et al. CD36 promotes the epithelial-mesenchymal transition and metastasis in cervical cancer by interacting with TGF-β. J Transl Med. 2019;17:352. 10.1186/s12967-019-2098-6. 31655604 10.1186/s12967-019-2098-6 PMC6815430

[146] Lee H, Park S, Lee J, Lee C, Kang H, Kang J, et al. Lipid metabolism in cancer stem cells: reprogramming, mechanisms, crosstalk, and therapeutic approaches. Cell Oncol (Dordr) Dordrecht Neth. 2025;48:1181–201. 10.1007/s13402-025-01081-6. 10.1007/s13402-025-01081-6 PMC12528255 40586825

[147] Oginuma M, Harima Y, Tarazona OA, Diaz-Cuadros M, Michaut A, Ishitani T, et al. Intracellular pH controls WNT downstream of glycolysis in amniote embryos. Nature. 2020;584:98–101. 10.1038/s41586-020-2428-0. 32581357 10.1038/s41586-020-2428-0 PMC8278564

[148] Hao Y, Baker D, Ten Dijke P. TGF-β-Mediated epithelial-mesenchymal transition and cancer metastasis. Int J Mol Sci. 2019;20:2767. 10.3390/ijms20112767. 31195692 10.3390/ijms20112767 PMC6600375

[149] Xu J, Lamouille S, Derynck R. TGF-beta-induced epithelial to mesenchymal transition. Cell Res. 2009;19:156–72. 10.1038/cr.2009.5. 19153598 10.1038/cr.2009.5 PMC4720263

[150] Liu R-M, Desai LP. Reciprocal regulation of TGF-β and reactive oxygen species: a perverse cycle for fibrosis. Redox Biol. 2015;6:565–77. 10.1016/j.redox.2015.09.009. 26496488 10.1016/j.redox.2015.09.009 PMC4625010

[151] Jiang L, Xiao L, Sugiura H, Huang X, Ali A, Kuro-o M, et al. Metabolic reprogramming during TGFβ1-induced epithelial-to-mesenchymal transition. Oncogene. 2015;34:3908–16. 10.1038/onc.2014.321. 25284588 10.1038/onc.2014.321 PMC4387121

[152] Fedele M, Sgarra R, Battista S, Cerchia L, Manfioletti G. The epithelial-mesenchymal transition at the crossroads between metabolism and tumor progression. Int J Mol Sci. 2022;23:800. 10.3390/ijms23020800. 35054987 10.3390/ijms23020800 PMC8776206

[153] Dong C, Yuan T, Wu Y, Wang Y, Fan TWM, Miriyala S, et al. Loss of FBP1 by Snail-mediated repression provides metabolic advantages in basal-like breast cancer. Cancer Cell. 2013;23:316–31. 10.1016/j.ccr.2013.01.022. 23453623 10.1016/j.ccr.2013.01.022 PMC3703516

[154] Masin M, Vazquez J, Rossi S, Groeneveld S, Samson N, Schwalie PC, et al. GLUT3 is induced during epithelial-mesenchymal transition and promotes tumor cell proliferation in non-small cell lung cancer. Cancer Metab. 2014;2:11. 10.1186/2049-3002-2-11. 25097756 10.1186/2049-3002-2-11 PMC4122054

[155] Ha T-K, Her N-G, Lee M-G, Ryu B-K, Lee J-H, Han J, et al. Caveolin-1 increases aerobic glycolysis in colorectal cancers by stimulating HMGA1-mediated GLUT3 transcription. Cancer Res. 2012;72:4097–109. 10.1158/0008-5472.CAN-12-0448. 22706202 10.1158/0008-5472.CAN-12-0448

[156] Sun Y, Daemen A, Hatzivassiliou G, Arnott D, Wilson C, Zhuang G, et al. Metabolic and transcriptional profiling reveals pyruvate dehydrogenase kinase 4 as a mediator of epithelial-mesenchymal transition and drug resistance in tumor cells. Cancer Metab. 2014;2:20. 10.1186/2049-3002-2-20. 25379179 10.1186/2049-3002-2-20 PMC4221711

[157] Hamabe A, Konno M, Tanuma N, Shima H, Tsunekuni K, Kawamoto K, et al. Role of pyruvate kinase M2 in transcriptional regulation leading to epithelial-mesenchymal transition. Proc Natl Acad Sci U S A. 2014;111:15526–31. 10.1073/pnas.1407717111. 25313085 10.1073/pnas.1407717111 PMC4217454

[158] Rodríguez-García A, Samsó P, Fontova P, Simon-Molas H, Manzano A, Castaño E, et al. TGF-β1 targets Smad, p38 MAPK, and PI3K/Akt signaling pathways to induce PFKFB3 gene expression and glycolysis in glioblastoma cells. FEBS J. 2017;284:3437–54. 10.1111/febs.14201. 28834297 10.1111/febs.14201

[159] Yalcin A, Solakoglu TH, Ozcan SC, Guzel S, Peker S, Celikler S, et al. 6-phosphofructo-2-kinase/fructose 2,6-bisphosphatase-3 is required for transforming growth factor β1-enhanced invasion of Panc1 cells in vitro. Biochem Biophys Res Commun. 2017;484:687–93. 10.1016/j.bbrc.2017.01.178. 28161638 10.1016/j.bbrc.2017.01.178

[160] Bhowmick R, Campit S, Katkam SK, Keshamouni VG, Chandrasekaran S. Genome-scale modeling identifies dynamic metabolic vulnerabilities during the epithelial to mesenchymal transition. Commun Biol. 2024;7:1704. 10.1038/s42003-024-07408-7. 39730911 10.1038/s42003-024-07408-7 PMC11681178

[161] Kim NH, Cha YH, Lee J, Lee S-H, Yang JH, Yun JS, et al. Snail reprograms glucose metabolism by repressing phosphofructokinase PFKP allowing cancer cell survival under metabolic stress. Nat Commun. 2017;8:14374. 10.1038/ncomms14374. 28176759 10.1038/ncomms14374 PMC5309788

[162] Dalmau N, Jaumot J, Tauler R, Bedia C. Epithelial-to-mesenchymal transition involves triacylglycerol accumulation in DU145 prostate cancer cells. Mol Biosyst. 2015;11:3397–406. 10.1039/c5mb00413f. 26474270 10.1039/c5mb00413f

[163] Shaul YD, Freinkman E, Comb WC, Cantor JR, Tam WL, Thiru P, et al. Dihydropyrimidine accumulation is required for the epithelial-mesenchymal transition. Cell. 2014;158:1094–109. 10.1016/j.cell.2014.07.032. 25171410 10.1016/j.cell.2014.07.032 PMC4250222

[164] Ahmad A, Aboukameel A, Kong D, Wang Z, Sethi S, Chen W, et al. Phosphoglucose isomerase/autocrine motility factor mediates epithelial-mesenchymal transition regulated by miR-200 in breast cancer cells. Cancer Res. 2011;71:3400–9. 10.1158/0008-5472.CAN-10-0965. 21389093 10.1158/0008-5472.CAN-10-0965 PMC3085607

[165] Du S, Guan Z, Hao L, Song Y, Wang L, Gong L, et al. Fructose-bisphosphate aldolase a is a potential metastasis-associated marker of lung squamous cell carcinoma and promotes lung cell tumorigenesis and migration. PLoS ONE. 2014;9:e85804. 10.1371/journal.pone.0085804. 24465716 10.1371/journal.pone.0085804 PMC3900443

[166] Liu K, Tang Z, Huang A, Chen P, Liu P, Yang J, et al. Glyceraldehyde-3-phosphate dehydrogenase promotes cancer growth and metastasis through upregulation of SNAIL expression. Int J Oncol. 2017;50:252–62. 10.3892/ijo.2016.3774. 27878251 10.3892/ijo.2016.3774

[167] Jiang F, Ma S, Xue Y, Hou J, Zhang Y. LDH-A promotes malignant progression via activation of epithelial-to-mesenchymal transition and conferring stemness in muscle-invasive bladder cancer. Biochem Biophys Res Commun. 2016;469:985–92. 10.1016/j.bbrc.2015.12.078. 26721441 10.1016/j.bbrc.2015.12.078

[168] Wu J, Chen J, Xi Y, Wang F, Sha H, Luo L, et al. High glucose induces epithelial-mesenchymal transition and results in the migration and invasion of colorectal cancer cells. Exp Ther Med. 2018;16:222–30. 10.3892/etm.2018.6189. 29896243 10.3892/etm.2018.6189 PMC5995072

[169] Li S, Zhu H, Chen H, Xia J, Zhang F, Xu R, et al. Glucose promotes epithelial-mesenchymal transitions in bladder cancer by regulating the functions of YAP1 and TAZ. J Cell Mol Med. 2020;24:10391–401. 10.1111/jcmm.15653. 32678516 10.1111/jcmm.15653 PMC7521329

[170] Li S, Ruan B, Wang Z, Xia J, Lin Q, Xu R, et al. Glucose dysregulation promotes oncogenesis in human bladder cancer by regulating autophagy and YAP1/TAZ expression. J Cell Mol Med. 2023;27:3744–59. 10.1111/jcmm.17943. 37665055 10.1111/jcmm.17943 PMC10718143

[171] Sciacovelli M, Gonçalves E, Johnson TI, Zecchini VR, da Costa ASH, Gaude E, et al. Fumarate is an epigenetic modifier that elicits epithelial-to-mesenchymal transition. Nature. 2016;537:544–7. 10.1038/nature19353. 27580029 10.1038/nature19353 PMC5136292

[172] Sciacovelli M, Gonçalves E, Johnson TI, Zecchini VR, da Costa ASH, Gaude E, et al. Corrigendum: Fumarate is an epigenetic modifier that elicits epithelial-to-mesenchymal transition. Nature. 2016;540:150. 10.1038/nature20144. 27760119 10.1038/nature20144

[173] He X, Yan B, Liu S, Jia J, Lai W, Xin X, et al. Chromatin remodeling factor lsh drives cancer progression by suppressing the activity of fumarate hydratase. Cancer Res. 2016;76:5743–55. 10.1158/0008-5472.CAN-16-0268. 27302170 10.1158/0008-5472.CAN-16-0268 PMC7821962

[174] He X, Yan B, Liu S, Jia J, Lai W, Xin X, et al. Correction: chromatin remodeling factor lsh drives cancer progression by suppressing the activity of fumarate hydratase. Cancer Res. 2022;82:1435. 10.1158/0008-5472.CAN-22-0457. 35373294 10.1158/0008-5472.CAN-22-0457

[175] Wang H, Chen Y, Wu G. SDHB deficiency promotes TGFβ-mediated invasion and metastasis of colorectal cancer through transcriptional repression complex SNAIL1-SMAD3/4. Transl Oncol. 2016;9:512–20. 10.1016/j.tranon.2016.09.009. 27816688 10.1016/j.tranon.2016.09.009 PMC5097976

[176] Aspuria P-JP, Lunt SY, Väremo L, Vergnes L, Gozo M, Beach JA, et al. Succinate dehydrogenase inhibition leads to epithelial-mesenchymal transition and reprogrammed carbon metabolism. Cancer Metab. 2014;2:21. 10.1186/2049-3002-2-21. 25671108 10.1186/2049-3002-2-21 PMC4322794

[177] Liu J, Gao L, Zhang H, Wang D, Wang M, Zhu J, et al. Succinate dehydrogenase 5 (SDH5) regulates glycogen synthase kinase 3β-β-catenin-mediated lung cancer metastasis. J Biol Chem. 2013;288:29965–73. 10.1074/jbc.M113.450106. 23983127 10.1074/jbc.M113.450106 PMC3795294

[178] Grassian AR, Lin F, Barrett R, Liu Y, Jiang W, Korpal M, et al. Isocitrate dehydrogenase (IDH) mutations promote a reversible ZEB1/microRNA (miR)-200-dependent epithelial-mesenchymal transition (EMT). J Biol Chem. 2012;287:42180–94. 10.1074/jbc.M112.417832. 23038259 10.1074/jbc.M112.417832 PMC3516763

[179] Chen J, Cui L, Lu S, Xu S. Amino acid metabolism in tumor biology and therapy. Cell Death Dis. 2024;15:42. 10.1038/s41419-024-06435-w. 38218942 10.1038/s41419-024-06435-w PMC10787762

[180] Lee SY, Jeon HM, Ju MK, Jeong EK, Kim CH, Park HG, et al. Dlx-2 and glutaminase upregulate epithelial-mesenchymal transition and glycolytic switch. Oncotarget. 2016;7:7925–39. 10.18632/oncotarget.6879. 26771232 10.18632/oncotarget.6879 PMC4884964

[181] Ramirez-Peña E, Arnold J, Shivakumar V, Joseph R, Vidhya Vijay G, den Hollander P, et al. The epithelial to mesenchymal transition promotes glutamine independence by suppressing GLS2 expression. Cancers (Basel). 2019;11:1610. 10.3390/cancers11101610. 31652551 10.3390/cancers11101610 PMC6826439

[182] Dias MM, Adamoski D, Dos Reis LM, Ascenção CFR, de Oliveira KRS, Mafra ACP, et al. GLS2 is protumorigenic in breast cancers. Oncogene. 2020;39:690–702. 10.1038/s41388-019-1007-z. 31541193 10.1038/s41388-019-1007-z

[183] Liu G, Zhu J, Yu M, Cai C, Zhou Y, Yu M, et al. Glutamate dehydrogenase is a novel prognostic marker and predicts metastases in colorectal cancer patients. J Transl Med. 2015;13:144. 10.1186/s12967-015-0500-6. 25947346 10.1186/s12967-015-0500-6 PMC4490642

[184] Al-khreisat MJ, Abdulsahib WK, Jasim IK, Malathi H, Nayak PP, Anand DA, et al. Targeting cancer stem cell plasticity and tumor microenvironment crosstalk: a comprehensive review. Discov Onc. 2025;17:145. 10.1007/s12672-025-04297-y. 10.1007/s12672-025-04297-y PMC12834896 41422192

[185] De Visser KE, Joyce JA. The evolving tumor microenvironment: from cancer initiation to metastatic outgrowth. Cancer Cell. 2023;41:374–403. 10.1016/j.ccell.2023.02.016. 36917948 10.1016/j.ccell.2023.02.016

[186] Junk DJ, Cipriano R, Bryson BL, Gilmore HL, Jackson MW. Tumor microenvironmental signaling elicits epithelial-mesenchymal plasticity through cooperation with transforming genetic events. Volume 15. New York, N: Neoplasia; 2013. pp. 1100–9. 10.1593/neo.131114. 10.1593/neo.131114 PMC3769888 24027434

[187] Fiori ME, Di Franco S, Villanova L, Bianca P, Stassi G, De Maria R. Cancer-associated fibroblasts as abettors of tumor progression at the crossroads of EMT and therapy resistance. Mol Cancer. 2019;18:70. 10.1186/s12943-019-0994-2. 30927908 10.1186/s12943-019-0994-2 PMC6441236

[188] Szabo PM, Vajdi A, Kumar N, Tolstorukov MY, Chen BJ, Edwards R, et al. Cancer-associated fibroblasts are the main contributors to epithelial-to-mesenchymal signatures in the tumor microenvironment. Sci Rep. 2023;13:3051. 10.1038/s41598-023-28480-9. 36810872 10.1038/s41598-023-28480-9 PMC9944255

[189] Fang JS, Hultgren NW, Hughes CCW. Regulation of partial and reversible endothelial-to-mesenchymal transition in angiogenesis. Front Cell Dev Biol. 2021;9:702021. 10.3389/fcell.2021.702021. 34692672 10.3389/fcell.2021.702021 PMC8529039

[190] Wu X, Que H, Li Q, Wei X. Wnt/β-catenin mediated signaling pathways in cancer: recent advances, and applications in cancer therapy. Mol Cancer. 2025;24:171. 10.1186/s12943-025-02363-1. 40495229 10.1186/s12943-025-02363-1 PMC12150590

[191] Kim R, Chang W. Endothelial-to-mesenchymal transition in health and disease: molecular insights and therapeutic implications. IJMS. 2025;26:11724. 10.3390/ijms262311724. 41373869 10.3390/ijms262311724 PMC12693439

[192] Sigurdsson V, Hilmarsdottir B, Sigmundsdottir H, Fridriksdottir AJR, Ringnér M, Villadsen R et al. A <>Swarbrick editor 2011 endothelial induced EMT in breast epithelial cells with stem cell properties. PLoS ONE 6 e23833 10.1371/journal.pone.0023833. 21915264 10.1371/journal.pone.0023833 PMC3167828

[193] Yan T, Shi J. Angiogenesis and EMT regulators in the tumor microenvironment in lung cancer and immunotherapy. Front Immunol. 2024;15:1509195. 10.3389/fimmu.2024.1509195. 39737184 10.3389/fimmu.2024.1509195 PMC11682976

[194] Yang Y, Andersson P, Hosaka K, Zhang Y, Cao R, Iwamoto H, et al. The PDGF-BB-SOX7 axis-modulated IL-33 in pericytes and stromal cells promotes metastasis through tumour-associated macrophages. Nat Commun. 2016;7:11385. 10.1038/ncomms11385. 27150562 10.1038/ncomms11385 PMC4859070

[195] Hosaka K, Yang Y, Seki T, Fischer C, Dubey O, Fredlund E, et al. Pericyte–fibroblast transition promotes tumor growth and metastasis. Proc Natl Acad Sci USA. 2016. 10.1073/pnas.1608384113. cited 2026 Jan 28;113. 27608497 10.1073/pnas.1608384113 PMC5035870

[196] Feng F, Feng X, Zhang D, Li Q, Yao L. Matrix stiffness induces pericyte-fibroblast transition through YAP activation. Front Pharmacol. 2021;12:698275. 10.3389/fphar.2021.698275. 34135765 10.3389/fphar.2021.698275 PMC8202079

[197] Liao L, Wang Y-X, Fan S-S, Hu Y-Y, Wang X-C, Zhang X. The role and clinical significance of tumor-associated macrophages in the epithelial–mesenchymal transition of lung cancer. Front Oncol. 2025;15:1571583. 10.3389/fonc.2025.1571583. 40304000 10.3389/fonc.2025.1571583 PMC12037373

[198] Abaurrea A, Araujo AM, Caffarel MM. The Role of the IL-6 Cytokine family in epithelial-mesenchymal plasticity in cancer progression. Int J Mol Sci. 2021;22:8334. 10.3390/ijms22158334. 34361105 10.3390/ijms22158334 PMC8347315

[199] Li Z, Duan D, Li L, Peng D, Ming Y, Ni R, et al. Tumor-associated macrophages in anti-PD-1/PD-L1 immunotherapy for hepatocellular carcinoma: recent research progress. Front Pharmacol. 2024;15:1382256. 10.3389/fphar.2024.1382256. 38957393 10.3389/fphar.2024.1382256 PMC11217528

[200] Sieow JL, Gun SY, Wong SC. The sweet surrender: how myeloid cell metabolic plasticity shapes the tumor microenvironment. Front Cell Dev Biol. 2018;6:168. 10.3389/fcell.2018.00168. 30619850 10.3389/fcell.2018.00168 PMC6297857

[201] Tumino N, Fiore PF, Pelosi A, Moretta L, Vacca P. Myeloid derived suppressor cells in tumor microenvironment: Interaction with innate lymphoid cells. Semin Immunol. 2022;61–64:101668. 10.1016/j.smim.2022.101668. 36370673 10.1016/j.smim.2022.101668

[202] Qin S, Jiang J, Lu Y, Nice EC, Huang C, Zhang J, et al. Emerging role of tumor cell plasticity in modifying therapeutic response. Sig Transduct Target Ther. 2020;5:228. 10.1038/s41392-020-00313-5. 10.1038/s41392-020-00313-5 PMC7541492 33028808

[203] Stankic M, Pavlovic S, Chin Y, Brogi E, Padua D, Norton L, et al. TGF-β-Id1 signaling opposes Twist1 and promotes metastatic colonization via a mesenchymal-to-epithelial transition. Cell Rep. 2013;5:1228–42. 10.1016/j.celrep.2013.11.014. 24332369 10.1016/j.celrep.2013.11.014 PMC3891470

[204] Oh E, Hong J, Yun C-O. Regulatory T Cells induce metastasis by increasing Tgf-β and enhancing the epithelial–mesenchymal transition. Cells. 2019;8:1387. 10.3390/cells8111387. 31690033 10.3390/cells8111387 PMC6912455

[205] Lorenzo-Sanz L, Muñoz P. Tumor-infiltrating immunosuppressive cells in cancer-cell plasticity, tumor progression and therapy response. Cancer Microenviron. 2019;12:119–32. 10.1007/s12307-019-00232-2. 31583529 10.1007/s12307-019-00232-2 PMC6937363

[206] Chopin V, Lagadec C, Toillon R-A, Le Bourhis X. Neurotrophin signaling in cancer stem cells. Cell Mol Life Sci. 2016;73:1859–70. 10.1007/s00018-016-2156-7. 26883804 10.1007/s00018-016-2156-7 PMC11108437

[207] Taylor KR, Barron T, Hui A, Spitzer A, Yalçin B, Ivec AE, et al. Glioma synapses recruit mechanisms of adaptive plasticity. Nature. 2023;623:366–74. 10.1038/s41586-023-06678-1. 37914930 10.1038/s41586-023-06678-1 PMC10632140

[208] Reichardt LF. Neurotrophin-regulated signalling pathways. Philos Trans R Soc Lond B Biol Sci. 2006;361:1545–64. 10.1098/rstb.2006.1894. 16939974 10.1098/rstb.2006.1894 PMC1664664

[209] Ricci A, De Vitis C, Noto A, Fattore L, Mariotta S, Cherubini E, et al. TrkB is responsible for EMT transition in malignant pleural effusions derived cultures from adenocarcinoma of the lung. Cell Cycle Georget Tex. 2013;12:1696–703. 10.4161/cc.24759. 10.4161/cc.24759 PMC3713128 23656788

[210] Meng L, Liu B, Ji R, Jiang X, Yan X, Xin Y. Targeting the BDNF/TrkB pathway for the treatment of tumors (Review). Oncol Lett . 2018 10.3892/ol.2018.9854 cited 2026 Jan 30. 10.3892/ol.2018.9854 PMC6341806 30675270

[211] Wang S, Nie L, Song Y, Zhang F, Chen X, Shi W, et al. Neurturin promotes tumor cell motility and angiogenesis in colorectal cancer. Exp Cell Res. 2022;413:113049. 10.1016/j.yexcr.2022.113049. 35114191 10.1016/j.yexcr.2022.113049

[212] Zhang Y, Fu Q, Sun W, Yue Q, He P, Niu D, et al. Mechanical forces in the tumor microenvironment: roles, pathways, and therapeutic approaches. J Transl Med. 2025;23:313. 10.1186/s12967-025-06306-8. 40075523 10.1186/s12967-025-06306-8 PMC11899831

[213] Calvo F, Ege N, Grande-Garcia A, Hooper S, Jenkins RP, Chaudhry SI, et al. Mechanotransduction and YAP-dependent matrix remodelling is required for the generation and maintenance of cancer-associated fibroblasts. Nat Cell Biol. 2013;15:637–46. 10.1038/ncb2756. 23708000 10.1038/ncb2756 PMC3836234

[214] Belhabib I, Zaghdoudi S, Lac C, Bousquet C, Jean C. Extracellular matrices and cancer-associated fibroblasts: targets for cancer diagnosis and therapy? Cancers. 2021;13:3466. 10.3390/cancers13143466. 34298680 10.3390/cancers13143466 PMC8303391

[215] Eddy RJ, Weidmann MD, Sharma VP, Condeelis JS. Tumor cell invadopodia: invasive protrusions that orchestrate metastasis. Trends Cell Biol. 2017;27:595–607. 10.1016/j.tcb.2017.03.003. 28412099 10.1016/j.tcb.2017.03.003 PMC5524604

[216] Chen Z, Han F, Du Y, Shi H, Zhou W. Hypoxic microenvironment in cancer: molecular mechanisms and therapeutic interventions. Sig Transduct Target Ther. 2023;8:70. 10.1038/s41392-023-01332-8. 10.1038/s41392-023-01332-8 PMC9935926 36797231

[217] Balamurugan K. HIF -1 at the crossroads of hypoxia, inflammation, and cancer. Intl J Cancer. 2016;138:1058–66. 10.1002/ijc.29519. 10.1002/ijc.29519 PMC4573780 25784597

[218] Shaikat AH, Azad SMAK, Tamim MAR, Ullah MS, Amin MN, Sabbir MK, et al. Investigating hypoxia-inducible factor signaling in cancer: mechanisms, clinical implications, targeted therapeutic strategies, and resistance. Cancer Pathogenesis Therapy. 2025. 10.1016/j.cpt.2025.07.003. S2949713225000904. 41732210 10.1016/j.cpt.2025.07.003 PMC12925099

[219] Chang J, Pang EM, Adebowale K, Wisdom KM, Chaudhuri O. Increased stiffness inhibits invadopodia formation and cell migration in 3D. Biophys J. 2020;119:726–36. 10.1016/j.bpj.2020.07.003. 32697977 10.1016/j.bpj.2020.07.003 PMC7451915

[220] Esquer H, Zhou Q, Nemkov T, Abraham AD, Rinaldetti S, Chen Y-C, et al. Isolating and targeting the real-time plasticity and malignant properties of epithelial-mesenchymal transition in cancer. Oncogene. 2021;40:2884–97. 10.1038/s41388-021-01728-2. 33742123 10.1038/s41388-021-01728-2 PMC8944243

[221] Gollavilli PN, Parma B, Siddiqui A, Yang H, Ramesh V, Napoli F, et al. The role of miR-200b/c in balancing EMT and proliferation revealed by an activity reporter. Oncogene. 2021;40:2309–22. 10.1038/s41388-021-01708-6. 33654197 10.1038/s41388-021-01708-6 PMC7994202

[222] Rimmer N, Liang C-Y, Coelho R, Lopez MN, Jacob F. Generation of endogenously tagged E-cadherin cells using gene editing via non-homologous end joining. STAR Protoc. 2023;4:102305. 10.1016/j.xpro.2023.102305. 37178110 10.1016/j.xpro.2023.102305 PMC10199306

[223] Roberts B, Haupt A, Tucker A, Grancharova T, Arakaki J, Fuqua MA, et al. Systematic gene tagging using CRISPR/Cas9 in human stem cells to illuminate cell organization. Mol Biol Cell. 2017;28:2854–74. 10.1091/mbc.E17-03-0209. 28814507 10.1091/mbc.E17-03-0209 PMC5638588

[224] Lee S, Cieply B, Yang Y, Peart N, Glaser C, Chan P, et al. Esrp1-Regulated splicing of arhgef11 isoforms is required for epithelial tight junction integrity. Cell Rep. 2018;25:2417–e24305. 10.1016/j.celrep.2018.10.097. 30485810 10.1016/j.celrep.2018.10.097 PMC6371790

[225] Jolly MK, Preca B-T, Tripathi SC, Jia D, George JT, Hanash SM, et al. Interconnected feedback loops among ESRP1, HAS2, and CD44 regulate epithelial-mesenchymal plasticity in cancer. APL Bioeng. 2018;2:031908. 10.1063/1.5024874. 31069317 10.1063/1.5024874 PMC6324214

[226] Xiang J, Fu X, Ran W, Wang Z. Grhl2 reduces invasion and migration through inhibition of TGFβ-induced EMT in gastric cancer. Oncogenesis. 2017;6:e284. 10.1038/oncsis.2016.83. 28067907 10.1038/oncsis.2016.83 PMC5294246

[227] Hong T, Watanabe K, Ta CH, Villarreal-Ponce A, Nie Q, Dai X. An Ovol2-Zeb1 mutual inhibitory circuit governs bidirectional and multi-step transition between epithelial and mesenchymal states. PLoS Comput Biol. 2015;11:e1004569. 10.1371/journal.pcbi.1004569. 26554584 10.1371/journal.pcbi.1004569 PMC4640575

[228] Kumano K, Nakahashi H, Louphrasitthiphol P, Kuroda Y, Miyazaki Y, Shimomura O, et al. Hypoxia at 3D organoid establishment selects essential subclones within heterogenous pancreatic cancer. Front Cell Dev Biol. 2024;12:1327772. 10.3389/fcell.2024.1327772. 38374892 10.3389/fcell.2024.1327772 PMC10875002

[229] Zeng X, Ma Q, Li X-K, You L-T, Li J, Fu X, et al. Patient-derived organoids of lung cancer based on organoids-on-a-chip: enhancing clinical and translational applications. Front Bioeng Biotechnol. 2023;11:1205157. 10.3389/fbioe.2023.1205157. 37304140 10.3389/fbioe.2023.1205157 PMC10250649

[230] Duzagac F, Saorin G, Memeo L, Canzonieri V, Rizzolio F. Microfluidic organoids-on-a-chip: quantum leap in cancer research. Cancers (Basel). 2021;13:737. 10.3390/cancers13040737. 33578886 10.3390/cancers13040737 PMC7916612

[231] Fontana F, Marzagalli M, Sommariva M, Gagliano N, Limonta P. Vitro 3D Cultures to model the tumor microenvironment. Cancers (Basel). 2021;13:2970. 10.3390/cancers13122970. 34199324 10.3390/cancers13122970 PMC8231786

[232] Testa M, Gaggianesi M, D’Accardo C, Porcelli G, Turdo A, Di Marco C, et al. A Novel Tumor on chip mimicking the breast cancer microenvironment for dynamic drug screening. IJMS. 2025;26:1028. 10.3390/ijms26031028. 39940796 10.3390/ijms26031028 PMC11816644

[233] Bar-Hai N, Ben-Yishay R, Arbili-Yarhi S, Herman N, Avidan-Noy V, Menes T, et al. Modeling epithelial-mesenchymal transition in patient-derived breast cancer organoids. Front Oncol. 2024;14:1470379. 10.3389/fonc.2024.1470379. 39469640 10.3389/fonc.2024.1470379 PMC11513879

[234] Ajikumar A, Lei KF. Microfluidic technologies in advancing cancer research. micromachines (Basel). 2024;15:1444. 10.3390/mi15121444. 39770196 10.3390/mi15121444 PMC11677295

[235] Mehta P, Rahman Z, Ten Dijke P, Boukany PE. Microfluidics meets 3D cancer cell migration. Trends Cancer. 2022;8:683–97. 10.1016/j.trecan.2022.03.006. 35568647 10.1016/j.trecan.2022.03.006

[236] Guo B, Shi X, Ma Z, Ji M, Tang C, Wang F. A ratiometric dual luciferase reporter for quantitative monitoring of pre-mRNA splicing efficiency in vivo. J Biol Chem. 2021;297:100933. 10.1016/j.jbc.2021.100933. 34216622 10.1016/j.jbc.2021.100933 PMC8322121

[237] Chen Y, LeBleu VS, Carstens JL, Sugimoto H, Zheng X, Malasi S, et al. Dual reporter genetic mouse models of pancreatic cancer identify an epithelial-to-mesenchymal transition-independent metastasis program. EMBO Mol Med. 2018;10:e9085. 10.15252/emmm.201809085. 30120146 10.15252/emmm.201809085 PMC6180301

[238] Ilina O, Campanello L, Gritsenko PG, Vullings M, Wang C, Bult P, et al. Intravital microscopy of collective invasion plasticity in breast cancer. Dis Model Mech. 2018;11:dmm034330. 10.1242/dmm.034330. 29997220 10.1242/dmm.034330 PMC6176993

[239] Zhao Z, Zhu X, Cui K, Mancuso J, Federley R, Fischer K, et al. In vivo visualization and characterization of epithelial-mesenchymal transition in breast tumors. Cancer Res. 2016;76:2094–104. 10.1158/0008-5472.CAN-15-2662. 26893478 10.1158/0008-5472.CAN-15-2662 PMC4873431

[240] Karagiannis GS, Pastoriza JM, Borriello L, Jafari R, Coste A, Condeelis JS, et al. Assessing tumor microenvironment of metastasis doorway-mediated vascular permeability associated with cancer cell dissemination using intravital imaging and fixed tissue analysis. J Vis Exp. 2019. 10.3791/59633. 31305525 10.3791/59633 PMC6784529

[241] Sharma VP, Tang B, Wang Y, Duran CL, Karagiannis GS, Xue EA, et al. Live tumor imaging shows macrophage induction and TMEM-mediated enrichment of cancer stem cells during metastatic dissemination. Nat Commun. 2021;12:7300. 10.1038/s41467-021-27308-2. 34911937 10.1038/s41467-021-27308-2 PMC8674234

[242] Buschhaus JM, Gibbons AE, Luker KE, Luker GD. Fluorescence Lifetime Imaging of a Caspase-3 Apoptosis Reporter. Curr Protoc Cell Biol. 2017;77:21121–211212. 10.1002/cpcb.36. 10.1002/cpcb.36 PMC5729923 29227553

[243] Zhou F, Xing D, Wu S, Chen WR. Intravital imaging of tumor apoptosis with FRET probes during tumor therapy. Mol Imaging Biol. 2010;12:63–70. 10.1007/s11307-009-0235-y. 19543775 10.1007/s11307-009-0235-y PMC5987252

[244] Nobis M, Herrmann D, Warren SC, Kadir S, Leung W, Killen M, et al. A RhoA-FRET biosensor mouse for intravital imaging in normal tissue homeostasis and disease contexts. Cell Rep. 2017;21:274–88. 10.1016/j.celrep.2017.09.022. 28978480 10.1016/j.celrep.2017.09.022

[245] Hunter MV, Moncada R, Weiss JM, Yanai I, White RM. Spatially resolved transcriptomics reveals the architecture of the tumor-microenvironment interface. Nat Commun. 2021;12:6278. 10.1038/s41467-021-26614-z. 34725363 10.1038/s41467-021-26614-z PMC8560802

[246] Zheng Y, Schupp JC, Adams T, Clair G, Justet A, Ahangari F, et al. A deep generative model for deciphering cellular dynamics and in silico drug discovery in complex diseases. Nat Biomed Eng. 2025;9:2155–80. 10.1038/s41551-025-01423-7. 40542107 10.1038/s41551-025-01423-7 PMC12705450

[247] Pastushenko I, Brisebarre A, Sifrim A, Fioramonti M, Revenco T, Boumahdi S, et al. Identification of the tumour transition states occurring during EMT. Nature. 2018;556:463–8. 10.1038/s41586-018-0040-3. 29670281 10.1038/s41586-018-0040-3

[248] Katebi A, Ramirez D, Lu M. Computational systems-biology approaches for modeling gene networks driving epithelial-mesenchymal transitions. Comput Syst Oncol. 2021;1:e1021. 10.1002/cso2.1021. 34164628 10.1002/cso2.1021 PMC8219219

[249] Carstens JL, Lovisa S. Epithelial-to-mesenchymal transition drives cancer genomic instability. J Exp Clin Cancer Res. 2025;44:135. 10.1186/s13046-025-03402-x. 40301945 10.1186/s13046-025-03402-x PMC12042499

[250] Perelli L, Zhang L, Mangiameli S, Giannese F, Mahadevan KK, Peng F, et al. Evolutionary fingerprints of epithelial-to-mesenchymal transition. Nature. 2025;640:1083–92. 10.1038/s41586-025-08671-2. 40044861 10.1038/s41586-025-08671-2 PMC13537484

[251] Cava C, Colaprico A, Bertoli G, Graudenzi A, Silva TC, Olsen C, et al. SpidermiR: an R/bioconductor package for integrative analysis with miRNA Data. Int J Mol Sci. 2017;18:274. 10.3390/ijms18020274. 28134831 10.3390/ijms18020274 PMC5343810

[252] Subhadarshini S, Markus J, Sahoo S, Jolly MK. Dynamics of epithelial–mesenchymal plasticity: what have single-cell investigations elucidated so far? ACS Omega. 2023;8:11665–73. 10.1021/acsomega.2c07989. 37033874 10.1021/acsomega.2c07989 PMC10077445

[253] Sahoo S, Ramu S, Nair MG, Pillai M, San Juan BP, Milioli HZ et al. Multi-modal transcriptomic analysis unravels enrichment of hybrid epithelial/mesenchymal state and enhanced phenotypic heterogeneity in basal breast cancer. bioRxiv. 2023;2023.09.30.558960. 10.1101/2023.09.30.558960

[254] Matsuoka A, Shien K, Tomida S, Ohki M, Hisamatsu K, Fujiwara R, et al. Single-cell and spatial transcriptomic characterization of pulmonary pleomorphic carcinoma. Commun Biol. 2025;8:1773. 10.1038/s42003-025-09162-w. 41402584 10.1038/s42003-025-09162-w PMC12708732

[255] Puram SV, Tirosh I, Parikh AS, Patel AP, Yizhak K, Gillespie S, et al. Single-cell transcriptomic analysis of primary and metastatic tumor ecosystems in head and neck cancer. Cell. 2017;171:1611–e162424. 10.1016/j.cell.2017.10.044. 29198524 10.1016/j.cell.2017.10.044 PMC5878932

[256] Chakraborty P, George JT, Tripathi S, Levine H, Jolly MK. Comparative study of transcriptomics-based scoring metrics for the epithelial-hybrid-mesenchymal spectrum. Front Bioeng Biotechnol. 2020;8:220. 10.3389/fbioe.2020.00220. 32266244 10.3389/fbioe.2020.00220 PMC7100584

[257] Jia D, Li X, Bocci F, Tripathi S, Deng Y, Jolly MK, et al. Quantifying cancer epithelial-mesenchymal plasticity and its association with stemness and immune response. J Clin Med. 2019;8:725. 10.3390/jcm8050725. 31121840 10.3390/jcm8050725 PMC6572429

[258] Hari K, Ullanat V, Balasubramanian A, Gopalan A, Jolly MK. Landscape of epithelial-mesenchymal plasticity as an emergent property of coordinated teams in regulatory networks. Elife. 2022;11:e76535. 10.7554/eLife.76535. 36269057 10.7554/eLife.76535 PMC9683792

[259] Hari K, Harlapur P, Saxena A, Haldar K, Girish A, Malpani T, et al. Low dimensionality of phenotypic space as an emergent property of coordinated teams in biological regulatory networks. iScience. 2025;28:111730. 10.1016/j.isci.2024.111730. 39898023 10.1016/j.isci.2024.111730 PMC11787609

[260] Bocci F, Jia D, Nie Q, Jolly MK, Onuchic J. Theoretical and computational tools to model multistable gene regulatory networks. Rep Prog Phys Great Br. 2023;86. 10.1088/1361-6633/acec88. 10.1088/1361-6633/acec88 PMC10521208 37531952

[261] Slager J, Gatto F, Frey B, Shi W, Porebski B, Carreras-Puigvert J, et al. A morphology-based machine learning model for scoring epithelial-mesenchymal plasticity using organelle dynamics. Commun Biol. 2025;9:59. 10.1038/s42003-025-09326-8. 41372576 10.1038/s42003-025-09326-8 PMC12800221

[262] Cicceri G, Di Bella S, Di Franco S, Stassi G, Todaro M, Vitabile S. Deep learning approaches for morphological classification of intestinal organoids. IEEE Access. 2025;13:62267–87. 10.1109/ACCESS.2025.3558621.

[263] Poleszczuk J, Macklin P, Enderling H. Agent-based modeling of cancer stem cell driven solid tumor growth. Methods Mol Biol Clifton N J. 2016;1516:335–46. 10.1007/7651_2016_346. 10.1007/7651_2016_346 PMC6587968 27044046

[264] Sinha D, Saha P, Samanta A, Bishayee A. Emerging concepts of hybrid epithelial-to-mesenchymal transition in cancer progression. Biomolecules. 2020;10:1561. 10.3390/biom10111561. 33207810 10.3390/biom10111561 PMC7697085

[265] Alshahrani A, Alsubait A, Asiri Z, Alghamdi S, Bin Saqyah S, Alqahtani T, et al. Deciphering molecular pathways driving cancer invasion and metastasis: advances and therapeutic prospects. Front Oncol. 2025;15:1684896. 10.3389/fonc.2025.1684896. 41409250 10.3389/fonc.2025.1684896 PMC12705418

[266] Wang X, Xue X, Pang M, Yu L, Qian J, Li X, et al. Epithelial–mesenchymal plasticity in cancer: signaling pathways and therapeutic targets. MedComm. 2024;5:e659. 10.1002/mco2.659. 39092293 10.1002/mco2.659 PMC11292400

[267] Verstappe J, Skrypek N, De Coninck J, Soen B, Taminau J, Tatari M, et al. ZEB2 drives intra-tumor heterogeneity and skin squamous cell carcinoma formation with distinct EMP transition states. iScience. 2024;27:111169. 10.1016/j.isci.2024.111169. 39555401 10.1016/j.isci.2024.111169 PMC11567922

[268] Andriani F, Bertolini G, Facchinetti F, Baldoli E, Moro M, Casalini P, et al. Conversion to stem-cell state in response to microenvironmental cues is regulated by balance between epithelial and mesenchymal features in lung cancer cells. Mol Oncol. 2016;10:253–71. 10.1016/j.molonc.2015.10.002. 26514616 10.1016/j.molonc.2015.10.002 PMC5528953

[269] Bertolini G, Roz L, Perego P, Tortoreto M, Fontanella E, Gatti L, et al. Highly tumorigenic lung cancer CD133 + cells display stem-like features and are spared by cisplatin treatment. Proc Natl Acad Sci U S A. 2009;106:16281–6. 10.1073/pnas.0905653106. 19805294 10.1073/pnas.0905653106 PMC2741477

[270] Liu Q, Chen G, Moore J, Guix I, Placantonakis D, Barcellos-Hoff MH. Exploiting canonical TGFβ signaling in cancer treatment. Mol Cancer Ther. 2022;21:16–24. 10.1158/1535-7163.MCT-20-0891. 34670783 10.1158/1535-7163.MCT-20-0891 PMC8742762

[271] Ying X, Sun Y, He P. Bone morphogenetic protein-7 Inhibits EMT-associated genes in breast cancer. Cell Physiol Biochem. 2015;37:1271–8. 10.1159/000430249. 26431436 10.1159/000430249

[272] Na Y-R, Seok S-H, Kim D-J, Han J-H, Kim T-H, Jung H, et al. Bone morphogenetic protein 7 induces mesenchymal-to-epithelial transition in melanoma cells, leading to inhibition of metastasis. Cancer Sci. 2009;100:2218–25. 10.1111/j.1349-7006.2009.01301.x. 19735263 10.1111/j.1349-7006.2009.01301.x PMC11159605

[273] Veschi V, Mangiapane LR, Nicotra A, Di Franco S, Scavo E, Apuzzo T, et al. Targeting chemoresistant colorectal cancer via systemic administration of a BMP7 variant. Oncogene. 2020;39:987–1003. 10.1038/s41388-019-1047-4. 31591478 10.1038/s41388-019-1047-4 PMC6989400

[274] Rastegar-Pouyani N, Zare H, Rezaei F, Khazen S, Haji Abdolvahab M. Synergistic combination of pirfenidone and paclitaxel suppresses migration and stemness in triple-negative breast cancer: implications of EMT and pluripotency pathways. BMC Pharmacol Toxicol. 2026;27:9. 10.1186/s40360-025-01080-1. 41495762 10.1186/s40360-025-01080-1 PMC12784545

[275] Birchmeier C, Birchmeier W, Gherardi E, Vande Woude GF. Met, metastasis, motility and more. Nat Rev Mol Cell Biol. 2003;4:915–25. 10.1038/nrm1261. 14685170 10.1038/nrm1261

[276] Cañadas I, Rojo F, Taus Á, Arpí O, Arumí-Uría M, Pijuan L, et al. Targeting epithelial-to-mesenchymal transition with met inhibitors reverts chemoresistance in small cell lung cancer. Clin Cancer Res. 2014;20:938–50. 10.1158/1078-0432.CCR-13-1330. 24284055 10.1158/1078-0432.CCR-13-1330

[277] Neuzillet C, Tijeras-Raballand A, Cohen R, Cros J, Faivre S, Raymond E, et al. Targeting the TGFβ pathway for cancer therapy. Pharmacol Ther. 2015;147:22–31. 10.1016/j.pharmthera.2014.11.001. 25444759 10.1016/j.pharmthera.2014.11.001

[278] Akhurst RJ, Hata A. Targeting the TGFβ signalling pathway in disease. Nat Rev Drug Discov. 2012;11:790–811. 10.1038/nrd3810. 23000686 10.1038/nrd3810 PMC3520610

[279] Gjerdrum C, Tiron C, Høiby T, Stefansson I, Haugen H, Sandal T, et al. Axl is an essential epithelial-to-mesenchymal transition-induced regulator of breast cancer metastasis and patient survival. Proc Natl Acad Sci U S A. 2010;107:1124–9. 10.1073/pnas.0909333107. 20080645 10.1073/pnas.0909333107 PMC2824310

[280] Koorstra J-BM, Karikari CA, Feldmann G, Bisht S, Rojas PL, Offerhaus GJA, et al. The Axl receptor tyrosine kinase confers an adverse prognostic influence in pancreatic cancer and represents a new therapeutic target. Cancer Biol Ther. 2009;8:618–26. 10.4161/cbt.8.7.7923. 19252414 10.4161/cbt.8.7.7923 PMC2678175

[281] Byers LA, Diao L, Wang J, Saintigny P, Girard L, Peyton M, et al. An epithelial-mesenchymal transition gene signature predicts resistance to EGFR and PI3K inhibitors and identifies Axl as a therapeutic target for overcoming EGFR inhibitor resistance. Clin Cancer Res. 2013;19:279–90. 10.1158/1078-0432.CCR-12-1558. 23091115 10.1158/1078-0432.CCR-12-1558 PMC3567921

[282] Zhou BP, Deng J, Xia W, Xu J, Li YM, Gunduz M, et al. Dual regulation of Snail by GSK-3beta-mediated phosphorylation in control of epithelial-mesenchymal transition. Nat Cell Biol. 2004;6:931–40. 10.1038/ncb1173. 15448698 10.1038/ncb1173

[283] Jin X, Luan H, Chai H, Yan L, Zhang J, Wang Q, et al. Netrin–1 interference potentiates epithelial–to–mesenchymal transition through the PI3K/AKT pathway under the hypoxic microenvironment conditions of non–small cell lung cancer. Int J Oncol. 2019;54:1457–65. 10.3892/ijo.2019.4716. 30968155 10.3892/ijo.2019.4716

[284] Cassier PA, Navaridas R, Bellina M, Rama N, Ducarouge B, Hernandez-Vargas H, et al. Netrin-1 blockade inhibits tumour growth and EMT features in endometrial cancer. Nature. 2023;620:409–16. 10.1038/s41586-023-06367-z. 37532934 10.1038/s41586-023-06367-z PMC10412451

[285] Li L, Han R, Xiao H, Lin C, Wang Y, Liu H, et al. Metformin sensitizes EGFR-TKI-resistant human lung cancer cells in vitro and in vivo through inhibition of IL-6 signaling and EMT reversal. Clin Cancer Res. 2014;20:2714–26. 10.1158/1078-0432.CCR-13-2613. 24644001 10.1158/1078-0432.CCR-13-2613

[286] Onodera T, Sakai T, Hsu JC, Matsumoto K, Chiorini JA, Yamada KM. Btbd7 regulates epithelial cell dynamics and branching morphogenesis. Volume 329. New York, N.Y.: Science; 2010. pp. 562–5. 10.1126/science.1191880. 10.1126/science.1191880 PMC3412157 20671187

[287] Fang L-Z, Zhang J-Q, Liu L, Fu W-P, Shu J-K, Feng J-G, et al. Silencing of Btbd7 Inhibited Epithelial-Mesenchymal Transition and Chemoresistance in CD133^+^ Lung Carcinoma A549 Cells. oncol res. 2017;25:819–29. 10.3727/096504016X14772349843854. 27983936 10.3727/096504016X14772349843854 PMC7841122

[288] Yan H, Wang X, Mo Y, Huang Y, Fu Z, Xie L. Targeting the IGF1/Twist1 axis: A novel mechanism for β-elemene-induced anoikis and EMT inhibition in breast cancer cells. Biochimica et Biophysica Acta (BBA) -. Gen Subj. 2026;1870:130901. 10.1016/j.bbagen.2026.130901. 10.1016/j.bbagen.2026.130901 41490592

[289] Li M, Yang J, Wang K, Zhu A, Yin H, Li Z, et al. Taxifolin promotes glioma stem cell differentiation via CYP1B1-mediated EMT suppression. Phytomedicine. 2026;150:157735. 10.1016/j.phymed.2025.157735. 41485288 10.1016/j.phymed.2025.157735

[290] Vander Heiden MG, DeBerardinis RJ. Understanding the Intersections between metabolism and cancer biology. Cell. 2017;168:657–69. 10.1016/j.cell.2016.12.039. 28187287 10.1016/j.cell.2016.12.039 PMC5329766

[291] Ulanet DB, Couto K, Jha A, Choe S, Wang A, Woo H-K, et al. Mesenchymal phenotype predisposes lung cancer cells to impaired proliferation and redox stress in response to glutaminase inhibition. PLoS ONE. 2014;9:e115144. 10.1371/journal.pone.0115144. 25502225 10.1371/journal.pone.0115144 PMC4264947

[292] Sebestyén A, Lisanti MP, Ozsvari B. Novel metabolic approaches targeting cancer cells. Cancers (Basel). 2023;15:5448. 10.3390/cancers15225448. 38001707 10.3390/cancers15225448 PMC10670503

[293] Ramesh V, Brabletz T, Ceppi P. Targeting EMT in Cancer with repurposed metabolic inhibitors. Trends Cancer. 2020;6:942–50. 10.1016/j.trecan.2020.06.005. 32680650 10.1016/j.trecan.2020.06.005

[294] Wang G, Cao R, Wang Y, Qian G, Dan HC, Jiang W, et al. Simvastatin induces cell cycle arrest and inhibits proliferation of bladder cancer cells via PPARγ signalling pathway. Sci Rep. 2016;6:35783. 10.1038/srep35783. 27779188 10.1038/srep35783 PMC5078845

[295] Song M-Y, Lee D-Y, Yun S-M, Kim E-H. GLUT3 Promotes epithelial-mesenchymal transition via TGF-β/JNK/ATF2 signaling pathway in colorectal cancer cells. Biomedicines. 2022;10:1837. 10.3390/biomedicines10081837. 36009381 10.3390/biomedicines10081837 PMC9405349

[296] Ruscetti M, Dadashian EL, Guo W, Quach B, Mulholland DJ, Park JW, et al. HDAC inhibition impedes epithelial-mesenchymal plasticity and suppresses metastatic, castration-resistant prostate cancer. Oncogene. 2016;35:3781–95. 10.1038/onc.2015.444. 26640144 10.1038/onc.2015.444 PMC4896852

[297] Biersack B, Nitzsche B, Höpfner M. HDAC inhibitors with potential to overcome drug resistance in castration-resistant prostate cancer. Cancer Drug Resist Alhambra Calif. 2022;5:64–79. 10.20517/cdr.2021.105. 10.20517/cdr.2021.105 PMC8992583 35582529

[298] Drápela S, Bouchal J, Jolly MK, Culig Z, Souček K. ZEB1: A critical regulator of cell plasticity, dna damage response, and therapy resistance. Front Mol Biosci. 2020;7:36. 10.3389/fmolb.2020.00036. 32266287 10.3389/fmolb.2020.00036 PMC7096573

[299] Simanovich E, Oyelami F, Brockmeyer P, Rahat MA. The Anti-EMMPRIN monoclonal antibody hMR18-mAb induces tumor dormancy and inhibits the EMT process in human carcinoma cell lines co-cultured with macrophages. Biomedicines. 2025;13:2950. 10.3390/biomedicines13122950. 41462963 10.3390/biomedicines13122950 PMC12730406

[300] Wang Z, Li Y, Ahmad A, Azmi AS, Kong D, Banerjee S, et al. Targeting miRNAs involved in cancer stem cell and EMT regulation: an emerging concept in overcoming drug resistance. Drug Resist Updat. 2010;13:109–18. 10.1016/j.drup.2010.07.001. 20692200 10.1016/j.drup.2010.07.001 PMC2956795

[301] Ma J, Fang B, Zeng F, Ma C, Pang H, Cheng L, et al. Down-regulation of miR-223 reverses epithelial-mesenchymal transition in gemcitabine-resistant pancreatic cancer cells. Oncotarget. 2015;6:1740–9. 10.18632/oncotarget.2714. 25638153 10.18632/oncotarget.2714 PMC4359328

[302] Shi Z-D, Pang K, Wu Z-X, Dong Y, Hao L, Qin J-X, et al. Tumor cell plasticity in targeted therapy-induced resistance: mechanisms and new strategies. Sig Transduct Target Ther. 2023;8:113. 10.1038/s41392-023-01383-x. 10.1038/s41392-023-01383-x PMC10008648 36906600

[303] Li Z, Yin P. Tumor microenvironment diversity and plasticity in cancer multidrug resistance. Biochim et Biophys Acta (BBA) - Reviews Cancer. 2023;1878:188997. 10.1016/j.bbcan.2023.188997. 10.1016/j.bbcan.2023.188997 37832894

[304] Bughda R, Dimou P, D’Souza RR, Klampatsa A. Fibroblast activation protein (FAP)-Targeted CAR-T Cells: launching an attack on tumor stroma. Immunotargets Ther. 2021;10:313–23. 10.2147/ITT.S291767. 34386436 10.2147/ITT.S291767 PMC8354246

[305] Cao Z, Quazi S, Arora S, Osellame LD, Burvenich IJ, Janes PW, et al. Cancer-associated fibroblasts as therapeutic targets for cancer: advances, challenges, and future prospects. J Biomed Sci. 2025;32:7. 10.1186/s12929-024-01099-2. 39780187 10.1186/s12929-024-01099-2 PMC11715488

[306] Wu F, Yang J, Liu J, Wang Y, Mu J, Zeng Q, et al. Signaling pathways in cancer-associated fibroblasts and targeted therapy for cancer. Sig Transduct Target Ther. 2021;6:218. 10.1038/s41392-021-00641-0. 10.1038/s41392-021-00641-0 PMC8190181 34108441

[307] Lee HH, Al-Ogaili Z. Fibroblast activation protein and the tumour microenvironment: challenges and therapeutic opportunities. Oncol Rev. 2025;19:1617487. 10.3389/or.2025.1617487. 40741380 10.3389/or.2025.1617487 PMC12308159

[308] Liang J, Wang S, Zhang G, He B, Bie Q, Zhang B. A New antitumor direction: tumor-specific endothelial cells. Front Oncol. 2021;11:756334. 10.3389/fonc.2021.756334. 34988011 10.3389/fonc.2021.756334 PMC8721012

[309] Xu D, Li J, Li R-Y, Lan T, Xiao C, Gong P. PD-L1 Expression is regulated By NF-κB during EMT signaling in gastric carcinoma. Onco Targets Ther. 2019;12:10099–105. 10.2147/OTT.S224053. 31819504 10.2147/OTT.S224053 PMC6883928

[310] Shrestha R, Bridle KR, Crawford DHG, Jayachandran A. Immune checkpoint molecules are regulated by transforming growth factor (TGF)-β1-induced epithelial-to-mesenchymal transition in hepatocellular carcinoma. Int J Med Sci. 2021;18:2466–79. 10.7150/ijms.54239. 34104078 10.7150/ijms.54239 PMC8176170

[311] Fallon M, Sopata M, Dragon E, Brown MT, Viktrup L, West CR, et al. A Randomized placebo-controlled trial of the anti-nerve growth factor antibody tanezumab in subjects with cancer pain due to bone metastasis. Oncologist. 2023;28:e1268–78. 10.1093/oncolo/oyad188. 37343145 10.1093/oncolo/oyad188 PMC10712717

[312] Saloman JL, Singhi AD, Hartman DJ, Normolle DP, Albers KM, Davis BM. Systemic Depletion of nerve growth factor inhibits disease progression in a genetically engineered model of pancreatic ductal adenocarcinoma. Pancreas. 2018;47:856–63. 10.1097/MPA.0000000000001090. 29975347 10.1097/MPA.0000000000001090 PMC6044729

[313] Garajová I, Giovannetti E. Targeting Perineural Invasion in Pancreatic Cancer. Cancers (Basel). 2024;16:4260. 10.3390/cancers16244260. 39766161 10.3390/cancers16244260 PMC11674953

[314] Wang S, Nie L, Song Y, Zhang F, Chen X, Shi W, et al. Corrigendum to Neurturin promotes tumor cell motility and angiogenesis in colorectal cancer [Exp. Cell Res. 413 (2022) 113049]. Exp Cell Res. 2022;417:113206. 10.1016/j.yexcr.2022.113206. 35584556 10.1016/j.yexcr.2022.113206

[315] Tauriello DVF, Palomo-Ponce S, Stork D, Berenguer-Llergo A, Badia-Ramentol J, Iglesias M, et al. TGFβ drives immune evasion in genetically reconstituted colon cancer metastasis. Nature. 2018;554:538–43. 10.1038/nature25492. 29443964 10.1038/nature25492

[316] Gomes Alves Martins R, Tekin MM, Cragg MS, Roghanian A. Therapeutic targeting of tumour-associated macrophage receptors. Immunotherapy Adv. 2024;5:ltaf009. 10.1093/immadv/ltaf009. 10.1093/immadv/ltaf009 PMC12084764 40385641

[317] Su P, Li O, Ke K, Jiang Z, Wu J, Wang Y, et al. Targeting tumor–associated macrophages: Critical players in tumor progression and therapeutic strategies (Review). Int J Oncol. 2024;64:60. 10.3892/ijo.2024.5648. 38695252 10.3892/ijo.2024.5648 PMC11087038

[318] Wang Y, Jia A, Bi Y, Wang Y, Yang Q, Cao Y, et al. Targeting myeloid-derived suppressor cells in cancer immunotherapy. Cancers. 2020;12:2626. 10.3390/cancers12092626. 32942545 10.3390/cancers12092626 PMC7564060

[319] Griffin N, Faulkner S, Jobling P, Hondermarck H. Targeting neurotrophin signaling in cancer: the renaissance. Pharmacol Res. 2018;135:12–7. 10.1016/j.phrs.2018.07.019. 30031169 10.1016/j.phrs.2018.07.019

[320] Bruno F, Arcuri D, Vozzo F, Malvaso A, Montesanto A, Maletta R. Expression and signaling pathways of nerve growth factor (NGF) and Pro-NGF in breast cancer: a systematic review. Curr Oncol Tor Ont. 2022;29:8103–20. 10.3390/curroncol29110640. 10.3390/curroncol29110640 PMC9689427 36354700

[321] Forsyth PA, Krishna N, Lawn S, Valadez JG, Qu X, Fenstermacher DA, et al. p75 neurotrophin receptor cleavage by α- and γ-secretases is required for neurotrophin-mediated proliferation of brain tumor-initiating cells. J Biol Chem. 2014;289:8067–85. 10.1074/jbc.M113.513762. 24519935 10.1074/jbc.M113.513762 PMC3961639

[322] Ahn BY, Saldanha-Gama RFG, Rahn JJ, Hao X, Zhang J, Dang N-H, et al. Glioma invasion mediated by the p75 neurotrophin receptor (p75(NTR)/CD271) requires regulated interaction with PDLIM1. Oncogene. 2016;35:1411–22. 10.1038/onc.2015.199. 26119933 10.1038/onc.2015.199 PMC4800290

[323] Nomura A, Majumder K, Giri B, Dauer P, Dudeja V, Roy S, et al. Inhibition of NF-kappa B pathway leads to deregulation of epithelial-mesenchymal transition and neural invasion in pancreatic cancer. Lab Invest. 2016;96:1268–78. 10.1038/labinvest.2016.109. 27775688 10.1038/labinvest.2016.109 PMC5121017

[324] Boilly B, Faulkner S, Jobling P, Hondermarck H. Nerve dependence: from regeneration to cancer. Cancer Cell. 2017;31:342–54. 10.1016/j.ccell.2017.02.005. 28292437 10.1016/j.ccell.2017.02.005

[325] Rabben H-L, Zhao C-M, Hayakawa Y, Wang C, Chen T. Vagotomy and gastric tumorigenesis. CN. 2016;14:967–72. 10.2174/1570159X14666160121114854. 10.2174/1570159X14666160121114854 PMC5333586 26791481

[326] Aydin HB, Ozcelikkale A, Acar A. Exploiting matrix stiffness to overcome drug resistance. ACS Biomater Sci Eng. 2024;10:4682–700. 10.1021/acsbiomaterials.4c00445. 38967485 10.1021/acsbiomaterials.4c00445 PMC11322920

[327] Mai Z, Lin Y, Lin P, Zhao X, Cui L. Modulating extracellular matrix stiffness: a strategic approach to boost cancer immunotherapy. Cell Death Dis. 2024;15:307. 10.1038/s41419-024-06697-4. 38693104 10.1038/s41419-024-06697-4 PMC11063215

[328] Hu J, Tang Z, Beeraka NM, Xu R, Liu J, Zhao X, et al. Multi-pathway therapeutics in colorectal cancer: targeting EMT, CSCs, and non-apoptotic cell death for drug resistance reversal. J Drug Target. 2025;1–22. 10.1080/1061186X.2025.2600679. 10.1080/1061186X.2025.2600679 41367286

